# Sex, Racial, and Ethnic Representation in COVID-19 Clinical Trials: A Systematic Review and Meta-analysis

**DOI:** 10.1001/jamainternmed.2022.5600

**Published:** 2022-12-05

**Authors:** Hong Xiao, Riha Vaidya, Fang Liu, Ximing Chang, Xiaoqian Xia, Joseph M. Unger

**Affiliations:** 1 Public Health Sciences Division, Fred Hutchinson Cancer Center, Seattle, Washington; 2 Public Health Emergency Center, Chinese Center for Disease Control and Prevention, Beijing, China; 3 School of Public Health, Imperial College London, London, England, United Kingdom; 4 School of Nursing, Peking University Health Science Center, Beijing, China; 5 School of Public Health, London School of Hygiene and Tropical Medicine, London, England, United Kingdom

## Abstract

**Importance:**

Since the onset of the COVID-19 pandemic, there have been calls for COVID-19 clinical trials to be fully representative of all demographic groups. However, limited evidence is available about the sex, racial, and ethnic representation among COVID-19 prevention and treatment trials.

**Objective:**

To investigate whether female participants and racial and ethnic minority individuals are adequately represented in COVID-19 prevention and treatment trials in the US.

**Data Sources:**

Identified studies were registered on ClinicalTrials.gov or published in the PubMed database from October 2019 to February 2022.

**Study Selection:**

Included studies must have provided the number of enrolled participants by sex, race, or ethnicity. Only interventional studies conducted in the US for the primary purpose of the diagnosis, prevention, or treatment of (or supportive care for) COVID-19 conditions were included.

**Data Extraction and Synthesis:**

Data on counts of enrollments by demographic variables (sex, race, and ethnicity) and location (country and state) were abstracted. Studies were broadly categorized by primary purpose as prevention (including vaccine and diagnosis studies) vs treatment (including supportive care studies). A random effects model for single proportions was used. Trial estimates were compared with corresponding estimates of representation in the US population with COVID-19.

**Main Outcomes and Measures:**

Sex, racial, and ethnic representation in COVID-19 clinical trials compared with their representation in the US population with COVID-19.

**Results:**

Overall, 122 US-based COVID-19 clinical trials comprising 176 654 participants were analyzed. Studies were predominantly randomized trials (n = 95) for treatment of COVID-19 (n = 103). Sex, race, and ethnicity were reported in 109 (89.3%), 95 (77.9%), and 87 (71.3%) trials, respectively. Estimated representation in prevention and treatment trials vs the US population with COVID-19 was 48.9% and 44.6% vs 52.4% for female participants; 23.0% and 36.6% vs 17.7% for Hispanic or Latino participants; 7.2% and 16.5% vs 14.1% for Black participants; 3.8% and 4.6% vs 3.7% for Asian participants; 0.2% and 0.9% vs 0.2% for Native Hawaiian or Other Pacific Islander participants; and 1.3% and 1.4% vs 1.1% for American Indian or Alaska Native participants. Compared with expected rates in the COVID-19 reference population, female participants were underrepresented in treatment trials (85.1% of expected; *P* < .001), Black participants (53.7% of expected; *P* = .003) and Asian participants (64.4% of expected; *P* = .003) were underrepresented in prevention trials, and Hispanic or Latino participants were overrepresented in treatment trials (206.8% of expected; *P* < .001).

**Conclusions and Relevance:**

In this systematic review and meta-analysis, aggregate differences in representation for several demographic groups in COVID-19 prevention and treatment trials in the US were found. Strategies to better ensure diverse representation in COVID-19 studies are needed, especially for prevention trials.

## Key Points

**Question:**

Compared with their representation in the US population with COVID-19, are female participants and racial and ethnic minority persons underenrolled in COVID-19 prevention and treatment trials?

**Findings:**

In this systematic review and meta-analysis of 122 US-based COVID-19 clinical trials with 176 654 participants, female participants were underrepresented in treatment trials, Asian and Black participants were underrepresented in prevention trials, and Hispanic or Latino participants were overrepresented in treatment trials.

**Meaning:**

These findings show systemwide differences in representation for several key demographic groups in COVID-19 prevention and treatment trials in the US.

## Teaser

This systematic review and meta-analysis investigates whether female participants and racial and ethnic minority individuals are adequately represented in COVID-19 prevention and treatment trials in the US.

## Introduction

As of October 2022, 530 million people across the world had been infected by SARS-CoV-2, with 96 million cases and more than 1 million COVID-19–related deaths in the US alone.^1^ Evidence has repeatedly demonstrated disproportionately higher risk of COVID-19 incidence, hospitalization, and death in racial and ethnic minority groups.^2,3,4^ Gender- and sex-based differences in COVID-19 incidence and outcomes have also been shown.^5^ Moreover, these demographic domains have also been shown to be independent modulators of drug/vaccine efficacy and toxic effects in specific settings.^6,7,8,9,10^

Vaccines and drugs are usually approved based on established safety and efficacy through the rigorous conduct of randomized clinical trials.^11^ Prevention and treatment regimens shown to be effective in clinical trials cannot be confidently applied to all populations when individuals with diverse backgrounds are not adequately represented.^6^ However, clinical trials have often lacked equitable inclusion of female participants and individuals identifying as members of specific racial and ethnic groups, including Black, Hispanic, and Native American.^11,12^ Although the National Institutes of Health (NIH) and the US Food and Drug Administration developed plans to enhance the enrollment of underrepresented groups, diverse participation in trials has remained persistently low.^11,13,14^ This imbalance may have been exacerbated by the COVID-19 pandemic.^15^

Several calls urging that COVID-19 clinical trials be fully representative of all demographic groups have been published.^6,11,16,17,18^ To our knowledge, no study has comprehensively examined demographic representation across the landscape of both prevention and treatment COVID-19 clinical trials over the first 2 years of the pandemic. Given the need to ensure equitable access to trial participation for individuals of any background and the importance of sex, racial, and ethnic diversity in ensuring the validity, generalizability, and scientific rigor of clinical trials, we systematically reviewed the demographic representation of COVID-19 clinical trials in the US.

## Methods

### Selection of COVID-19 Clinical Trials

We searched trials registered in ClinicalTrials.gov or indexed in the PubMed database. For ClinicalTrials.gov, we retrieved all COVID-19 trials for which results had been posted as of February 18, 2022. Additionally, we searched the PubMed database for published COVID-19 trials using an established COVID-19 search string from October 31, 2019, to February 18, 2022.^19^ Three reviewers (H.X., X.C., X.X.) independently screened the titles, abstracts, and full text of articles to identify original publications of COVID-19 clinical trials. Only interventional studies (ie, those for which participants received any kind of nonbehavioral intervention) conducted in the US for the primary purpose of the diagnosis, prevention, or treatment of (or supportive care for) COVID-19 conditions were included. Differences among reviewers were resolved by consensus.

This study of published literature and publicly available data was exempt from institutional review approval. Preferred Reporting Items for Systematic Reviews and Meta-analyses (PRISMA) reporting guidelines were followed.^20^

### Data Extraction

Data on counts of enrollments by demographic variables (sex, race, and ethnicity) and location (country and state) were abstracted. For race, we used the following mutually exclusive categories based on commonly used federal classifications^21^: American Indian or Alaska Native, Asian, Black, Native Hawaiian or Other Pacific Islander, White, more than 1 race, other (undefined in the source), unknown, or missing. Ethnicity was classified as Hispanic or Latino vs non-Hispanic or non-Latino. Sex was classified as female, male, other (undefined in the source), unknown, or missing. Studies were broadly categorized by primary purpose as prevention (including vaccine and diagnosis studies) vs treatment (including supportive care studies). Additionally, we characterized studies by lead sponsor (NIH or other US federal agency vs industry vs all others [individuals, university, organizations]), sample size, trial type (randomized vs nonrandomized) and phase; studies recorded as combined phases (eg, phases 1/2) were categorized as the higher phase. Reference populations were derived from US population-based COVID-19 incidence data, extracted from COVID-19 Case Surveillance Public Use Data and, secondarily, US population data from the US Census Bureau Population Estimates Program.^22,23^

### Statistical Analysis

Proportional enrollment by sex, race, and ethnicity was determined by pooling study-specific estimates of proportions. Both random and fixed effects approaches were considered for deriving summary proportions. A statistically significant Cochran *Q* statistic or an *I*^2^ statistic greater than 50% indicates that a random effects model, which takes into account both within- and between-study variance, is preferable. The random effects model for single proportions was implemented in R, version 4.0.2 (R Foundation for Statistical Computing) using a restricted maximum-likelihood estimator.^24,25^ Only studies with more than 10 participants and conducted in the US (with US-based participants accounting for ≥75% of enrollments) were included. Trials reporting Hispanic or Latino ethnicity as a category of race were excluded from the analyses of racial or ethnic representation.

Individual study effects for each demographic domain were illustrated using forest plots.^26^ Results from trials grouped according to predefined study-level characteristics (primary purpose, trial phase, sponsor, and trial type) were separately analyzed. Odds ratios comparing trial participation between groups were derived using moderator analyses.^24^

The base case (primary) comparison for trial demographic representation estimates was to the corresponding proportion of individuals in the US population diagnosed with COVID-19, interpreted as a more appropriate determinant of representation across demographic groups than the general US population. Because more than 90% of the trials had been completed by April 2021, we used the cumulative incidence of COVID-19 as of April 2021 in calculating the COVID-19 population reference. Comparisons with the proportion of individuals in the US population (irrespective of their COVID-19 status) were also made. To aid interpretation, we calculated domain-specific estimates of the enrollment incidence disparity (EID), defined as the absolute difference in proportional representation between trial and reference population estimates. We also calculated the enrollment incidence ratio (EIR) as the ratio of study to reference population estimates.

For analyses of racial and ethnic representation, we also calculated “adjusted” population estimates of proportional representation by weighting according to the estimated proportion of trial participants in each state, to examine trial representation among states where the trials were actually conducted. Moderator analyses of representation by trial phase, primary funder, and trial type were conducted separately for prevention and treatment trials. Sensitivity analyses were performed using a “leave-one-out” procedure by serially excluding each of the individual studies and recalculating the overall estimates. We also performed a sensitivity analysis excluding trials that recruited children (<18 years old) or trials that were not exclusively conducted in the US. Two-sided *P* ≤ .05 was considered statistically significant.

## Results

### Trial Characteristics

**Figure 1.**
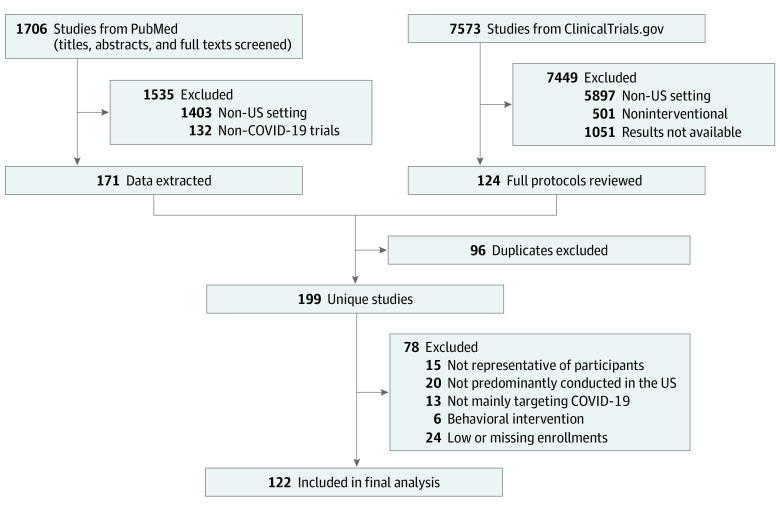
Selection of Studies Included in the Analysis

In total, 1706 studies were initially identified via the PubMed search engine. After exclusions, data were extracted from 171 articles (Figure 1). Using ClinicalTrials.gov, 7573 studies were identified, and after exclusions 124 full protocols were reviewed. After further exclusions (duplicates), 122 studies comprising 176 654 participants (including 159 214 participants [90.1%] from US sites) were analyzed. Trials were conducted in all 50 states except Alaska and Wyoming. The total number of trial sites ranged from 1 each in Delaware and North Dakota to 244 in Texas and 285 in California. We identified substantial heterogeneity (Cochran *Q* statistics with *P* < .05 and *I*^2^ > 50%) in trial effects across all demographic domains (eFigure in the Supplement), motivating the use of a random effects analysis.

Most trials examined treatments for COVID-19 (n = 99 [81.1%]), followed by COVID-19 vaccination trials (n = 14 [11.5%]; Table 1 and eTable 1 in the Supplement). Trials were predominantly phase 2 (n = 53 [43.4%]) or 3 (n = 34 [27.9%]). Six trials (4.9%), all prevention trials, were primarily funded by government agencies, 42 (34.4%) by industry, and 71 (58.2%) by other entities. Most trials (n = 95 [77.9%]) were randomized. The number of trial enrollments ranged from 11 to 44 047. Most trials (n = 98 [80.3%]) comprised fewer than 500 participants. The small number of trials (n = 4 [3.3%]) with sample sizes of 5000 participants or more were all large prevention trials representing 74.9% of all participants (n = 132 229).

**Table 1.** Study Characteristics

Characteristic	No. (%)					
	All trials		Prevention trials		Treatment trials	
	Trials (n = 122)	Participants (n = 176 654)	Trials (n = 19)	Participants (n = 140 147)	Trials (n = 103)	Participants (n = 36 497)
Phase						
1	10 (8.2)	853 (0.5)	3 (15.8)	347 (0.2)	7 (6.8)	506 (1.4)
2	53 (43.4)	40 628 (23.0)	5 (26.3)	33 908 (24.2)	48 (46.6)	6720 (18.4)
3	34 (27.9)	128 800 (72.9)	5 (26.3)	105 081 (75.0)	29 (28.2)	23 719 (65.0)
4	4 (3.3)	279 (0.2)	1 (5.3)	42 (<0.1)	3 (2.9)	237 (0.6)
Not applicable	21 (17.2)	6094 (3.4)	5 (26.3)	779 (0.6)	16 (15.5)	5315 (14.6)
Lead sponsor						
Government	6 (4.9)	3150 (1.8)	1 (5.3)	45 (<0.1)	5 (4.9)	3105 (8.5)
Industry	42 (34.4)	158 202 (89.6)	11 (57.9)	139 221 (99.3)	31 (30.1)	18 981 (52.0)
Other	71 (58.2)	15 180 (8.6)	7 (36.8)	891 (0.6)	64 (62.1)	14 289 (39.2)
Unknown	3 (2.5)	122 (0.1)	0	0	3 (2.9)	122 (0.3)
Allocation						
Randomized	95 (77.9)	170 731 (96.6)	12 (63.2)	139 263 (99.4)	83 (80.6)	31 468 (86.2)
Nonrandomized/single assignment	27 (22.1)	5923 (3.4)	7 (36.8)	894 (0.6)	20 (19.4)	5029 (13.8)
Sample size						
11-99	58 (47.5)	2531 (1.4)	6 (31.6)	355 (0.3)	52 (50.5)	2176 (6.0)
100-499	40 (32.8)	9662 (5.5)	6 (31.6)	1742 (1.2)	34 (33.0)	7920 (21.7)
500-999	9 (7.4)	2915 (1.7)	1 (5.3)	600 (0.4)	3 (2.9)	2315 (6.3)
1000-4999	11 (9.0)	26 598 (15.1)	2 (10.5)	5231 (3.7)	9 (8.7)	21 367 (58.5)
≥5000	4 (3.3)	132 229 (74.9)	4 (21.1)	132 229 (94.3)	0	0
US participants, %						
75-90	6 (4.9)	86 604 (49.0)	2 (10.5)	76 426 (54.5)	4 (3.9)	10 178 (27.9)
91-99	6 (4.9)	29 837 (16.9)	2 (10.5)	26 957 (19.2)	4 (3.9)	2880 (7.9)
100	110 (90.2)	60 213 (34.1)	15 (78.9)	36 334 (25.9)	95 (92.2)	23 439 (64.2)
Sex reported	109 (89.3)	169 130 (95.7)	14 (73.7)	139 376 (99.4)	95 (92.2)	29 754 (81.5)
Race						
Reported and included ethnicity as a category of race	17 (13.9)	3407 (1.9)	0	0	17 (16.5)	3407 (9.3)
Reported separately from ethnicity	78 (63.9)^a^	163 918 (92.8)	12 (63.2)	139 266 (99.4)	66 (64.1)	24 652 (67.5)
Ethnicity						
Reported as a category of race	17 (13.9)	3407 (1.9)	0	0	17 (16.5)	3407 (9.3)
Reported separately from race	70 (57.4)^b^	162 466 (92.0)	11 (57.9)	138 666 (98.9)	59 (57.3)	23 800 (65.2)

^a^ Overall estimates of representation by race category derived from these 78 studies.

^b^ Overall estimates of representation by ethnicity derived from these 70 studies.

Of the 122 trials, 109 (89.3%), 95 (77.9%), and 87 (71.3%) reported enrollment totals by sex, race, and ethnicity, respectively. Among the 95 and 87 trials that reported race and ethnicity, 78 (82.1%) and 70 (80.5%), respectively, reported Hispanic or Latino ethnicity as its own demographic category distinct from race.

### Sex Representation

**Figure 2.**
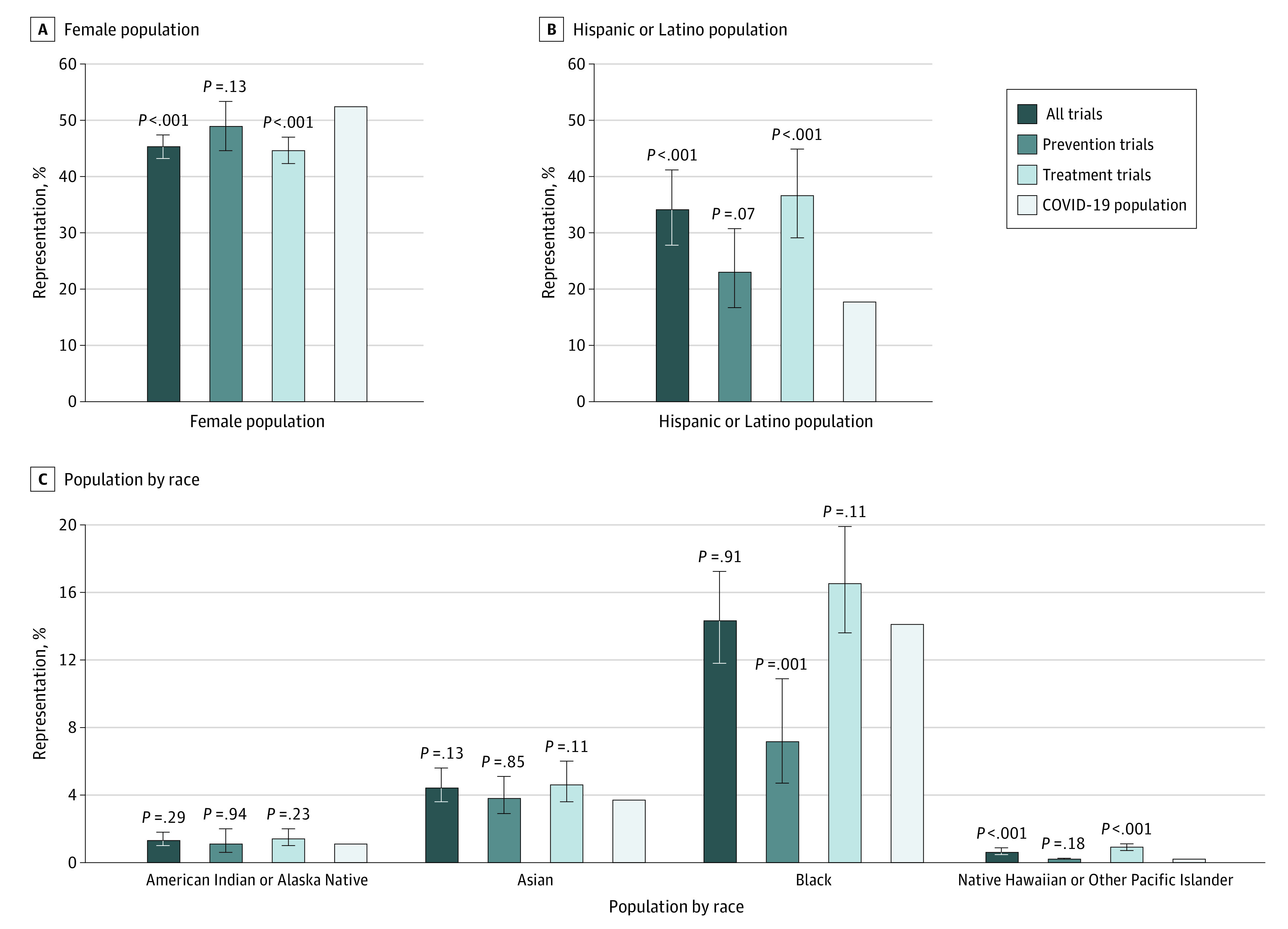
Differences in Population, Incidence, and Enrollment in COVID-19 Clinical Trials Error bars indicate 95% CIs.

**Figure 3.**
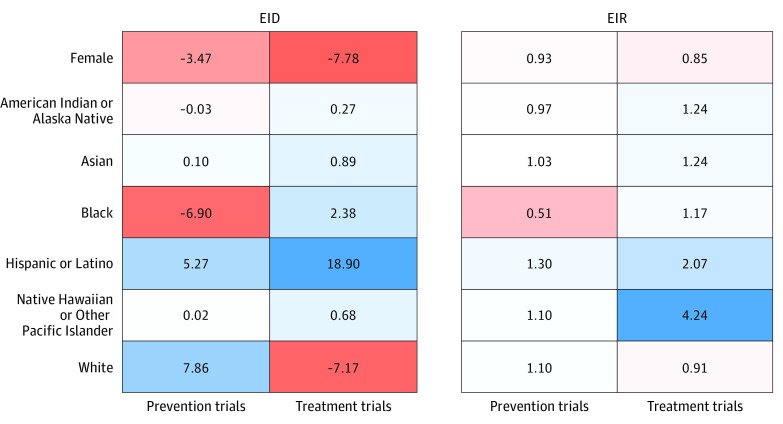
Heat Map of Enrollment Incidence Disparity (EID) and Enrollment Incidence Ratio (EIR) Favorable (more blue) and unfavorable (more red) disparity and ratio. EID is measured in percentage points.

Female participants represented 45.3% (95% CI, 43.2%-47.4%) of enrollees in all trials combined compared with 52.4% in the COVID-19 population (*P* < .001; Table 2). Female participants were well represented in prevention trials compared with the COVID-19 population (48.9% vs 52.4%; *P* = .13; EID = −3.5%; EIR = 0.93) but were underrepresented in treatment trials (44.6% vs 52.4%; *P* < .001; EID = −7.8%; EIR = 0.85; Figures 2 and 3 and Table 2). Female representation did not statistically significantly differ by trial phase and sponsor. Results were similar with the US population as the reference.

**Table 2.** Sex, Race, and Ethnicity Representation in COVID-19 Clinical Trials

Demographic domain^a^	No.	Estimated proportion of participants (95% CI), %	Effect of moderator		Proportion of cumulative COVID-19 incidence, %^b^	*P* value	Proportion of population, %	*P* value
			Odds ratio (95% CI)	*P* value				
**Female sex**								
Overall	109	45.3 (43.2-47.4)	NA	NA	52.4	<.001	50.5	<.001
Purpose								
Prevention	14	48.9 (44.5-53.4)	1.19 (0.97-1.46)	.10		.13		.48
Treatment	95	44.6 (42.3-47.0)	1 [Reference]	NA		<.001		<.001
Phase^c^								
1 or 2	59	45.1 (41.9-48.3)	1 [Reference]	NA		<.001		<.001
3 or 4	36	46.1 (43.2-49.1)	1.04 (0.87-1.25)	.06		<.001		<.001
Primary funder								
Government	6	43.0 (36.1-50.2)	0.88 (0.65-1.21)	.44		<.001		<.001
Industry	40	46.0 (43.2-48.9)	1 [Reference]	NA		<.001		<.001
Other	61	44.6 (40.3-47.9)	0.94 (0.79-1.13)	.52		<.001		<.001
**White race**								
Overall	78	73.9 (69.7-77.7)	NA	NA	77.9	.04	76.3	.22
Purpose								
Prevention	12	85.7 (80.3-89.9)	2.49 (1.60-3.88)	<.001		.007		<.002
Treatment	66	70.7 (66.1-74.9)	1 [Reference]	NA		.001		.008
Phase^c^								
1 or 2	48	75.3 (67.4-77.7)	1 [Reference]	NA		.36		.76
3 or 4	26	72.9 (67.4-77.7)	0.88 (0.59-1.31)	.54		.04		.18
Primary funder								
Government	5	74.0 (58.1-86.4)	0.77 (0.36-1.65)	.51		.57		.74
Industry	38	78.6 (74.0-82.6)	1 [Reference]	NA		.73		.31
Other	35	66.9 (59.3-73.7)	0.55 (0.36-0.82)	.005		<.001		.005
**Black race**								
Overall	78	14.3 (11.8-17.2)	NA	NA	14.1	.91	13.4	.53
Purpose								
Prevention	12	7.2 (4.7-10.9)	0.39 (0.23-0.66)	<.001		.001		.003
Treatment	66	16.5 (13.6-19.9)	1 [Reference]	NA		.11		.07
Phase^c^								
1 or 2	48	14.1 (10.6-18.5)	1 [Reference]	NA		.99		.73
3 or 4	26	13.1 (10.2-16.6)	0.92 (0.60-1.40)	.70		.54		.54
Primary funder								
Government	5	20.2 (17.0-23.7)	1.83 (1.29-2.62)	<.001		<.001		<.001
Industry	38	12.1 (9.4-15.4)	1 [Reference]	NA		.22		.42
Other	35	17.1 (12.4-23.1)	1.50 (0.94-2.40)	.09		.24		.14
**Asian race**								
Overall	78	4.4 (3.6-5.6)	NA	NA	3.7	.13	5.9	.06
Purpose								
Prevention	12	3.8 (2.9-5.1)	0.82 (0.55-1.23)	.35		.85		.003
Treatment	66	4.6 (3.6-6.0)	1 [Reference]	NA		.11		.07
Phase^c^								
1 or 2	48	3.7 (2.7-5.0)	1 [Reference]	NA		.96		.003
3 or 4	26	5.9 (4.3-8.1)	1.65 (1.03-2.64)	.04		.005		.98
Primary funder								
Government	5	11.8 (9.3-14.9)	3.26 (2.28-4.66)	<.001		<.001		<.001
Industry	38	4.0 (3.2-5.0)	1 [Reference]	NA		.63		<.001
Other	35	4.6 (2.9-7.0)	1.16 (0.69-1.94)	.58		.38		.24
**Native Hawaiian or Other Pacific Islander race**								
Overall	78	0.6 (0.5-0.9)	NA	NA	0.2	<.001	0.2	<.001
Purpose								
Prevention	12	0.2 (0.2-0.3)	0.26 (0.20-0.32)	<.001		.18		.03
Treatment	66	0.9 (0.7-1.1)	1 [Reference]	NA		<.001		<.001
Phase^c^								
1 or 2	48	1.1 (0.7-1.5)	1 [Reference]	NA		<.001		<.001
3 or 4	26	0.5 (0.3-0.7)	0.44 (0.20-0.37)	<.001		<.001		<.001
Primary funder								
Government	5	1.0 (0.6-1.7)	2.09 (1.05-4.09)	.03		<.001		<.001
Industry	38	0.5 (0.3-0.7)	1 [Reference]	NA		<.001		<.001
Other	35	0.8 (0.5-1.1)	1.53 (0.90-2.61)	.12		<.001		<.001
**American Indian or Alaska Native race**								
Overall	78	1.3 (1.0-1.8)	NA	NA	1.1	.29	1.3	.88
Purpose								
Prevention	12	1.1 (0.6-2.0)	0.78 (0.40-1.59)	.49		.94		.58
Treatment	66	1.4 (1.0-2.0)	1 [Reference]	NA		.23		.68
Phase^c^								
1 or 2	48	1.6 (1.1-2.4)	1 [Reference]	NA		.06		.24
3 or 4	26	1.1 (0.7-1.8)	0.68 (0.37-1.29)	.23		.98		.56
Primary funder								
Government	5	1.2 (0.8-1.7)	0.89 (0.51-1.52)	.66		.84		.57
Industry	38	1.3 (0.9-2.0)	1 [Reference]	NA		.45		.93
Other	35	1.3 (0.8-2.3)	1.01 (0.50-1.52)	.99		.56		.94
**Hispanic or Latino ethnicity**								
Overall	70	34.1 (27.8-41.1)	NA	NA	17.7	<.001	19.5	<.001
Purpose								
Prevention	11	23.0 (16.7-30.7)	0.52 (0.31-0.87)	.01		.07		.30
Treatment	59	36.6 (29.1-44.9)	1 [Reference]	NA		<.001		<.001
Phase^c^								
1 or 2	42	31.2 (22.2-41.9)	1 [Reference]	NA		.002		.008
3 or 4	24	33.9 (27.0-42.5)	1.13 (0.63-1.99)	.67		<.001		<.001
Primary funder								
Government	5	30.0 (18.6-44.5)	0.73 (0.36-1.52)	.38		.03		.07
Industry	35	37.1 (29.6-45.2)	1 [Reference]	NA		<.001		<.001
Other	30	30.3 (19.3-44.2)	0.74 (0.37-1.48)	.39		.02		.06

Abbreviation: NA, not applicable.

^a^ Race and ethnicity are presented in this order, rather than alphabetically, to better show the statistical representation.

^b^ As of April 2021.

^c^ Studies recorded as combined phases (eg, phases 1/2) were categorized as the higher phase.

### Racial Representation

Overall, Black representation was similar to the COVID-19 reference population (14.3% vs 14.1%; *P* = .91); however, representation differed by trial purpose, with Black participants being well represented in treatment trials (16.5% vs 14.1%; *P* = .11; EID = 2.4%; EIR = 1.17) but underrepresented in prevention trials (7.2% vs 14.1%; *P* = .001; EID = −5.7%; EIR = 0.77; Table 2 and Figure 3). Similarly, Asian participants were well represented overall when compared with the COVID-19 reference population (4.4% vs 3.7%; *P* = .13; EID = 0.7%; EIR = 1.18), including in both prevention trials (3.8% vs 3.7%; *P* = .85; EID = 0.1%; EIR = 1.03) and treatment trials (4.6% vs 3.7%; *P* = .11; EID = 0.9%; EIR = 1.24). However, Asian participants were underrepresented in prevention trials compared with the general US population (3.8% vs 5.9%; *P* = .003). Trials sponsored by the NIH were more likely to enroll Asian and Black participants compared with industry-sponsored trials (Asian participants: odds ratio, 3.27; *P* < .001; Black participants: odds ratio, 1.83; *P* < .001). Native Hawaiian or Other Pacific Islander participants were strongly overrepresented overall compared with the COVID-19 reference population (0.61% vs 0.21%; *P* < .001); however, this pattern was primarily observed in treatment trials (0.9% vs 0.2%; *P* < .001; EID = 0.7%; EIR = 4.24) and trials sponsored by the NIH (1.0% vs 0.2%; *P* < .001). Overall, American Indian or Alaska Native participants nearly matched the proportion in the COVID-19 population (1.3% vs 1.1%; *P* = .29), and the results were consistent when analyzed by primary purpose, trial phase, location, and sponsor (Table 2 and eTables 3 and 4 in the Supplement). Results were similar with the US population as the reference.

### Ethnic Representation

Overall, Hispanic or Latino representation was much greater in trials than in the COVID-19 reference population (34.1% vs 17.7%; *P* < .001). However, this pattern differed between prevention trials, where Hispanic or Latino representation was not statistically significantly different compared with the COVID-19 population (23.0% vs 17.7%; *P* = .07; EID = 5.3%; EIR = 1.30), and treatment trials, where Hispanic or Latino representation was much greater than in the COVID-19 population (36.6% vs 17.7%; *P* < .001; EID = 18.9%; EIR = 2.07). Hispanic or Latino participants remained overrepresented in treatment trials, all trials phases, and both industry- and university-sponsored trials. Results were similar with the US population as the reference.

### Sensitivity Analyses

The comparison to the adjusted proportion of the population reference led to consistent inferences, except for Asian participants (unadjusted, 4.6% vs 5.9%; *P* = .07 vs adjusted, 4.6% vs 7.0%; *P* = .002) in treatment trials compared with the US population (eTable 2 in the Supplement). Patterns of differences between key variables (ie, study phase and primary funder) were largely similar when moderation analyses were conducted separately for prevention and treatment and trials (eTable 2 in the Supplement). Patterns of differences between randomized and nonrandomized/single-arm trials were similar.

The use of a later landmark time (February 2022) as the COVID-19 population reference to compare with treatment trial representation also provided consistent findings (eTables 2 and 4 in the Supplement). When individual studies were iteratively excluded, nearly all of the overall estimates corresponded closely to the primary analysis (eTable 5 in the Supplement). The exclusion of trials not solely conducted among adults in the US showed consistent results with respect to female and Hispanic representation; differences in representation in trials for some racial subgroups were no longer statistically significant compared with the COVID-19 reference population (eTable 6 in the Supplement).

## Discussion

In this systematic review and meta-analysis, we found that sex, race, and ethnicity were reported in 89.3%, 77.9%, and 71.3% of US-based COVID-19 clinical trials, respectively. In COVID-19 prevention trials, Asian and Black participants were underrepresented, while Hispanic or Latino participants were overrepresented. In COVID-19 treatment trials, female participants were underrepresented, and Hispanic or Latino participants were overrepresented. These findings highlight the ongoing struggle in the US to provide equitable access to clinical studies regardless of an individual’s demographic background.

Despite the NIH’s efforts toward improving reporting of demographic data,^11,27^ sex, race, and ethnicity were not reported in numerous COVID-19 trials. Even when reported, 20% of studies did not follow the NIH’s recommendation to report race and ethnicity as independent categories. These findings contribute additional evidence to the underreporting of sex, race, and ethnic representation.^6,11,28,29^

Female participants have historically been underrepresented in clinical trials.^11,30^ The reasons include a reduced willingness to participate in clinical trials, differences in prognosis, perceived symptoms, and perceived greater risk of harm from interventions.^31,32,33,34,35,36,37^ In addition, pregnant women have routinely been excluded from clinical trials,^38^ and women of reproductive age have more concerns about the safety and efficacy of the treatments for themselves and their babies.^30,39^ Furthermore, evidence has shown that women were more adversely affected by the COVID-19 pandemic, including with respect to access to clinical trials and routine health care,^15,40,41^ potentially reflecting higher employment loss or increased household responsibilities and childcare.

Hispanic or Latino participants were overrepresented in COVID-19 trials, likely for multiple reasons. First, more than one-third of the US-based COVID-19 trial sites were in California, Florida, and Texas, which have large Hispanic or Latino populations. However, Hispanic or Latino participants remained overrepresented even after accounting for state-level differences in the distribution of trial sites and ethnic composition. Second, Hispanic or Latino representation in the COVID-19 reference population may have been underestimated by surveillance data, which rely on the assumption that missing ethnicity information is missing completely at random, a statistical assumption that may be invalid.^42^ Additionally, COVID-19 treatment trials were typically conducted among inpatient populations, which were likely disproportionately Hispanic or Latino owing to the relative lack of primary care services on contracting COVID-19 and the increased risk of COVID-19–associated hospitalization among Hispanic or Latino groups.^43,44^ The present findings about Hispanic or Latino representation stand in contrast to prior studies for COVID-19 and other diseases, which have found low enrollment of Hispanic or Latino populations in trials owing to institutional and/or systemic racism, distrust of the health care system, lack of access to clinical trial centers, low socioeconomic status, and language and communication barriers.^11,29,45,46,47,48,49,50^ The underrepresentation of Hispanic or Latino participants indicated in some previous studies may also be related to poor reporting of Hispanic or Latino identity when using administrative records.^49,51,52^ Understanding why Hispanic or Latino individuals were overrepresented in COVID-19 trials could aid in understanding ethnic disparities in participation in clinical trials for other diseases.^32^

Black participants were underrepresented in COVID-19 prevention trials, though not treatment trials; Asian participants were underrepresented in prevention trials compared with the general US population but not compared with the US COVID-19 population. Black patients with COVID-19 may be more likely to meet specific inclusion criteria (eg, currently hospitalized and requiring medical care for COVID-19) for treatment studies.^43,53^ More generally, the urgency of seeking treatment for actual disease may better ensure an encounter with the health care system that will more commonly result in participation in a clinical study, including for underrepresented groups. In the prevention setting, in contrast, this forcing mechanism will be absent, and the multilayered individual, social, and economic barriers to study participation that underrepresented groups often encounter are more likely to become manifest. These barriers include lack of access to health care services, difficulties traveling to health facilities, lengthy enrollment and follow-up requirements, and inadequate study enrollment opportunities associated with socioeconomic and cultural factors.^11,45,54^ Financial barriers may also play a role. Thus, reducing the direct financial burdens associated with participating in clinical trials by limiting or waiving co-payments and co-insurance could disproportionally benefit underrepresented groups. Similarly, providing support for indirect expenses such as transportation, childcare, and time off from work could be especially beneficial for underrepresented communities.^55^ Limited access to Black physicians could contribute to the underrepresentation of Black participants in vaccine trials because racial and ethnic minority groups are more likely to trust a physician from a background similar to their own.^56,57^ Additionally, lower trust in biomedical research has been well documented for both Asian and Black communities.^29,51,58,59,60^ The Asian American community in the US nearly doubled in size from 2000 (11.9 million) to 2019 (22.4 million).^61^ Given the cultural and linguistic diversity among Asian subpopulations, a strategy of partnering with language-concordant health care professionals, as well as improving strategic outreach to Asian communities, could help enhance knowledge about and interest in vaccine trials.^60,62^ Efforts to ensure improved inclusion in vaccine clinical trials may help to address mistrust and counter safety concerns about vaccine uptake.^32,63^ Future studies are needed to assess whether the lack of early community engagement and reduced racial representation in trials was associated with limited COVID-19 vaccination rates in selected populations.^11,32^

This analysis reaffirms prior evidence that industry-sponsored trials enrolled fewer racially diverse participants compared with federally sponsored trials.^31,64^ Industry-sponsored trials have not been subject to NIH mandates regarding proportional racial representation.^27^ Because the majority of COVID-19 vaccination and treatment trials are industry sponsored, and pharmaceutical companies contribute the most to production, marketing, and distribution of novel therapeutics and devices, poor representation of racial minority groups is of vital scientific interest and has unique implications for disparities in treatment efficacy, adverse effects, and access.^51,64^ Incentives such as tax breaks or patent extensions have been recommended for pharmaceutical companies to increase the inclusion of racial and ethnic minority participants in clinical trials.^62^ In recognition of generally poor racial representation in industry-sponsored trials, the US Food and Drug Administration published in April 2022 a draft guidance about the necessity for improved representation of underrepresented populations in industry-sponsored trials.^65^

### Strengths and Limitations

This study has several strengths. First, we assessed representation in both intervention and treatment trials from registration data sets and published literature. This is important because data from the ClinicalTrials.gov results database are typically more complete than peer-reviewed publications.^66^ However, the results of registered studies are sometimes published in journal articles before they are posted on ClinicalTrials.gov. The present inclusive search strategy mitigated such sampling bias, resulting in a large cohort of trials, which also allowed subgroup analyses to assess moderators and account for potential sources of confounding. Also, we compared the trial representation estimates to the US COVID-19 population, and secondarily, to the overall US population, to more fully contextualize these findings. Additionally, we used the weighted population and case population to better represent the population reference of the states where the clinical sites were located.

This review also had limitations. First, it is both an important finding and a limitation that a considerable proportion of studies did not report race and/or ethnicity or used customized race reporting that was not in compliance with reporting standards. Second, this analysis adjusted the population reference to account for the race and ethnicity composition of populations near trial sites but likely did not fully represent site catchment areas, as only state-level (rather than city- or county-level) population race and ethnicity data were available. Lastly, we only included studies predominantly conducted in the US. This strategy was advantageous for comparing diversity in COVID-19 trial cohorts with the US COVID-19 population, but it also limits the generalizability of these findings to other countries.

## Conclusions

Results of this systematic review and meta-analysis demonstrate that despite efforts to eliminate sex, racial, and ethnic disparities, gaps in reporting and differences in representation persisted in US-based COVID-19 trials. Additional strategies may be needed to ensure that all sponsors are accountable for appropriate representation of female participants and racial and ethnic minority individuals.

## Notes

Supplement. **eTable 1.** Included studies characteristics **eTable 2.** Sex and race/ethnicity representation in COVID-19 clinical trials **eTable 3.** Race/ethnicity representation in COVID-19 clinical trials: results from analyses stratified by locations of clinical trials (COVID-19 incidence as of April 2021) **eTable 4.** Race/ethnicity representation in COVID-19 clinical trials: results from analyses stratified by locations of clinical trials (COVID-19 incidence as of February 2022) **eTable 5.** Sensitivity analysis results for the estimates of female proportion, Hispanic/Latino ethnicity proportion, and racial proportions using the “leave one out” method **eTable 6.** Sex and race/ethnicity representation in COVID-19 clinical trials solely conducted among adults in the US **eFigure.** Forest plots of the study-level and summary estimates for each domain eReferences
