# Supplementary material for: Sex, Racial, and Ethnic Representation in COVID-19 Clinical Trials: A Systematic Review and Meta-analysis
Source: JAMA Intern Med. 2022 Dec 5;183(1):50–60. doi: 10.1001/jamainternmed.2022.5600 (PMC9857303; doi:10.1001/jamainternmed.2022.5600)
Supplement: Supplement. — eTable 1. Included studies characteristics eTable 2. Sex and race/ethnicity representation in COVID-19 clinical trials eTable 3. Race/ethnicity representation in COVID-19 clinical trials: results from analyses stratified by locations of clinical trials (COVID-19 incidence as of April 2021) eTable 4. Race/ethnicity representation in COVID-19 clinical trials: results from analyses stratified by locations of clinical trials (COVID-19 incidence as of February 2022) eTable 5. Sensitivity analysis results for the estimates of female proportion, Hispanic/Latino ethnicity proportion, and racial proportions using the “leave one out” method eTable 6. Sex and race/ethnicity representation in COVID-19 clinical trials solely conducted among adults in the US eFigure. Forest plots of the study-level and summary estimates for each domain eReferences [file jamainternmed-e225600-s001.pdf]

Supplemental Online Content

Xiao H, Vaidya R, Liu F, Chang X, Xia X, Unger JM. Sex, racial, and ethnic representation in COVID-19 clinical trials: a systematic review and meta-analysis. *JAMA Intern Med*. Published online December 5, 2022. doi:10.1001/jamainternmed.2022.5600

eTable 1. Included studies characteristics
eTable 2. Sex and race/ethnicity representation in COVID-19 clinical trials
eTable 3. Race/ethnicity representation in COVID-19 clinical trials: results from analyses stratified by locations of clinical trials (COVID-19 incidence as of April 2021)
eTable 4. Race/ethnicity representation in COVID-19 clinical trials: results from analyses stratified by locations of clinical trials (COVID-19 incidence as of February 2022)
eTable 5. Sensitivity analysis results for the estimates of female proportion, Hispanic/Latino ethnicity proportion, and racial proportions using the “leave one out” method
eTable 6. Sex and race/ethnicity representation in COVID-19 clinical trials solely conducted among adults in the US
eFigure. Forest plots of the study-level and summary estimates for each domain
eReferences

This supplemental material has been provided by the authors to give readers additional information about their work.

© 2022 Xiao H et al. *JAMA Intern Med.*

eTable 1. Included studies characteristics

Sources	Interventions	Lead sponsor	Primary Purpose	Child included	Phases	Randomized	Sex reported	Race Reported	Ethnicity reported	No. Sites (Enrollment)	US based sites (Enrollment)
Abella et al., 2021[1]	Drug：Hydroxychloroquine	Other	treatment	No	Phase 2	Yes	Yes	Yes	Not distinct from race	1 (132)	1 (132)
Ali et al., 2021[2]	Biological: mRNA1273 SARSCoV2 vaccine from Moderna	Industry	prevention	Yes	Phase 2|3	Yes	Yes	Yes	Distinct from Race	46 (3726)	46 (3726)
Bar et al., 2021[3]	Biological: Convalescent Plasma	Other	treatment	No	Phase 1	Yes	Yes	Yes	Distinct from Race	1 (79)	1 (79)
Barnabas et al., 2021[4]	Drug：Hydroxychloroquine	Other	treatment	No	Phase 2|3	Yes	Yes	Yes	Distinct from Race	8 (689)	8 (689)
Bennett-Guerrero et al., 2021[5]	Biological: Convalescent Plasma<br>Biological: Standard Donor Plasma	Other	treatment	No	Phase 1|2	Yes	Yes	Yes	No	1 (74)	1 (74)
Bikdeli et al., 2022[6]	Drug: heparin	Industry	Treatment		not Applicable	Yes	No	No	No	(562)	(562)
Bradfute et al., 2020[7]	Drug: Convalescent Plasma	Other	treatment	No	Phase 2	No	Yes	Yes	Distinct from Race	1 (12)	1 (12)
Chigutsa et al., 2021[8]	Drug: LY3819253<br>Drug: LY3832479<br>Drug: Placebo<br>Drug: VIR7831<br>Drug: LY3853113	Industry	Treatment	Yes	Phase 2	Yes	No	No	No	164 (2970)	161 (2970)
Chu et al., 2021[9]	Biological: mRNA1273 SARSCoV2 vaccine	Industry	prevention	No	Phase 2	Yes	Yes	Yes	No	8 (600)	8 (600)
Dayya et al., 2021[10]	Device: Oxygen Hood	Other	treatment	Yes	Not Applicable	Yes	Yes	No	No	1 (136)	1 (136)
Donato et al., 2021[11]	Biologocal:Convalescent Plasma	Other	treatment		Phase 2	No	Yes	Yes	Not distinct from race	1 (51)	1 (51)
Dunkle LM., 2021[12]	Biological: SARSCoV2 rS/MatrixM1 Adjuvant (Initial Vaccination Period)	Industry	prevention	No	Phase 3	Yes	Yes	Yes	Distinct from Race	156 (25452)	146 (23932)
Elamir et al., 2022[13]	Drug：calcitriol		treatment			Yes	Yes	Yes	Not distinct from race	3 (50)	3 (50)
Falsey et al., 2021[14]	Biological：AZD1222 (ChAdOx1 nCoV19) Covid19 vaccine	Industry	prevention	No	Phase 2	Yes	Yes	Yes	Distinct from Race	84 (32379)	82 (28717)
Ghandehari et al., 2021[15]	Drug: Progesterone 100 MG	Other	treatment	No	Phase 1	Yes	No	Yes	Distinct from Race	1 (40)	1 (40)
Goepfert et al., 2021[16]	Biological：CoV2 preS dTM vaccine	Industry	prevention	No	Phase 1|2	Yes	Yes	Yes	Distinct from Race	11 (441)	11 (441)
Greenbaum et al., 2021[17]	Biological: COVID19 convalescent plasma	Other	treatment	No		No	Yes	Yes	No	12 (44)	12 (44)
Gupta et al., 2021[18]	Biological: VIR7831	Industry	treatment	No	Phase 3	Yes	Yes	Yes	Distinct from Race	91 (583)	62 (538)
Hess et al., 2021[19]	Device：lowdose, wholelung radiation therapy (LDRT)	Other	treatment	No	Phase 1|2	No	Yes	Yes	Distinct from Race	2 (20)	2 (20)

© 2022 Xiao H et al. *JAMA Intern Med.*

Huang et al., 2021[20]	Drug：chlorhexidine	Other	treatment			Yes	Yes	No	No	4 (294)	4 (294)
Humeniuk et al., 2020[21]	Drug：Remdesivir	Industry	treatment	No	Phase 1	Yes	Yes	Yes	Distinct from Race	1 (120)	1 (120)
Jackson et al., 2020[22]	Biological：mRNA1273 vaccine	NIH	prevention	No	Phase 1	No	Yes	Yes	Distinct from Race	3 (45)	3 (45)
Kaur et al., 2021[23]	Device: high flow nasal cannula (HFNC)<br>Procedure: Prone positioning (PP)	Other	treatment	No	Not Applicable	Yes	Yes	Yes	Not distinct from race	1 (125)	1 (125)
Kimura et al., 2020[24]	Device：Nasal saline irrigation		treatment			Yes	No	No	No	1 (45)	1 (45)
Lanzoni et al., 2021[25]	Biological: Umbilical Cord Mesenchymal Stem Cells + Heparin along with best supportive care.<br>Other: Vehicle + Heparin along with best supportive care	Other	treatment	No	Phase 1|2	Yes	Yes	Yes	Distinct from Race	1 (24)	1 (24)
Lerner et al., 2020[26]	Drug：omega3 fatty acid supplementation	Other	treatment	No	Phase 2	Yes	No	No	No	1 (176)	1 (176)
Liu et al., 2021[27]	Drug：Hydroxychloroquine	Other	prevention	No	Phase 4	Yes	No	No	No	3 (42)	3 (42)
Lundgren et al., 2021[28]	Biological: LY3819253Drug: PlaceboBiological: RemdesivirBiological: VIR7831Biological: BRII196/BRII198Biological: AZD7442Drug: MP0420Drug: PF07304814	Other	treatment	No	Phase 3	Yes	Yes	Yes	Not distinct from race	147 (314)	98 (250)
Miller et al., 2020[29]	Drug：Auxora	Industry	treatment	No	Phase 2	Yes	Yes	Yes	Distinct from Race	17 (30)	17 (30)
Nickel et al., 2020[30]	Drug: Hydroxyurea	Other	treatment	Yes	Phase 2	No	No	No	No	1 (14)	1 (14)
O'Brien et al., 2021[31]	Drug：REGENCOV+REGN10987	Industry	prevention	No	Phase 3	Yes	Yes	Yes	Distinct from Race	134 (1505)	132 (1471)
Perepu et al., 2021[32]	Drug: Intermediate dose thromboprophylaxis<br>Drug: Standard of Care thromboprophylaxis	Other	treatment	No	Phase 4	Yes	Yes	Yes	Not distinct from race	2 (173)	2 (173)
Baden et al., 2021[33]	Biological：The mRNA1273 vaccine	Industry	prevention	No	Phase 3	Yes	Yes	Yes	Distinct from Race	100 (30351)	100 (30351)
Salazar et al., 2020[34]	drug: convalescent plasma	NIH	treatment			No	Yes	No	No	1 (25)	1 (25)
Sengupta et al., 2020[35]	drug: Exosomes Derived from Bone Marrow Mesenchymal Stem Cells		treatment	No		No	Yes	Yes	Not distinct from race	1 (27)	1 (27)
Shah et al., 2021[36]	Drug: Standard of Care (SOC) and Colchicine+Rosuvastatin	Other	Treatment	No	Phase 3	Yes	No	No	No	4 (236)	4 (236)
Shroff RT et al[37]	Biological：BNT162b2 mRNA Covid19 vaccine	Other	prevention	No	Phase 1	No	Yes	No	No	1 (70)	1 (70)
Skipper et al., 2020[38]	drug：Hydroxychloroquine	Other	treatment		Phase 3	Yes	Yes	No	No	5 (423)	2 (385)
Strohbehn et al., 2021[39]	drug：Tocilizumab	Other	treatment	No	Phase 2	No	Yes	Yes	Not distinct from race	1 (32)	1 (32)
Taylor et al., 2021[40]	Therapeutic strategy：Prone positioning	Other	treatment			Yes	Yes	Yes	Distinct from Race	1 (80)	1 (80)

© 2022 Xiao H et al. *JAMA Intern Med.*

Thomas et al., 2021[41]	Dietary Supplement: Ascorbic Acid<br>Dietary Supplement: Zinc Gluconate<br>Dietary Supplement: Ascorbic Acid and Zinc Gluconate<br>Other: Standard of Care	Other	supportive care	No		Yes	Yes	Yes	No	2 (214)	2 (214)
Thomas SJ., 2021[42]	Biological: BNT162b2 mRNA Covid19 Vaccine	Industry	prevention	No	Phase 2|3	Yes	Yes	Yes	Distinct from Race	166 (44047)	144 (33586)
Uckun et al., 2021[43]	drug (RJX)	Industry	treatment	No	Phase 1	Yes	Yes	Yes	No	1 (76)	1 (76)
Wang et al., 2021[44]	drug： vitamin D	Other	treatment	No		Yes	No	No	No	(2700)	(2700)
Weinreich et al., 2021[45]	Drug: casirivimab+imdevimab combination therapy	Industry	Treatment	Yes	Phase 3	No	Yes	Yes	Distinct from Race	114 (4057)	102 (3600)
NCT03852537[46]	Drug: Methylprednisolone|Other: Usual Care	Other	treatment	No	Phase 2	Yes	Yes	Yes	No	1 (44)	1 (44)
NCT04280705[47]	Other: Placebo|Drug: Remdesivir	NIH	treatment	No	Phase 3	Yes	Yes	Yes	Distinct from Race	60 (1062)	41 (1062)
NCT04292899[48]	Drug: Remdesivir|Drug: Standard of Care	Industry	treatment	Yes	Phase 3	Yes	Yes	Yes	Distinct from Race	183 (4838)	106 (3878)
NCT04308668[49]	Drug: Hydroxychloroquine|Other: Placebo	Other	treatment	No	Phase 3	Yes	Yes	Yes	Distinct from Race	5 (1312)	2 (1253)
NCT04315298[50]	Drug: Sarilumab|Drug: Placebo	Industry	treatment	No	Phase 2|3	Yes	Yes	Yes	Distinct from Race	62 (1912)	62 (1912)
NCT04317040[51]	Drug: CD24Fc|Drug: Placebo	Industry	treatment	No	Phase 3	Yes	Yes	Yes	Distinct from Race	10 (234)	10 (234)
NCT04320615[52]	Drug: Tocilizumab (TCZ)|Drug: Placebo	Industry	treatment	No	Phase 3	Yes	Yes	Yes	Distinct from Race	62 (438)	23 (438)
NCT04328467[53]	Drug: Hydroxychloroquine|Other: Placebo	Other	treatment	No	Phase 3	Yes	Yes	Yes	Not distinct from race	2 (1483)	2 (1483)
NCT04331899[54]	Drug: Peginterferon Lambda1a|Other: Placebo	Other	treatment	No	Phase 2	Yes	Yes	Yes	Not distinct from race	1 (120)	1 (120)
NCT04332081[55]	Device: hyperbaric oxygen therapy (HBOT)	Other	treatment	No	Not Applicable	No	Yes	No	No	4 (80)	4 (80)
NCT04332107[56]	Drug: Azithromycin|Drug: Placebos	Other	treatment	No	Phase 3	Yes	Yes	Yes	Not distinct from race	1 (263)	1 (263)
NCT04332991[57]	Drug: Hydroxychloroquine|Drug: Placebo	Other	treatment	No	Phase 3	Yes	Yes	Yes	Not distinct from race	40 (479)	40 (479)
NCT04335552[58]	Other: Standard of care|Drug: Hydroxychloroquine|Drug: Azithromycin	Other	treatment	Yes	Phase 2	Yes	Yes	Yes	Distinct from Race	4 (11)	4 (11)
NCT04338009[59]	Other: Discontinuation of ARB/ACEI|Other: Continuation of ARB/ACEI	Other	treatment	No	Not Applicable	Yes	Yes	No	Distinct from Race	1 (152)	1 (152)

© 2022 Xiao H et al. *JAMA Intern Med.*

NCT04340557[60]	Drug: Losartan	Other	prevention drug (infected people)	No	Phase 4	Yes	Yes	Yes	Not distinct from race	3 (31)	3 (31)
NCT04342663[61]	Drug: Fluvoxamine|Drug: Placebo	Other	treatment	No	Phase 2	Yes	Yes	Yes	Distinct from Race	2 (152)	2 (152)
NCT04342897[62]	Drug: LY3127804|Drug: Placebo	Industry	treatment	No	Phase 2	Yes	Yes	Yes	Distinct from Race	13 (95)	13 (95)
NCT04343261[63]	Biological: Convalescent Plasma	Other	treatment	No	Phase 2	No	Yes	Yes	Distinct from Race	1 (48)	1 (48)
NCT04343989[64]	Drug: Clazakizumab 25 mg|Drug: Clazakizumab 12.5 mg|Other: Placebo	Other	treatment	No	Phase 2	Yes	Yes	Yes	Distinct from Race	2 (178)	2 (178)
NCT04347954[65]	Drug: PovidoneIodine 2%|Drug: PovidoneIodine 0.5%|Drug: Isotonic saline 0.9%	Other	treatment	No	Phase 1|2	Yes	Yes	Yes	Not distinct from race	1 (35)	1 (35)
NCT04348500[66]	Drug: Clazakizumab	Other	treatment	No	Phase 2	Yes	Yes	Yes	Distinct from Race	1 (16)	1 (16)
NCT04349098[67]	Drug: Selinexor|Other: Placebo	Industry	treatment	No	Phase 2	Yes	Yes	Yes	Distinct from Race	33 (190)	20 (190)
NCT04351243[68]	Drug: Gimsilumab|Drug: Placebo	Industry	prevention	No	Phase 2	Yes	Yes	Yes	Distinct from Race	33 (225)	33 (225)
NCT04353037[69]	Drug: Group 1 HCQ|Drug: Group 2 Placebo	Industry	treatment	No	Phase 2	Yes	Yes	Yes	Distinct from Race	1 (39)	1 (39)
NCT04354870[70]	Drug: Hydroxychloroquine (HCQ)	Other	treatment	No	Phase 2	No	Yes	Yes	Distinct from Race	1 (49)	1 (49)
NCT04355767[71]	Biological: Convalescent Plasma|Biological: Saline	Other	treatment	No	Phase 3	Yes	Yes	Yes	Distinct from Race	53 (511)	53 (511)
NCT04356937[72]	Drug: Tocilizumab|Drug: Placebos	Other	treatment	No	Phase 3	Yes	Yes	Yes	Distinct from Race	3 (243)	3 (243)
NCT04357730[73]	Drug: Alteplase 50 MG [Activase]	Other	treatment	No	Phase 2	Yes	Yes	Yes	Distinct from Race	9 (50)	9 (50)
NCT04358068[74]	Drug: Hydroxychloroquine (HCQ)|Drug: Azithromycin (Azithro)|Drug: Placebo for Hydroxychloroquine|Drug: Placebo for Azithromycin	NIH	treatment	No	Phase 2	Yes	Yes	Yes	Distinct from Race	11 (16)	11 (16)
NCT04358081[75]	Drug: HCQ|Drug: HCQ+AZT|Drug: Placebo	Industry	treatment	No	Phase 3	Yes	Yes	Yes	No	7 (19)	7 (19)
NCT04359797[76]	Other: Prone|Other: Usual Care	Other	supportive care	No	Not Applicable	Yes	Yes	Yes	Distinct from Race	2 (501)	2 (501)
NCT04363437[77]	Drug: Colchicine|Drug: Usual Care	Other	treatment	No	Phase 2	Yes	Yes	No	No	1 (21)	1 (21)
NCT04363736[78]	Drug: Tociliuzumab	Industry	treatment	No	Phase 2	Yes	Yes	Yes	Distinct from Race	24 (97)	24 (97)
NCT04365153[79]	Drug: Canakinumab Injection 600mg|Drug: Canakinumab Injection 300mg|Drug: Placebos	Other	treatment	No	Phase 2	Yes	Yes	Yes	Not distinct from race	2 (45)	2 (45)
NCT04365699[80]	Drug: AT001	Other	treatment	No	Phase 2	No	Yes	No	No	1 (81)	1 (81)
NCT04365985[81]	Drug: Naltrexone|Drug: Ketamine|Other: Placebo	Other	treatment	No	Phase 2	Yes	Yes	Yes	Distinct from Race	1 (70)	1 (70)

© 2022 Xiao H et al. *JAMA Intern Med.*

NCT04368260[82]	Device: Control swab|Device: Prototype swab	Other	screening	Yes	Not Applicable	No	Yes	No	No	1 (40)	1 (40)
NCT04372186[83]	Drug: Placebo|Drug: Tocilizumab	Industry	treatment	No	Phase 3	Yes	Yes	Yes	Distinct from Race	51 (377)	32 (377)
NCT04374019[84]	Drug: Ivermectin|Drug: Camostat Mesilate|Dietary Supplement: Artemesia annua|Drug: Artesunate	Other	treatment	No	Phase 2	Yes	Yes	Yes	Distinct from Race	1 (13)	1 (13)
NCT04377620[85]	Drug: Placebo|Drug: Ruxolitinib	Industry	treatment	Yes	Phase 3	Yes	Yes	Yes	Distinct from Race	36 (211)	35 (211)
NCT04377711[86]	Drug: Ciclesonide|Drug: Placebo	Industry	treatment	Yes	Phase 3	Yes	Yes	Yes	Distinct from Race	1 (400)	1 (400)
NCT04380688[87]	Drug: Acalabrutinib	Industry	treatment	No	Phase 2	Yes	Yes	Yes	Distinct from Race	29 (62)	29 (62)
NCT04388826[88]	Drug: Veru111	Industry	treatment	No	Phase 2	Yes	Yes	Yes	Distinct from Race	5 (39)	5 (39)
NCT04399980[89]	Drug: Mavrilimumab|Drug: Placebos	Other	treatment	No	Phase 2	Yes	Yes	No	No	2 (40)	2 (40)
NCT04401293[90]	Drug: Enoxaparin|Drug: Prophylactic/Intermediate Dose Enoxaparin	Other	treatment	No	Phase 3	Yes	Yes	Yes	No	6 (253)	6 (253)
NCT04401579[91]	Other: Placebo|Drug: Remdesivir|Drug: Baricitinib	NIH	treatment	No	Phase 3	Yes	Yes	Yes	Distinct from Race	71 (1033)	53 (1033)
NCT04402970[92]	Drug: Dornase Alfa Inhalation Solution	Other	treatment	No	Phase 3	No	Yes	Yes	Not distinct from race	1 (30)	1 (30)
NCT04405570[93]	Drug: Molnupiravir 200 mg|Drug: Molnupiravir 400 mg|Drug: Molnupiravir 800 mg|Drug: Placebo (PBO)	Industry	treatment	No	Phase 2	Yes	Yes	Yes	Distinct from Race	10 (202)	10 (202)
NCT04409509[94]	Biological: Garadacimab, Factor XIIa Antagonist Monoclonal Antibody|Drug: Placebo	Industry	treatment	No	Phase 2	Yes	Yes	Yes	Distinct from Race	14 (124)	14 (124)
NCT04411628[95]	Drug: LY3819253|Drug: Placebo	Industry	treatment	No	Phase 1	Yes	Yes	Yes	Distinct from Race	11 (24)	11 (24)
NCT04411667[96]	Drug: Octagam	Other	treatment	No	Phase 4	Yes	Yes	No	Distinct from Race	2 (33)	2 (33)
NCT04411680[97]	Drug: Sargramostim|Drug: Standard of care	Industry	treatment	No	Phase 2	Yes	Yes	Yes	Distinct from Race	11 (123)	11 (123)
NCT04421404[98]	Biological: COVID19 Convalescent Plasma (CCP)|Biological: Placebo	Other	treatment	No	Phase 2	Yes	Yes	Yes	Distinct from Race	3 (34)	3 (34)
NCT04425252[99]	Drug: Brequinar|Other: Standard of Care	Industry	treatment	No	Phase 1|2	Yes	Yes	Yes	No	5 (23)	5 (23)
NCT04425538[100]	Drug: Infliximab	Other	treatment	No	Phase 2	No	Yes	Yes	Not distinct from race	1 (17)	1 (17)
NCT04425720[101]	Device: LifeSignals Biosensor 1AX*|Other: Standard of Care	Other	supportive care	No	Not Applicable	Yes	Yes	No	Distinct from Race	1 (280)	1 (280)
NCT04432272[102]	Biological: COVID19 convalescent plasma	Other	treatment	No	Phase 2	No	Yes	Yes	Distinct from Race	1 (54)	1 (54)
NCT04435184[103]	Drug: Crizanlizumab|Other: 0.9% saline	Other	treatment	No	Phase 2	Yes	Yes	Yes	No	1 (42)	1 (42)

© 2022 Xiao H et al. *JAMA Intern Med.*

NCT04456153[104]	Drug: Experimental Group|Drug: Placebo Group	Other	supportive care	No	Phase 2	Yes	Yes	Yes	Distinct from Race	1 (60)	1 (60)
NCT04460690[105]	Device: Rapid Onsite COVID19 Detection	Other	diagnosis	Yes	Not Applicable	No	No	No	No	1 (93)	1 (93)
NCT04492475[106]	Drug: Interferon beta1a|Other: Placebo|Drug: Remdesivir	NIH	treatment	No	Phase 3	Yes	Yes	Yes	Distinct from Race	64 (969)	55 (860)
NCT04494646[107]	Drug: Bardoxolone Methyl|Drug: Placebo	Other	treatment	No	Phase 2	Yes	Yes	Yes	Distinct from Race	8 (38)	8 (38)
NCT04498247[108]	Biological: V591|Other: Placebo	Industry	prevention	No	Phase 1|2	Yes	Yes	Yes	Distinct from Race	9 (263)	4 (263)
NCT04498273[109]	Drug: Apixaban 2.5 MG|Drug: Apixaban 5MG|Drug: Aspirin|Drug: Placebo	Other	treatment	No	Phase 3	Yes	Yes	Yes	No	71 (657)	71 (657)
NCT04501952[110]	Drug: RDV|Drug: Placebo to Match RDV	Industry	treatment	Yes	Phase 3	Yes	Yes	Yes	Distinct from Race	104 (562)	92 (531)
NCT04502472[111]	Biological: Convalescent plasma transfusion	Other	treatment	No	Phase 2|3	No	Yes	Yes	Distinct from Race	1 (109)	1 (109)
NCT04504032[112]	Drug: Rivaroxaban|Drug: Placebo	Other	treatment	No	Phase 2	Yes	Yes	Yes	Distinct from Race	20 (497)	17 (497)
NCT04524507[113]	Biological: Hightiter Convalescent COVID19 Plasma (CCP1)|Biological: Standardtiter Convalescent COVID19 plasma (CCP2)	Other	treatment	No	Phase 2	Yes	Yes	Yes	Distinct from Race	1 (56)	1 (56)
NCT04569786[114]	Biological: V590|Other: Placebo	Industry	prevention	No	Phase 1	Yes	Yes	Yes	Distinct from Race	7 (232)	7 (232)
NCT04579549[115]	Device: Saliva Assay	Other	diagnosis	Yes	Not Applicable	No	No	No	No	1 (464)	1 (464)
NCT04583592[116]	Drug: Camostat Mesilate|Drug: Placebo	Industry	treatment	No	Phase 2	Yes	Yes	Yes	Distinct from Race	21 (295)	21 (295)
NCT04610489[117]	Diagnostic Test: Quidel Sofia SARS Antigen FIA	Other	diagnosis	No	Not Applicable	No	No	No	No	1 (117)	1 (117)
NCT04685213[118]	Device: Electrical Stimulation|Device: Electrical Stimulation Sham	Other	supportive care	No	Phase 1	Yes	Yes	Yes	No	1 (19)	1 (19)
NCT04701658[119]	Drug: Bamlanivimab	Industry	treatment	Yes	Phase 2	No	Yes	Yes	Distinct from Race	1 (109)	1 (109)
NCT04706416[120]	Dietary Supplement: Nacetyl glucosamine (NAG)|Other: Control	Industry	treatment	No	Phase 1	No	Yes	No	Distinct from Race	1 (148)	1 (148)
NCT04871815[121]	Drug: sodium pyruvate nasal spray	Industry	treatment	No	Phase 2|3	No	Yes	No	No	1 (22)	1 (22)
NCT05087524[122]	Diagnostic Test: BinaxNOW Ag Card	Other	diagnosis	Yes	Not Applicable	No	No	No	No	1 (65)	1 (65)

© 2022 Xiao H et al. *JAMA Intern Med.*

Table 2A. Sex and race/ethnicity representation in COVID-19 clinical trials

Demographic Domain		Estimated proportion of participants (95 % CI)	Effect of Moderator		Proportion of population (%)		Proportion of cumulative COVID-19 incidence as of April 2021*	
Category	Category Level		OR (95% CI)	p-value	Unadjusted (p-value)	Adjusted (p-value)	Unadjusted (p-value)	Adjusted (p-value)
**Female**								
Overall (n=109)		45.3 (43.2, 47.4)			50.5 (<.001)		52.4 (<.001)	
Purpose	Prevention (n=14)	48.9 (44.5, 53.4)	1.19 (0.97, 1.46)	.10	(.48)		(.13)	
	Treatment (n=95)	44.6 (42.3, 47.0)	Ref	Ref	(<.001)		(<.001)	
Phase	Phase 1 or 2 (n=59)	45.1 (41.9, 48.3)	Ref	Ref	(<.001)		(<.001)	
	Phase 3 or 4 (n=36)	46.1 (43.2, 49.1)	1.04 (0.87, 1.25)	.06	(<.001)		(<.001)	
Primary Funder	Government (n=6)	43.0 (36.1, 50.2)	0.88 (0.65, 1.21)	.44	(<.001)		(<.001)	
	Industry (n=40)	46.0 (43.2, 48.9)	Ref	Ref	(<.001)		(<.001)	
	Other (n=61)	44.6 (40.3, 47.9)	0.94 (0.79, 1.13)	.52	(<.001)		(<.001)	
**Race: White**								
Overall (n=78)		73.9 (69.7, 77.7)			76.3 (.22)	75.0 (.56)	77.9 (.04)	75.9 (.46)
Purpose	Prevention (n=12)	85.7 (80.3, 89.9)	2.49 (1.60, 3.88)	<.001	(<.002)	75.1 (<.001)	(.007)	76.1 (.001)
	Treatment (n=66)	70.7 (66.1, 74.9)	Ref	Ref	(.008)	74.8 (.05)	(.001)	75.0 (.04)
Phase	Phase 1 or 2 (n=48)	75.3 (67.4, 77.7)	Ref	Ref	(.76)	73.2 (.49)	(.36)	73.6 (.56)
	Phase 3 or 4 (n=26)	72.9 (67.4, 77.7)	0.88 (0.59, 1.31)	.54	(.18)	75.6 (.29)	(.04)	76.6 (.14)
Primary Funder	Government (n=5)	74.0 (58.1, 86.4)	0.77 (0.36, 1.65)	.51	(.74)	73.6 (.95)	(.57)	73.5 (.94)
	Industry (n=38)	78.6 (74.0, 82.6)		Ref	(.31)	75.1 (.13)	(.73)	76.0 (.24)
	Other (n=35)	66.9 (59.3, 73.7)	0.55 (0.36, 0.82)	.005	(.005)	75.8 (.009)	(<.001)	75.9 (.008)
**Race: Black**								
Overall (n=78)		14.3 (11.8, 17.2)			13.4 (.53)	14.2 (.98)	14.1 (.91)	15.3 (.46)
Purpose	Prevention (n=12)	7.2 (4.7, 10.9)	0.39 (0.23, 0.66)	<.001	(.003)	14.3 (.001)	(.001)	15.3 (<.001)
	Treatment (n=66)	16.5 (13.6, 19.9)	Ref	Ref	(.07)	13.8 (.07)	(.11)	15.3 (.44)
Phase	Phase 1 or 2 (n=48)	14.1 (10.6, 18.5)	Ref	Ref	(.73)	12.4 (.38)	(.99)	13.6 (.81)
	Phase 3 or 4 (n=26)	13.1 (10.2, 16.6)	0.92 (0.60, 1.40)	.70	(.54)	14.7 (.33)	(.54)	15.8 (.12)
Primary Funder	Government (n=5)	20.2 (17.0, 23.7)	1.83 (1.29, 2.62)	<.001	(<.001)	14.4 (<.001)	(<.001)	16.1 (.009)
	Industry (n=38)	12.1 (9.4, 15.4)	Ref	Ref	(.42)	14.2 (.19)	(.22)	15.3 (.05)
	Other (n=35)	17.1 (12.4, 23.1)	1.50 (0.94, 2.40)	.09	(.14)	14.2 (.25)	(.24)	15.6 (.58)
**Race: Asian**								
Overall (n=78)		4.4 (3.6, 5.6)			5.9 (.06)	5.5 (.05)	3.7 (.13)	3.8 (.16)
Purpose	Prevention (n=12)	3.8 (2.9, 5.1)	0.82 (0.55, 1.23)	.35	(.003)	5.3 (.03)	(.85)	3.5 (.54)
	Treatment (n=66)	4.6 (3.6, 6.0)	Ref	Ref	(.07)	7.0 (.002)	(.11)	5.3 (.31)
Phase	Phase 1 or 2 (n=48)	3.7 (2.7, 5.0)	Ref	Ref	(.003)	5.8 (.004)	(.96)	4.3 (.32)
	Phase 3 or 4 (n=26)	5.9 (4.3, 8.1)	1.65 (1.03, 2.64)	.04	(.98)	5.5 (.63)	(.005)	3.6 (.003)
Primary Funder	Government (n=5)	11.8 (9.3, 14.9)	3.26 (2.28, 4.66)	<.001	(<.001)	7.5 (<.001)	(<.001)	5.5 (<.001)
	Industry (n=38)	4.0 (3.2, 5.0)	Ref	Ref	(<.001)	5.5 (.004)	(.63)	3.7 (.60)
	Other (n=35)	4.6 (2.9, 7.0)	1.16 (0.69, 1.94)	.58	(.24)	6.2 (.16)	(.38)	4.6 (.98)
**Race: Native Hawaiian / Pacific Islander**								
Overall (n=78)		0.61 (0.47, 0.87)			0.20 (<.001)	0.24 (<.001)	0.21 (<.001)	0.19 (<.001)
Purpose	Prevention (n=12)	0.23 (0.20, 0.25)	0.26 (0.20, 0.32)	<.001	(.03)	0.24 (.33)	(.18)	0.19 (.002)
	Treatment (n=66)	0.89 (0.71, 1.11)	Ref	Ref	(<.001)	0.24 (<.001)	(<.001)	0.24 (<.001)
Phase	Phase 1 or 2 (n=48)	1.06 (0.74, 1.50)	Ref	Ref	(<.001)	0.28 (<.001)	(<.001)	0.16 (<.001)
	Phase 3 or 4 (n=26)	0.47 (0.30, 0.73)	0.44 (0.20, 0.37)	<.001	(<.001)	0.23 (.003)	(<.001)	0.20 (<.001)
Primary Funder	Government (n=5)	1.02 (0.60, 1.73)	2.09 (1.05, 4.09)	.03	(<.001)	0.31 (<.001)	(<.001)	0.33 (<.001)
	Industry (n=38)	0.49 (0.33, 0.74)	Ref	Ref	(<.001)	0.24 (<.001)	(<.001)	0.19 (<.001)
	Other (n=35)	0.75 (0.53, 1.07)	1.53 (0.90. 2.61)	.12	(<.001)	0.18 (<.001)	(<.001)	0.15 (<.001)
**Race: American Indian / Alaska Native**								
Overall (n=78)		1.3 (1.0, 1.8)			1.3 (.88)	1.3 (.92)	1.1 (.29)	1.0 (.06)
Purpose	Prevention (n=12)	1.1 (0.6, 2.0)	0.78 (0.40, 1.59)	.49	(.58)	1.3 (.53)	(.94)	1.0 (.79)
	Treatment (n=66)	1.4 (1.0, 2.0)	Ref	Ref	(.68)	1.2 (.39)	(.23)	0.9 (.01)
Phase	Phase 1 or 2 (n=48)	1.6 (1.1, 2.4)	Ref	Ref	(.24)	1.3 (.25)	(.06)	1.2 (.09)
	Phase 3 or 4 (n=26)	1.1 (0.7 1.8)	0.68 (0.37, 1.29)	.23	(.56)	1.3 (.52)	(.98)	1.0 (.53)
Primary Funder	Government (n=5)	1.2 (0.8, 1.7)	0.89 (0.51, 1.52)	.66	(.57)	1.2 (.87)	(.84)	0.8 (.02)
	Industry (n=38)	1.3 (0.9, 2.0)	Ref	Ref	(.93)	1.3 (.98)	(.45)	1.0 (.21)
	Other (n=35)	1.3 (0.8, 2.3)	1.01 (0.50, 1.52)	.99	(.94)	1.1 (.39)	(.56)	0.8 (.04)
**Ethnicity: Hispanic**								
Overall (n=70)		34.1 (27.8, 41.1)			19.5 (<.001)	18.9 (<.001)	17.7 (<.001)	21.6 (<.001)
Purpose	Prevention (n=11)	23.0 (16.7, 30.7)	0.52 (0.31, 0.87)	.01	(.30)	18.7 (.22)	(.07)	21.4 (.65)
	Treatment (n=59)	36.6 (29.1, 44.9)	Ref	Ref	(<.001)	21.4 (<.001)	(<.001)	24.7 (<.001)
Phase	Phase 1 or 2 (n=42)	31.2 (22.2, 41.9)	Ref	Ref	(.008)	18.2 (.003)	(.002)	20.3 (.01)
	Phase 3 or 4 (n=24)	33.9 (27.0, 42.5)	1.13 (0.63, 1.99)	.67	(<.001)	19.5 (<.001)	(<.001)	22.4 (<.001)
Primary Funder	Government (n=5)	30.0 (18.6, 44.5)	0.73 (0.36, 1.52)	.38	(.07)	20.4 (.11)	(.03)	24.3 (.37)
	Industry (n=35)	37.1 (29.6, 45.2)	Ref	Ref	(<.001)	19.1 (<.001)	(<.001)	21.9 (<.001)
	Other (n=30)	30.3 (19.3, 44.2)	0.74 (0.37, 1.48)	.39	(.06)	17.0 (.01)	(.02)	19.1 (.04)

* The comparison to cumulative COVID-19 incidence as of February 2022 was presented in eTable 2d

© 2022 Xiao H et al. *JAMA Intern Med.*

eTable 2B. Sex and race/ethnicity representation in COVID-19 prevention clinical trials

Demographic Domain		Estimated proportion of participants (95 % CI)	Effect of Moderator		Proportion of population (%)		Proportion of cumulative COVID-19 incidence as of April 2021*	
Category	Category Level		OR (95% CI)	p-value	Unadjusted (p-value)	Adjusted (p-value)	Unadjusted (p-value)	Adjusted (p-value)
**Female**								
Overall (n=14)		48.9 (44.5, 53.4)			50.5 (.48)		52.4 (.13)	
Phase	Phase 1 or 2 (n=5)	49.3 (47.2, 51.3)	Ref	Ref	(.57)		(.31)	
	Phase 3 or 4 (n=8)	48.1 (39.0, 56.4)	0.95 (0.68, 1.35)	0.79	(.23)		(.002)	
Primary Funder	Government (n=1)	51.1 (36.8, 65.2)	1.05 (0.58, 1.74)	0.87	(.93)		(.86)	
	Industry (n=11)	49.8 (45.2, 54.5)	Ref	Ref	(.78)		(.28)	
	Other (n=2)	39.9 (23.6, 58.7)	1.49 (0.69, 3.25)	0.31	(.27)		(.19)	
**Race: White**								
Overall (n=12)		85.7 (80.3, 89.9)			76.3 (<.002)	75.1 (<.001)	77.9 (.007)	76.1 (.001)
Phase	Phase 1 or 2 (n=7)	88.1 (78.9, 93.6)	Ref	Ref	(.02)	62.8 (<.001)	(.03)	63.1 (<.001)
	Phase 3 or 4 (n=5)	82.6 (79.5, 85.4)	0.64 (0.32, 1.31)	0.23	(<.001)	75.8 (<.001)	(.005)	77.0 (<.001)
Primary Funder	Government (n=1)	90.0 (78.2, 96.6)	1.70 (0.56, 5.15)	0.34	(.04)	65.7 (.002)	(.04)	63.1 ()<.001
	Industry (n=11)	81.1 (80.8, 81.3)	Ref	Ref	(.004)	75.1 (.002)	(.02)	76.1 (.004)
	Other (n=0)							
**Race: Black**								
Overall (n=12)		7.2 (4.7, 10.9)			13.4 (.003)	14.3 (.001)	14.1 (.001)	15.3 (<.001)
Phase	Phase 1 or 2 (n=7)	8.2 (5.4, 12.5)	Ref	Ref	(.03)	10.6 (.15)	(.02)	11.6 (.08)
	Phase 3 or 4 (n=5)	6.3 (3.0, 12.6)	0.75 (0.30, 1.82)	0.53	(.02)	14.9 (.004)	(.01)	15.9 (.002)
Primary Funder	Government (n=1)	4.6 (1.1, 16.4)	0.60 (0.13, 2.27)	0.50	(.10)	22.7 (.01)	(.09)	26.2 (.01)
	Industry (n=11)	7.4 (4.7, 11.4)	Ref	Ref	(.006)	14.3 (.002)	(.003)	15.3 (<.001)
	Other (n=0)							
**Race: Asian**								
Overall (n=12)		3.8 (2.9, 5.1)			5.9 (.003)	5.3 (.03)	3.7 (.85)	3.5 (.54)
Phase	Phase 1 or 2 (n=7)	2.9 (1.5, 5.4)	Ref	Ref	(.03)	5.0 (.07)	(.45)	3.7 (.45)
	Phase 3 or 4 (n=5)	4.5 (3.6, 5.5)	1.56 (0.78, 3.10)	0.21	(.007)	5.1 (.09)	(.08)	3.3 (.005)
Primary Funder	Government (n=1)	2.3 (3.2, 14.5)	0.58 (0.77, 4.26)	0.59	(.37)	6.9 (.25)	(.62)	4.2 (.53)
	Industry (n=11)	3.9 (2.9, 5.2)	Ref	Ref	(.004)	5.3 (.03)	(.75)	3.5 (.49)
	Other (n=0)							
**Race: Native Hawaiian / Pacific Islander**								
Overall (n=12)		0.23 (0.20, 0.25)			0.20 (.03)	0.24 (.33)	0.21 (.18)	0.19 (.002)
Phase	Phase 1 or 2 (n=7)	0.26 (0.21, 0.32)	Ref	Ref	(.02)	0.25 (.78)	(.06)	0.14 (.005)
	Phase 3 or 4 (n=5)	0.22 (0.19, 0.25)	0.84 (0.65, 1.08)	0.16	(.26)	0.22 (.78)	(.68)	0.19 (.06)
Primary Funder	Government (n=1)	1.11 (0.07, 15.43)	5.00 (3.07, 79.79)	0.26	(.22)	0.33 (.39)	(.24)	0.49 (.56)
	Industry (n=11)	0.23 (0.02, 0.25)	Ref	Ref	(.03)	0.23 (.78)	(.19)	0.18 (<.001)
	Other (n=0)							
**Race: American Indian / Alaska Native**								
Overall (n=12)		1.1 (0.6, 2.0)			1.3 (.58)	1.3 (.53)	1.1 (.94)	1.0 (.79)
Phase	Phase 1 or 2 (n=7)	1.2 (0.5, 2.5)	Ref	Ref	(.77)	1.1(.92)	(.90)	1.0 (.65)
	Phase 3 or 4 (n=5)	1.1 (0.4, 2.7)	0.94 (0.27, 3.29)	0.91	(.69)	1.3 (.64)	(.96)	1.0 (.85)
Primary Funder	Government (n=1)	1.1 (0.6, 1.9)	2.17 (0.27, 17.2)	0.46	(.57)	1.0 (.41)	(.47)	0.4 (.66)
	Industry (n=11)	2.3 (0.3, 14.5)	Ref	Ref	(.51)	1.3 (.53)	1.1 (.94)	1.0 (.79)
	Other (n=0)							
**Ethnicity: Hispanic**								
Overall (n=11)		23.0 (16.7, 30.7)			19.5 (.30)	18.7 (.22)	17.7 (.07)	21.4 (.65)
Phase	Phase 1 or 2 (n=6)	23.0 (13.6, 36.0)	Ref	Ref	(.52)	17.7 (.31)	(.31)	19.6 (.54)
	Phase 3 or 4 (n=5)	23.0 (15.0, 33.3)	1.01 (0.44, 2.78)	0.99	(.45)	19.1 (39)	(.23)	22.0 (.85)
Primary Funder	Government (n=1)	13.3 (6.1, 26.7)	0.50 (0.19, 1.31)	0.15	(.30)	10.1 (.47)	(.44)	13.4 (.99)
	Industry (n=10)	23.8 (17.1, 32.1)	Ref	Ref	(.23)	18.8 (.15)	(.08)	21.4 (.51)
	Other (n=0)							

© 2022 Xiao H et al. *JAMA Intern Med.*

eTable 2C. Sex and race/ethnicity representation in COVID-19 treatment clinical trials

Demographic Domain		Estimated proportion of participants (95 % CI)	Effect of Moderator		Proportion of population (%)		Proportion of cumulative COVID-19 incidence as of April 2021*	
Category	Category Level		OR (95% CI)	p-value	Unadjusted (p-value)	Adjusted (p-value)	Unadjusted (p-value)	Adjusted (p-value)
**Female**								
Overall (n=95)		44.6 (42.3, 47.0)			50.5 (<.001)		52.4 (<.001)	
Phase	Phase 1 or 2 (n=51)	44.5 (41.0, 48.0)	Ref	Ref	(<.001)		(<.001)	
	Phase 3 or 4 (n=31)	45.5 (42.1, 48.9)	1.04 (0.86, 1.27)	0.69	(.004)		(<.001)	
Primary Funder	Government (n=5)	40.9 (35.0, 47.1)	0.87 (0.65, 1.16)	0.36	(.002)		(<.001)	
	Industry (n=29)	44.2 (40.1, 47.6)	Ref	Ref	(<.001)		(<.001)	
	Other (n=59)	44.7 (41.4, 48.2)	1.02 (0.84, 1.24)	0.82	(.001)		(<.001)	
**Race: White**								
Overall (n=66)		70.7 (66.1, 74.9)			76.3 (.008)	74.8 (.05)	77.9 (.001)	75.0 (.04)
Phase	Phase 1 or 2 (n=41)	71.9 (65.1, 77.8)	Ref	Ref	(.15)	76.0 (.18)	(.04)	76.4 (.14)
	Phase 3 or 4 (n=21)	70.0 (63.5, 75.7)	0.91 (0.60, 1.40)	0.67	(.03)	74.7 (.11)	(.005)	74.8 (.10)
Primary Funder	Government (n=4)	64.8 (59.8, 69.5)	0.61 (0.43, 0.88)	.009	(<.001)	73.7 (<.001)	(<.001)	73.7 (<.001)
	Industry (n=27)	74.9 (69.1, 80.0)	Ref	Ref	(.62)	74.8 (.96)	(.27)	74.9 (.99)
	Other (n=35)	66.9 (59.3, 73.7)	0.67 (0.43, 1.04)	0.08	(.006)	75.6 (.01)	(.001)	75.7 (.01)
**Race: Black**								
Overall (n=66)		16.5 (13.6, 19.9)	Ref	Ref	13.4 (.07)	13.8 (.07)	14.1 (.11)	15.3 (.44)
Phase	Phase 1 or 2 (n=41)	16.7 (12.6, 21.7)	Ref	Ref	(.12)	13.8 (.18)	(.23)	15.0 (.45)
	Phase 3 or 4 (n=21)	14.6 (11.1, 19.0)	0.86 (0.55, 1.35)	0.45	(.52)	13.7 (.73)	(.78)	15.4 (.71)
Primary Funder	Government (n=4)	20.8 (17.9, 24.0)	1.47 (1.03, 2.09)	0.03	(<.001)	14.3 (<.001)	(<.001)	15.9 (<.001)
	Industry (n=27)	15.1 (11.7, 19.4)	Ref	Ref	(.36)	13.6 (.49)	(.60)	15.1 (.99)
	Other (n=35)	17.1 (12.4, 23.1)	1.15 (0.71, 1.86)	0.55	(.14)	14.1 (.24)	(.24)	15.6 (.57)
**Race: Asian**								
Overall (n=66)		4.6 (3.6, 6.0)	Ref	Ref	5.9 (.07)	7.0 (.002)	3.7 (.11)	5.3 (.31)
Phase	Phase 1 or 2 (n=41)	3.9 (2.7, 5.6)	Ref	Ref	(.02)	6.0 (.02)	(.76)	4.2 (.69)
	Phase 3 or 4 (n=21)	6.4 (4.3, 9.3)	1.66 (0.95, 2.90)	0.07	(.71)	7.4 (.44)	(.008)	5.6 (.53)
Primary Funder	Government (n=4)	12.2 (9.7, 15.2)	3.31 (2.19, 5.00)	<.001	(<.001)	7.5 (<.001)	(<.001)	5.5 (<.001)
	Industry (n=27)	4.0 (2.9, 5.5)	Ref	Ref	(.01)	7.2 (<.001)	(.62)	5.5 (.04)
	Other (n=35)	4.6 (2.9, 7.0)	1.14 (0.65, 1.54)	0.64	(.24)	6.3 (.14)	(.36)	4.7 (.88)
**Race: Native Hawaiian / Pacific Islander**								
Overall (n=66)		0.89 (0.71, 1.11)			0.20 (<.001)	0.24 (<.001)	0.21 (<.001)	0.24 (<.001)
Phase	Phase 1 or 2 (n=41)	1.31 (0.90, 1.90)	Ref	Ref	(<.001)	0.17 (<.001)	(<.001)	0.16 (<.001)
	Phase 3 or 4 (n=21)	0.67 (0.46, 0.97)	0.51 (0.30, 0.87)	0.01	(<.001)	0.26 (<.001)	(<.001)	0.27 (<.001)
Primary Funder	Government (n=4)	1.01 (0.57, 1.78)	1.13 (0.55, 2.28)	0.73	(<.001)	0.31 (<.001)	(<.001)	0.33 (.001)
	Industry (n=27)	0.89 (0.59, 1.34)	Ref	Ref	(<.001)	0.25 (<.001)	(<.001)	0.26 (<.001)
	Other (n=35)	0.75 (0.53, 1.07)	0.84 (0.49, 1.44)	0.54	(<.001)	0.19 (<.001)	(<.001)	0.16 (<.001)
**Race: American Indian / Alaska Native**								
Overall (n=66)		1.4 (1.0, 2.0)			1.3 (.88)	1.2 (.39)	1.1 (.29)	0.9 (.01)
Phase	Phase 1 or 2 (n=41)	1.8 (1.2, 2.8)	Ref	Ref	(.14)	1.5 (.38)	(.03)	1.6(.58)
	Phase 3 or 4 (n=21)	1.1 (0.6, 2.0)	0.62 (0.29, 1.31)	0.20	(.63)	1.2 (.89)	(.94)	0.8 (.29)
Primary Funder	Government (n=4)	1.1 (0.8, 1.7)	0.76 (0.39, 1.45)	0.41	(.50)	1.2 (.87)	(.83)	0.8 (.03)
	Industry (n=27)	1.5 (0.9, 2.5)	Ref	Ref	(.60)	1.3 (.52)	(.25)	1.0 (.14)
	Other (n=35)	1.3 (0.8, 2.3)	0.88 (0.41, 1.89)	0.75	(.93)	1.1 (.45)	(.49)	0.8 (.07)
**Ethnicity: Hispanic**								
Overall (n=59)		36.6 (29.1, 44.9)			19.5 (<.001)	21.4 (<.001)	17.7 (<.001)	24.7 (<.001)
Phase	Phase 1 or 2 (n=36)	32.7 (29.1, 46.2)	Ref	Ref	(.01)	23.0 (.03)	(.003)	27.0 (.32)
	Phase 3 or 4 (n=19)	37.3 (29.1, 46.2)	1.23 (0.64, 2.38)	0.55	(<.001)	21.2 (<.001)	(<.001)	23.5 (<.001)
Primary Funder	Government (n=4)	34.6 (22.8, 48.6)	0.68 (0.33, 1.39)	0.30	(.001)	21.0 (.02)	(.003)	24.4 (.10)
	Industry (n=25)	43.5 (33.8, 53.6)	Ref	Ref	(<.001)	22.6 (<.001)	(<.001)	26.4 (<.001)
	Other (n=30)	30.3 (19.3, 44.2)	0.56 (0.27, 1.16)	0.12	(<.001)	17.3 (<.001)	(<.001)	19.1 (<.001)

© 2022 Xiao H et al. *JAMA Intern Med.*

eTable 2D. Sex and race/ethnicity representation in COVID-19 clinical trials
(COVID-19 incidence as of February 2022)

Demographic Domain		Estimated proportion of participants	Effect of Moderator		Proportion of population (%)		Proportion of cumulative COVID19 incidence as of Feb 2022	
Category	Category Level	(95 % CI)	OR (95% CI)	p-value	Unadjusted (p-value)	Adjusted (p-value)	Unadjusted (p-value)	Adjusted (p-value)
**Female**								
Overall(n=109))(n=109)		45.3 (43.2, 47.4)			50.5 (<.001)		53.1 (<.001)	
Purpose	Prevention (n=14)	48.9 (44.5, 53.4)	1.19 (0.97, 1.46)	0.1	(.48)		(.06)	
	Treatment (n=95)	44.6 (42.3, 47.0)	Ref	Ref	(<.001)		(<.001)	
Phase	Phase 1 or 2 (n=59)	45.1 (41.9, 48.3)	Ref	Ref	(<.001)		(<.001)	
	Phase 3 or 4 (n=36)	46.1 (43.2, 49.1)	1.04 (0.87, 1.25)	0.06	(<.001)		(<.001)	
Primary Funder	Government (n=6)	43.0 (36.1, 50.2)	0.88 (0.65, 1.21)	0.44	(<.001)		(<.001)	
	Industry (n=40)	46.0 (43.2, 48.9)	Ref	Ref	(<.001)		(<.001)	
	Other (n=61)	44.6 (40.3, 47.9)	0.94 (0.79, 1.13)	0.52	(<.001)		(<.001)	
**Race: White**								
Overall (n=78)		73.9 (69.7, 77.7)			76.3 (.22)	75.0 (.56)	76.0 (.29)	73.6 (.89)
Purpose	Prevention (n=12)	85.7 (80.3, 89.9)	2.49 (1.60, 3.88)	<.001	(<.002)	75.1 (<.001)	(.001)	73.7 (<.001)
	Treatment (n=66)	70.7 (66.1, 74.9)	Ref	Ref	(.008)	75.0 (.04)	(.01)	72.9 (.31)
Phase	Phase 1 or 2 (n=48)	75.3 (67.4, 77.7)	Ref	Ref	(.76)	73.2 (.49)	(.79)	71.8 (.25)
	Phase 3 or 4 (n=26)	72.9 (67.4, 77.7)	0.88 (0.59, 1.31)	0.54	(.18)	75.6 (.29)	(0.21)	74.1 (.64)
Primary Funder	Government (n=5)	74.0 (58.1, 86.4)	0.77 (0.36, 1.65)	0.51	(.74)	73.6 (.95)	(.77)	71.4 (.72)
	Industry (n=38)	78.6 (74.0, 82.6)		Ref	(.31)	75.1 (.13)	(.26)	73.6 (.03)
	Other (n=35)	66.9 (59.3, 73.7)	0.55 (0.36, 0.82)	0.005	(.005)	75.8 (.009)	(.007)	74.4(.03)
**Race: Black**								
Overall (n=78)		14.3 (11.8, 17.2)			13.4 (.53)	14.2 (.98)	15.0 (.59)	16.1 (.21)
Purpose	Prevention (n=12)	7.2 (4.7, 10.9)	0.39 (0.23, 0.66)	<.001	(.003)	14.3 (.001)	(<.001)	16.1 (<.001)
	Treatment (n=66)	16.5 (13.6, 19.9)	Ref	Ref	(.07)	13.8 (.07)	(.38)	16.0 (.76)
Phase	Phase 1 or 2 (n=48)	14.1 (10.6, 18.5)	Ref	Ref	(.73)	12.4 (.38)	(.65)	14.1 (.98)
	Phase 3 or 4 (n=26)	13.1 (10.2, 16.6)	0.92 (0.60, 1.40)	0.7	(.54)	14.7 (.33)	(.27)	16.65 (.04)
Primary Funder	Government (n=5)	20.2 (17.0, 23.7)	1.83 (1.29, 2.62)	<.001	(<.001)	14.4 (<.001)	(<.001)	16.7 (.03)
	Industry (n=38)	12.1 (9.4, 15.4)	Ref	Ref	(.42)	14.2 (.19)	(.08)	16.1 (.02)
	Other (n=35)	17.1 (12.4, 23.1)	1.50 (0.94, 2.40)	0.09	(.14)	14.2 (.25)	(.42)	16.1 (.71)
**Race: Asian**								
Overall (n=78)		4.4 (3.6, 5.6)			5.9 (.06)	5.5 (.05)	4.3 (.72)	4.3 (.76)
Purpose	Prevention (n=12)	3.8 (2.9, 5.1)	0.82 (0.55, 1.23)	0.35	(.003)	5.3 (.03)	(.48)	4.0 (.76)
	Treatment (n=66)	4.6 (3.6, 6.0)	Ref	Ref	(.07)	7.0 (.002)	(.53)	5.9 (.07)
Phase	Phase 1 or 2 (n=48)	3.7 (2.7, 5.0)	Ref	Ref	(.003)	5.8 (.004)	(.35)	4.8 (.09)
	Phase 3 or 4 (n=26)	5.9 (4.3, 8.1)	1.65 (1.03, 2.64)	0.04	(.98)	5.5 (.63)	(.04)	4.2 (.03)
Primary Funder	Government (n=5)	11.8 (9.3, 14.9)	3.26 (2.28, 4.66)	<.001	(<.001)	7.5 (<.001)	(<.001)	6.0 (<.001)
	Industry (n=38)	4.0 (3.2, 5.0)	Ref	Ref	(<.001)	5.5 (.004)	(.53)	4.2 (.60)
	Other (n=35)	4.6 (2.9, 7.0)	1.16 (0.69, 1.94)	0.58	(.24)	6.2 (.16)	(.77)	5.1 (.63)
**Race: Native Hawaiian / Pacific Islander**								
Overall (n=78)		0.61 (0.47, 0.87)			0.20 (<.001)	0.24 (<.001)	0.25 (<.001)	0.21 (<.001)
Purpose	Prevention (n=12)	0.23 (0.20, 0.25)	0.26 (0.20, 0.32)	<.001	(.03)	0.24 (.33)	(.09)	0.20 (.03)
	Treatment (n=66)	0.89 (0.71, 1.11)	Ref	Ref	(<.001)	0.24 (<.001)	(<.001)	0.27 (<.001)
Phase	Phase 1 or 2 (n=48)	1.06 (0.74, 1.50)	Ref	Ref	(<.001)	0.28 (<.001)	(<.001)	0.18 (<.001)
	Phase 3 or 4 (n=26)	0.47 (0.30, 0.73)	0.44 (0.20, 0.37)	<.001	(<.001)	0.23 (.003)	(.01)	0.22 (.002)
Primary Funder	Government (n=5)	1.02 (0.60, 1.73)	2.09 (1.05, 4.09)	0.03	(<.001)	0.31 (<.001)	(<.001)	0.35 (<.001)
	Industry (n=38)	0.49 (0.33, 0.74)	Ref	Ref	(<.001)	0.24 (<.001)	(<.001)	0.21 (<.001)
	Other (n=35)	0.75 (0.53, 1.07)	1.53 (0.90. 2.61)	0.12	(<.001)	0.18 (<.001)	(<.001)	0.16 (<.001)
**Race: American Indian / Alaska Native**								
Overall (n=78)		1.3 (1.0, 1.8)			1.3 (.88)	1.3 (.92)	1.1 (.31)	1.0 (.07)
Purpose	Prevention (n=12)	1.1 (0.6, 2.0)	0.78 (0.40, 1.59)	0.49	(.58)	1.3 (.53)	(.91)	1.1 (.92)
	Treatment (n=66)	1.4 (1.0, 2.0)	Ref	Ref	(.68)	1.2 (.39)	(.25)	0.9 (.01)
Phase	Phase 1 or 2 (n=48)	1.6 (1.1, 2.4)	Ref	Ref	(.24)	1.3 (.25)	(.06)	1.2 (.09)
	Phase 3 or 4 (n=26)	1.1 (0.7 1.8)	0.68 (0.37, 1.29)	0.23	(.56)	1.3 (.52)	(.95)	1.0 (.73)
Primary Funder	Government (n=5)	1.2 (0.8, 1.7)	0.89 (0.51, 1.52)	0.66	(.57)	1.2 (.87)	(.88)	0.8 (.07)
	Industry (n=38)	1.3 (0.9, 2.0)	Ref	Ref	(.93)	1.3 (.98)	(.47)	1.0 (.21)
	Other (n=35)	1.3 (0.8, 2.3)	1.01 (0.50, 1.52)	0.99	(.94)	1.1 (.39)	(0.58)	0.8 (.04)
**Ethnicity: Hispanic / Latino**								
Overall (n=70)		34.1 (27.8, 41.1)			19.5 (<.001)	18.9 (<.001)	15.4 (<.001)	19.2 (<.001)
Purpose	Prevention (n=11)	23.0 (16.7, 30.7)	0.52 (0.31, 0.87)	0.01	(.30)	18.7 (.22)	(.01)	19.0 (.24)
	Treatment (n=59)	36.6 (29.1, 44.9)	Ref	Ref	(<.001)	21.4 (<.001)	(<.001)	21.9 (<.001)
Phase	Phase 1 or 2 (n=42)	31.2 (22.2, 41.9)	Ref	Ref	(.008)	18.2 (.003)	(<.001)	18.2 (.003)
	Phase 3 or 4 (n=24)	33.9 (27.0, 42.5)	1.13 (0.63, 1.99)	0.67	(<.001)	19.5 (<.001)	(<.001)	19.9 (<.001)
Primary Funder	Government (n=5)	30.0 (18.6, 44.5)	0.73 (0.36, 1.52)	0.38	(.07)	20.4 (.11)	(.008)	21.2 (.15)
	Industry (n=35)	37.1 (29.6, 45.2)	Ref	Ref	(<.001)	19.1 (<.001)	(<.001)	19.5 (<.001)
	Other (n=30)	30.3 (19.3, 44.2)	0.74 (0.37, 1.48)	0.39	(.15)	17.0 (.01)	(.004)	16.9 (.01)

© 2022 Xiao H et al. *JAMA Intern Med.*

eTable 3. Race/ethnicity representation in COVID-19 clinical trials: results from analyses stratified by locations of clinical trials (COVID-19 incidence as of April 2021)

	Estimated proportion of participants (95 % CI)	Effect of Moderator			Proportion of population (%)			Proportion of cumulative COVID-19 incidence as of April 2021*	
		OR	p-value		Unadjusted (p-value)	Adjusted (p-value)		Unadjusted (p-value)	Adjusted (p-value)
White									
High white[1] (n=7)	74.3 (52.2, 88.4)	1.19 (0.41, 3.40)	0.75		92.5 (.003)	79.2 (0.58)		81.3 (0.41)	80.7 (0.46)
Moderate white[1] (n=42)	75.7 (71.1, 79.8)	1.29 (0.79, 2.10)	0.31		76.3 (0.79)	75.1 (0.78)		77.9 (0.42)	75.7 (0.98)
Low white[1] (n=29)	70.8 (61.5, 78.7)	Ref	Ref		64.8 (0.20)	68.7 (0.64)		62.7 (0.09)	64.0 (0.15)
Black									
High black[2] (n=12)	18.7 (8.8, 35.3)	1.32 (0.48, 3.55)	0.59		22.8 (0.57)	19.3 (0.93)		24.4 (0.44)	22.5 (0.60)
Moderate black[2] (n=48)	13.1 (10.6, 16.1)	0.86 (0.50, 1.50)	0.61		13.4 (0.82)	13.9 (0.57)		14.1 (0.48)	15.2 (0.15)
Low black[2] (n=18)	14.9 (9.5, 22.5)	Ref	Ref		9.2 (0.03)	10.1 (0.09)		9.3 (0.04)	10.7 (0.15)
Asian									
High Asian[3] (n=9)	7.7 (3.3, 16.7)	3.21 (1.14, 8.86)	0.03		12.4 (0.24)	9.2 (0.67)		10.4 (0.46)	9.0 (0.70)
Moderate Asian[3] (n=40)	5.0 (4.0, 6.2)	2.04 (1.15, 3.64)	0.01		5.9 (0.15)	6.7 (.009)		3.7 (.006)	4.8 (0.68)
Low Asian[3] (n=29)	2.5 (1.5, 4.2)	Ref	Ref		3.6 (0.16)	3.7 (0.15)		1.7 (0.12)	1.7 (0.11)
Native Hawaiian / Pacific Islander									
High Native Hawaiian[4] (n=3)	2.23 (0.45, 10.35)	2.60 (0.51, 13.49)	0.26		0.76 (0.19)	0.50 (0.07)		0.93 (0.28)	0.92 (0.28)
Moderate Native Hawaiian[4]	0.49 (0.34, 0.70)	0.56 (0.34, 0.92)	0.02		0.20 (<.001)	0.24 (<.001)		0.21 (<.001)	0.25 (<.001)
Low Native Hawaiian[4] (n=35)	0.87 (0.63, 1.22)	Ref	Ref		0.10 (<.001)	0.11 (<.001)		0.10 (<.001)	0.20 (<.001)
American Indian / Alaska Native									
High American Indian[5] (n=8)	5.5 (1.1, 22.7)	8.86 (1.64, 50.16)	0.01		4.4 (0.77)	4.8 (0.86)		3.4 (0.55)	9.0 (0.52)
Moderate American Indian[5]	1.4 (1.0, 2.0)	2.15 (1.23, 3.73)	0.01		1.3 (0.72)	1.3 (0.69)		1.1 (0.25)	1.0 (0.05)
Low American Indian[5] (n=27)	0.7 (0.4 1.0)	Ref	Ref		1.6 (0.88)	0.7 (0.79)		2.0 (<.001)	0.3 (.003)
Hispanic									
High Hispanic[6] (n=10)	73.6 (53.4, 87.1)	11.38 (3.78, 35.48)	<.001		35.2 (<.001)	38.4 (<.001)		39.9 (<.001)	46.9 (<.011)
Moderate Hispanic[6] (n=45)	33.2 (28.6 38.1)	2.03 (0.99, 4.20)	0.05		19.5 (<.001)	19.2 (<.001)		17.7 (<.001)	22.0 (<.001)
Low Hispanic[6] (n=23)	19.7 (11.0, 32.7)	Ref	Ref		10.0 (<.025)	13.0 (0.14)		9.7 (0.02)	13.5 (0.19)

* The comparison to cumulative COVID-19 incidence was presented in the supplementary materials (eTable3)

1. States with low proportion of white population include AL, AK, DE, DC, GA, LA, MD, MS, NY, SC and VA. Trials conducted in regions with low, moderate and high white population was defined as trials exclusively, partially and not conducted in the predefined states with low proportion of white population respectively.
2. States with high proportion of black population include AL DE, DC, FL, GA, LA, MD, MS, NY, NCSC, TN and VA. Trials conducted in regions with high, moderate and low Hispanic population was defined as trials exclusively, partially and not conducted in the predefined states with high proportion of black population respectively.
3. States with high proportion of black population include CA HI, MA, NV, NJ, NY and WA. Trials conducted in regions with high, moderate and low Hispanic population was defined as trials exclusively, partially and not conducted in the predefined states with high proportion of Asian population respectively.
4. States with high proportion of Native Hawaiian/ Pacific Islander population include AK, AZ, AR, CA, HI, NV, OR, UT and WA. Trials conducted in regions with high, moderate and low Hawaiian/ Pacific Islander was defined as trials exclusively, partially and not conducted in the predefined states with high proportion of Hawaiian/ Pacific Islander respectively.
5. States with high proportion of American Indian/Native Alaska population include AK, AZ, CA, CO, ID, MT, NE, NV, NM, NC, ND, OK, OR, SD, UT, WA and WY. Trials conducted in regions with high, moderate and low American Indian/Native Alaska was defined as trials exclusively, partially and not conducted in the predefined states with high proportion of American Indian/Native Alaska respectively.
6. States with high proportion of Hispanic population include AZ, CA, CO, FL, NM, NV and TX and. Trials conducted in regions with high, moderate and low Hispanic population was defined as trials exclusively, partially and not conducted in the predefined states with high proportion of Hispanic population respectively.

© 2022 Xiao H et al. *JAMA Intern Med.*

eTable 4. Race/ethnicity representation in COVID-19 clinical trials: results from analyses stratified by locations of clinical trials (COVID-19 incidence as of February 2022)

	Estimated proportion of participants (95 % CI)	Effect of Moderator			Proportion of population (%)			Proportion of cumulative COVID19 incidence as of Feb 2022	
		OR	p-value		Unadjusted (p-value)	Adjusted (p-value)		Unadjusted (p-value)	Adjusted (p-value)
White									
High white[1] (n=7)	74.3 (52.2, 88.4)	1.19 (0.41, 3.40)	0.75		92.5 (0.003)	79.2 (0.58)		79.6 (0.54)	78.9 (0.60)
Moderate white[1] (n=42)	75.7 (71.1, 79.8)	1.29 (0.79, 2.10)	0.31		76.3 (0.79)	75.1 (0.78)		76.0 (0.88)	73.2 (0.27)
Low white[1] (n=29)	70.8 (61.5, 78.7)	Ref	Ref		64.8 (0.20)	68.7 (0.64)		62.0 (0.06)	63.4 (0.12)
Black									
High black[2] (n=12)	18.7 (8.8, 35.3)	1.32 (0.48, 3.55)	0.59		22.8 (0.57)	19.25 (0.93)		25.1 (0.39)	22.6 (0.59)
Moderate black[2] (n=48)	13.1 (10.6, 16.1)	0.86 (0.50, 1.50)	0.61		13.4 (0.82)	13.9 (0.57)		15.0 (0.19)	16.0 (0.07)
Low black[2] (n=18)	14.9 (9.5, 22.5)	Ref	Ref		9.2 (0.03)	10.1 (0.09)		9.9 (0.08)	11.4 (0.24)
Asian									
High Asian[3] (n=9)	7.7 (3.3, 16.7)	3.21 (1.14, 8.86)	0.03		12.4 (0.24)	9.2 (0.67)		11.6 (0.30)	10.0 (0.51)
Moderate Asian[3] (n=40)	5.0 (4.0, 6.2)	2.04 (1.15, 3.64)	0.01		5.9 (0.15)	6.7 (0.008)		4.3 (0.14)	5.5 (0.46)
Low Asian[3] (n=29)	2.5 (1.5, 4.2)	Ref	Ref		3.6 (0.16)	3.7 (0.15)		1.8 (0.22)	2.0 (0.38)
Native Hawaiian / Pacific Islander									
High Native Hawaiian[4] (n=3)	2.23 (0.45, 10.35)	2.60 (0.51, 13.49)	0.26		0.76 (0.19)	0.50 (0.07)		0.98 (0.31)	1.17 (0.43)
Moderate Native Hawaiian[4] (n=40)	0.49 (0.34, 0.70)	0.56 (0.34, 0.92)	0.02		0.20 (<.001)	0.24 (<.001)		0.25 (<.001)	0.27 (0.001)
Low Native Hawaiian[4] (n=35)	0.87 (0.63, 1.22)	Ref	Ref		0.10 (<.001)	0.11 (<.001)		0.10 (<.001)	0.20 (<.001)
American Indian / Alaska Native									
High American Indian[5] (n=8)	5.5 (1.1, 22.7)	8.86 (1.64, 50.16)	0.01		4.4 (0.77)	4.8 (0.86)		3.5 (0.46)	7.9 (0.64)
Moderate American Indian[5] (n=43)	1.4 (1.0, 2.0)	2.15 (1.23, 3.73)	0.01		1.3 (0.72)	1.3 (0.69)		1.1 (0.27)	1.0 (0.11)
Low American Indian[5] (n=27)	0.7 (0.4 1.0)	Ref	Ref		1.6 (0.88)	0.7 (0.79)		2.0 (<.001)	0.3 (<.001)
Hispanic									
High Hispanic[6] (n=10)	73.6 (53.4, 87.1)	11.38 (3.78, 35.48)	<.001		35.2 (<.001)	38.4 (<.001)		36.2 (0.01)	42.8 (<.001)
Moderate Hispanic[6] (n=45)	33.2 (28.6 38.1)	2.03 (0.99, 4.20)	0.05		19.5 (<.001)	19.2 (<.001)		15.4 (<.001)	19.5 (<.001)
Low Hispanic[6] (n=23)	19.7 (11.0, 32.7)	Ref	Ref		10.0 (.025)	13.0 (0.14).		7.9 (0.003)	12.0 (0.09)

7. States with low proportion of white population include AL, AK, DE, DC, GA, LA, MD, MS, NY, SC and VA. Trials conducted in regions with low, moderate and high white population was defined as trials exclusively, partially and not conducted in the predefined states with low proportion of white population respectively.
8. States with high proportion of black population include AL DE, DC, FL, GA, LA, MD, MS, NY, NCSC, TN and VA. Trials conducted in regions with high, moderate and low Hispanic population was defined as trials exclusively, partially and not conducted in the predefined states with high proportion of black population respectively.
9. States with high proportion of black population include CA HI, MA, NV, NJ, NY and WA. Trials conducted in regions with high, moderate and low Hispanic population was defined as trials exclusively, partially and not conducted in the predefined states with high proportion of Asian population respectively.
10. States with high proportion of Native Hawaiian/ Pacific Islander population include AK, AZ, AR, CA, HI, NV, OR, UT and WA. Trials conducted in regions with high, moderate and low Hawaiian/ Pacific Islander was defined as trials exclusively, partially and not conducted in the predefined states with high proportion of Hawaiian/ Pacific Islander respectively.

© 2022 Xiao H et al. *JAMA Intern Med.*

11. States with high proportion of American Indian/Native Alaska population include AK, AZ, CA, CO, ID, MT, NE, NV, NM, NC, ND, OK, OR, SD, UT, WA and WY. Trials conducted in regions with high, moderate and low American Indian/Native Alaska was defined as trials exclusively, partially and not conducted in the predefined states with high proportion of American Indian/Native Alaska respectively.
12. States with high proportion of Hispanic population include AZ, CA, CO FL, NM, TX and NV. Trials conducted in regions with high, moderate and low Hispanic population was defined as trials exclusively, partially and not conducted in the predefined states with high proportion of Hispanic population respectively.

© 2022 Xiao H et al. *JAMA Intern Med.*

eTable 5A. Sensitivity analysis results for the estimate of female proportion using the "leave one out" method

Study Omitted	Estimated Female Proportion (%)	95% CI		Study Omitted	Estimated Female Proportion (%)	95% CI
Abella et al., 2021	45.1	42.9 - 47.1		NCT04348500	45.3	43.1 - 47.4
Ali et al., 2021	45.2	43.0 - 47.3		NCT04349098	45.3	43.1 - 47.4
Bar et al., 2021	45.2	43.0 - 47.3		NCT04351243	45.4	43.3 - 47.5
Barnabas et al., 2021	45.3	43.1 - 47.4		NCT04353037	45.2	43.0 - 47.3
BennettGuerrero et al.,	45.3	43.1 - 47.4		NCT04354870	45.2	43.0 - 47.3
Bradfute et al., 2020	45.3	43.1 - 47.4		NCT04355767	45.2	43.0 - 47.3
Chu et al., 2021	45.1	42.9 - 47.1		NCT04356937	45.3	43.1 - 47.4
Dayya et al., 2021	45.4	43.2 - 47.5		NCT04357730	45.4	43.3 - 47.5
Donato et al., 2021	45.2	43.0 - 47.3		NCT04358068	45.2	43.0 - 47.3
Dunkle LM., 2021	45.2	43.0 - 47.3		NCT04358081	45.3	43.1 - 47.4
Elamir et al., 2022	45.2	43.0 - 47.3		NCT04359797	45.3	43.1 - 47.4
Falsey AR., 2021	45.3	43.1 - 47.4		NCT04363437	45.3	43.1 - 47.4
Goepfert et al., 2021	45.2	43.0 - 47.3		NCT04363736	45.3	43.1 - 47.4
Greenbaum et al., 2021	45.3	43.1 - 47.4		NCT04365153	45.4	43.3 - 47.5
Gupta et al., 2021	45.1	43.0 - 47.2		NCT04365699	45.6	43.4 - 47.6
Hess et al., 2021	45.2	43.0 - 47.3		NCT04365985	45.3	43.2 - 47.4
Huang et al., 2021	45.3	43.1 - 47.4		NCT04368260	45.2	43.1 - 47.3
Humeniuk et al., 2020	45.3	43.1 - 47.4		NCT04372186	45.3	43.1 - 47.4
Jackson et al., 2020	45.2	43.0 - 47.3		NCT04374019	45.2	43.0 - 47.3
Kaur et al., 2021	45.4	43.2 - 47.5		NCT04377620	45.4	43.2 - 47.5
Lanzoni et al., 2021	45.3	43.1 - 47.4		NCT04377711	45.2	43.0 - 47.3
Lundgren et al., 2021	45.3	43.1 - 47.4		NCT04380688	45.4	43.2 - 47.5
Miller et al., 2020	45.2	43.0 - 47.3		NCT04388826	45.4	43.2 - 47.5
O'Brien et al., 2021	45.2	43.0 - 47.3		NCT04399980	45.4	43.2 - 47.4
Perepu et al., 2021	45.3	43.1 - 47.4		NCT04401293	45.3	43.1 - 47.4
Baden et al., 2021	45.2	43.0 - 47.4		NCT04401579	45.4	43.2 - 47.5
Salazar et al., 2020	45.2	43.0 - 47.3		NCT04402970	45.3	43.1 - 47.4
Sengupta et al., 2020	45.3	43.1 - 47.4		NCT04405570	45.2	43.0 - 47.3
Shroff RT et al., 2021	45.4	43.2 - 47.5		NCT04409509	45.3	43.1 - 47.4
Skipper et al., 2020	45.1	43.0 - 47.2		NCT04411628	45.4	43.2 - 47.5
Strohbehn et al., 2021	45.2	43.1 - 47.3		NCT04411667	45.3	43.1 - 47.4
Taylor et al., 2021	45.4	43.2 - 47.5		NCT04411680	45.2	43.0 - 47.3
Thomas et al., 2021	45.1	42.9 - 47.2		NCT04421404	45.2	43.0 - 47.3
Thomas SJ., 2021	45.2	43.0 - 47.3		NCT04425252	45.3	43.1 - 47.4
Uckun et al., 2021	45.2	43.0 - 47.3		NCT04425538	45.4	43.2 - 47.5
Weinreich et al., 2021	45.2	43.0 - 47.3		NCT04425720	45.1	42.9 - 47.2
NCT03852537	45.3	43.1 - 47.4		NCT04432272	45.4	43.2 - 47.5
NCT04280705	45.4	43.2 - 47.5		NCT04435184	45.3	43.1 - 47.4
NCT04292899	45.4	43.2 - 47.5		NCT04456153	45.3	43.2 - 47.4
NCT04308668	45.2	43.0 - 47.3		NCT04492475	45.3	43.1 - 47.4
NCT04315298	45.4	43.3 - 47.5		NCT04494646	45.3	43.2 - 47.4
NCT04317040	45.4	43.2 - 47.5		NCT04498247	45.2	43.0 - 47.2
NCT04320615	45.5	43.3 - 47.6		NCT04498273	45.1	42.9 - 47.2
NCT04328467	45.2	43.0 - 47.3		NCT04501952	45.2	43.0 - 47.3
NCT04331899	45.3	43.1 - 47.4		NCT04502472	45.1	43.0 - 47.2
NCT04332081	45.7	43.6 - 47.7		NCT04504032	45.1	42.9 - 47.2
NCT04332107	45.1	42.9 - 47.1		NCT04524507	45.4	43.2 - 47.5
NCT04332991	45.3	43.1 - 47.4		NCT04569786	45.2	43.0 - 47.3
NCT04335552	45.3	43.2 - 47.4		NCT04583592	45.1	43.0 - 47.2
NCT04338009	45.3	43.1 - 47.4		NCT04685213	45.3	43.1 - 47.4
NCT04340557	45.3	43.1 - 47.4		NCT04701658	45.2	43.0 - 47.3
NCT04342663	45.0	42.9 - 47.1		NCT04706416	45.3	43.1 - 47.4
NCT04342897	45.4	43.2 - 47.5		NCT04871815	45.2	43.1 - 47.3
NCT04343261	45.3	43.1 - 47.4				
NCT04343989	45.4	43.3 - 47.5				
NCT04347954	45.2	43.0 - 47.3		**Total**	**45.3**	**43.2 - 47.4**

© 2022 Xiao H et al. *JAMA Intern Med.*

eTable 5B. Sensitivity analysis results for the estimate of Hispanic/Latino ethnicity proportion using the "leave one out" method

Study Omitted	Estimated Hispanic Proportion (%)	95% CI		Study Omitted	Estimated Hispanic Proportion (%)	95% CI
Ali et al., 2021	34.6	28.2 - 41.6		NCT04355767	34.2	27.7 - 41.2
Bar et al., 2021	34.9	28.5 - 41.8		NCT04356937	33.9	27.5 - 40.9
Barnabas et al., 2021	34.3	27.8 - 41.4		NCT04357730	33.7	27.3 - 40.6
Bradfute et al., 2020	34.5	28.1 - 41.5		NCT04358068	34.2	27.7 - 41.2
Dunkle LM., 2021	34.4	27.9 - 41.4		NCT04359797	33.3	27.2 - 40.0
Falsey AR., 2021	34.3	27.8 - 41.4		NCT04363736	34.2	27.7 - 41.3
Ghandehari et al., 2021	33.8	27.4 - 40.7		NCT04365985	34.9	28.5 - 41.8
Goepfert et al., 2021	34.6	28.1 - 41.6		NCT04372186	33.8	27.4 - 40.8
Gupta et al., 2021	33.7	27.3 - 40.7		NCT04374019	34.5	28.1 - 41.5
Hess et al., 2021	34.6	28.2 - 41.5		NCT04377620	34.3	27.8 - 41.3
Humeniuk et al., 2020	33.1	27.2 - 39.4		NCT04377711	34.0	27.5 - 41.0
Jackson et al., 2020	34.5	28.0 - 41.5		NCT04380688	34.0	27.5 - 41.0
Lanzoni et al., 2021	33.4	27.2 - 40.1		NCT04388826	34.1	27.6 - 41.1
Miller et al., 2020	34.6	28.1 - 41.6		NCT04401579	33.9	27.4 - 40.9
O'Brien et al., 2021	34.0	27.5 - 41.1		NCT04405570	34.0	27.5 - 41.0
Baden et al., 2021	34.4	27.9 - 41.4		NCT04409509	34.4	27.9 - 41.4
Taylor et al., 2021	34.3	27.8 - 41.3		NCT04411628	33.8	27.4 - 40.7
Thomas SJ., 2021	34.3	27.8 - 41.3		NCT04411667	33.5	27.2 - 40.2
Weinreich et al., 2021	34.0	27.5 - 41.1		NCT04411680	34.2	27.7 - 41.2
NCT04280705	34.3	27.8 - 41.3		NCT04421404	33.7	27.3 - 40.6
NCT04292899	34.1	27.6 - 41.1		NCT04425720	33.7	27.3 - 40.6
NCT04308668	35.0	28.6 - 41.8		NCT04432272	34.9	28.5 - 41.7
NCT04315298	34.1	27.6 - 41.2		NCT04456153	33.6	27.3 - 40.5
NCT04317040	34.1	27.6 - 41.1		NCT04492475	34.1	27.6 - 41.2
NCT04320615	34.1	27.6 - 41.2		NCT04494646	34.2	27.7 - 41.2
NCT04335552	33.9	27.5 - 40.9		NCT04498247	34.5	28.1 - 41.5
NCT04338009	33.8	27.4 - 40.8		NCT04501952	34.0	27.5 - 41.0
NCT04342663	35.0	28.7 - 41.9		NCT04502472	34.2	27.7 - 41.2
NCT04342897	34.0	27.5 - 41.0		NCT04504032	34.4	27.9 - 41.4
NCT04343261	34.3	27.8 - 41.3		NCT04524507	34.1	27.7 - 41.2
NCT04343989	34.2	27.7 - 41.3		NCT04569786	34.0	27.5 - 41.0
NCT04348500	33.9	27.5 - 40.9		NCT04583592	33.8	27.3 - 40.7
NCT04349098	34.0	27.6 - 41.1		NCT04701658	33.9	27.5 - 40.9
NCT04351243	34.0	27.5 - 41.0		NCT04706416	33.3	27.2 - 40.0
NCT04353037	34.7	28.2 - 41.6				
NCT04354870	34.8	28.4 - 41.7		**Total**	**34.1**	**27.8 - 41.1**

© 2022 Xiao H et al. *JAMA Intern Med.*

eTable 5C. Sensitivity analysis results for the estimate of racial proportions using the “leave one out” method

	White		Black		Asian		Hawaiian/ Pacific Islander		American Indian/ Native Alaska	
**Study Omitted**	**Proportion (%)**	**95% CI**	**Proportion (%)**	**95% CI**	**Proportion (%)**	**95% CI**	**Proportion (%)**	**95% CI**	**Proportion (%)**	**95% CI**
Ali et al., 2021	73.7	69.4 - 77.5	14.6	12.0 - 17.5	4.4	3.4 - 5.5	0.64	0.49 - 0.82	1.4	1.0 - 1.8
Bar et al., 2021	74.3	70.1 - 77.9	13.9	11.5 - 16.7	4.4	3.5 - 5.5	0.61	0.46 - 0.80	1.3	0.9 - 1.8
Barnabas et al., 2021	74.2	69.9 - 77.9	14.4	11.8 - 17.3	4.4	3.4 - 5.4	0.62	0.47 - 0.81	1.3	0.9 - 1.7
BennettGuerrero et al.,	73.6	69.4 - 77.3	14.4	11.8 - 17.3	4.5	3.5 - 5.5	0.61	0.46 - 0.80	1.3	0.9 - 1.7
Bradfute et al., 2020	74.3	70.2 - 77.9	14.3	11.8 - 17.2	4.4	3.5 - 5.5	0.61	0.46 - 0.79	1.3	0.9 - 1.6
Chu et al., 2021	73.3	69.2 - 77.0	14.6	12.0 - 17.5	4.6	3.6 - 5.6	0.62	0.47 - 0.81	1.4	0.9 - 1.8
Dunkle LM., 2021	73.9	69.6 - 77.6	14.3	11.7 - 17.2	4.4	3.5 - 5.5	0.64	0.48 - 0.83	1.3	0.9 - 1.7
Falsey AR., 2021	73.8	69.5 - 77.6	14.4	11.8 - 17.3	4.4	3.5 - 5.5	0.63	0.48 - 0.83	1.3	0.9 - 1.7
Ghandehari et al., 2021	73.8	69.6 - 77.6	14.3	11.7 - 17.2	4.4	3.5 - 5.5	0.61	0.46 - 0.79	1.3	0.9 - 1.7
Goepfert et al., 2021	73.7	69.4 - 77.4	14.5	11.9 - 17.4	4.4	3.4 - 5.5	0.62	0.47 - 0.81	1.3	0.9 - 1.8
Greenbaum et al., 2021	73.7	69.5 - 77.4	14.4	11.8 - 17.3	4.4	3.5 - 5.5	0.61	0.46 - 0.79	1.3	0.9 - 1.7
Gupta et al., 2021	73.6	69.4 - 77.4	14.4	11.8 - 17.4	4.4	3.4 - 5.5	0.62	0.47 - 0.81	1.4	1.0 - 1.8
Hess et al., 2021	74.3	70.2 - 78.0	13.9	11.5 - 16.6	4.5	3.5 - 5.5	0.61	0.46 - 0.79	1.3	0.9 - 1.7
Humeniuk et al., 2020	73.7	69.4 - 77.4	14.3	11.7 - 17.2	4.5	3.5 - 5.6	0.62	0.47 - 0.80	1.3	0.9 - 1.8
Jackson et al., 2020	73.6	69.4 - 77.4	14.4	11.8 - 17.3	4.5	3.5 - 5.5	0.61	0.46 - 0.80	1.3	0.9 - 1.7
Lanzoni et al., 2021	73.7	69.5 - 77.5	14.3	11.7 - 17.2	4.5	3.5 - 5.5	0.61	0.46 - 0.79	1.3	0.9 - 1.7
Miller et al., 2020	74.2	70.0 - 77.9	14.0	11.5 - 16.9	4.4	3.5 - 5.5	0.61	0.46 - 0.79	1.3	0.9 - 1.7
O'Brien et al., 2021	73.7	69.4 - 77.5	14.3	11.7 - 17.3	4.5	3.5 - 5.6	0.63	0.47 - 0.82	1.4	1.0 - 1.8
Baden et al., 2021"	73.7	69.4 - 77.5	14.3	11.7 - 17.2	4.4	3.5 - 5.5	0.63	0.48 - 0.83	1.3	0.9 - 1.8
Taylor et al., 2021	74.3	70.2 - 78.0	13.9	11.5 - 16.7	4.5	3.5 - 5.6	0.61	0.46 - 0.80	1.3	0.9 - 1.8
Thomas et al., 2021	73.9	69.7 - 77.7	14.1	11.6 - 17.0	4.5	3.6 - 5.6	0.62	0.47 - 0.81	1.3	0.9 - 1.8
Thomas SJ., 2021	73.7	69.5 - 77.5	14.3	11.7 - 17.3	4.4	3.5 - 5.5	0.64	0.48 - 0.83	1.3	0.9 - 1.8
Uckun et al., 2021	74.0	69.8 - 77.8	14.0	11.5 - 16.9	4.5	3.5 - 5.6	0.61	0.46 - 0.79	1.3	0.9 - 1.8
Weinreich et al., 2021	73.6	69.3 - 77.4	14.5	11.9 - 17.4	4.4	3.5 - 5.5	0.63	0.48 - 0.82	1.3	0.9 - 1.7
NCT03852537	73.8	69.5 - 77.6	14.4	11.8 - 17.3	4.4	3.5 - 5.5	0.61	0.46 - 0.80	1.3	0.9 - 1.7
NCT04280705	74.1	69.8 - 77.8	14.1	11.6 - 17.0	4.3	3.4 - 5.4	0.62	0.47 - 0.81	1.3	0.9 - 1.8
NCT04292899	74.1	69.9 - 77.9	14.2	11.6 - 17.1	4.4	3.4 - 5.4	0.61	0.46 - 0.79	1.3	0.9 - 1.8
NCT04308668	74.1	69.8 - 77.8	14.5	11.9 - 17.4	4.3	3.5 - 5.3	0.61	0.46 - 0.80	1.4	1.0 - 1.8
NCT04315298	74.1	69.8 - 77.8	14.1	11.5 - 17.0	4.4	3.4 - 5.4	0.62	0.46 - 0.81	1.4	1.0 - 1.8
NCT04317040	74.0	69.7 - 77.8	14.1	11.5 - 17.0	4.5	3.5 - 5.6	0.62	0.47 - 0.80	1.3	0.9 - 1.8
NCT04320615	74.0	69.7 - 77.8	14.2	11.6 - 17.1	4.4	3.4 - 5.4	0.59	0.44 - 0.76	1.3	0.9 - 1.7
NCT04335552	74.0	69.7 - 77.7	14.2	11.6 - 17.0	4.4	3.5 - 5.5	0.60	0.46 - 0.79	1.3	0.9 - 1.7
NCT04342663	73.9	69.7 - 77.7	14.1	11.6 - 17.0	4.5	3.5 - 5.5	0.62	0.47 - 0.80	1.3	0.9 - 1.8
NCT04342897	73.8	69.6 - 77.6	14.2	11.6 - 17.2	4.4	3.5 - 5.5	0.61	0.46 - 0.79	1.3	0.9 - 1.8
NCT04343261	74.3	70.1 - 77.9	14.1	11.5 - 16.9	4.5	3.5 - 5.5	0.61	0.46 - 0.80	1.3	0.9 - 1.7
NCT04343989	74.1	69.9 - 77.8	14.1	11.5 - 17.0	4.3	3.4 - 5.4	0.62	0.46 - 0.80	1.3	0.9 - 1.8
NCT04348500	73.9	69.6 - 77.7	14.3	11.7 - 17.2	4.4	3.4 - 5.4	0.61	0.46 - 0.79	1.3	0.9 - 1.7
NCT04349098	74.2	70.0 - 77.9	14.1	11.5 - 17.0	4.4	3.5 - 5.5	0.61	0.46 - 0.79	1.3	0.9 - 1.8
NCT04351243	74.0	69.7 - 77.8	14.1	11.6 - 17.0	4.4	3.4 - 5.5	0.62	0.47 - 0.80	1.3	0.9 - 1.8

© 2022 Xiao H et al. *JAMA Intern Med.*

NCT04353037	73.9	69.7 - 77.7		14.3	11.7 - 17.3		4.4	3.4 - 5.4		0.58	0.44 - 0.75		1.3	0.9 - 1.7
NCT04354870	73.8	69.6 - 77.6		14.4	11.8 - 17.3		4.3	3.4 - 5.4		0.61	0.46 - 0.80		1.3	0.9 - 1.7
NCT04355767	74.0	69.7 - 77.8		14.2	11.6 - 17.1		4.4	3.5 - 5.5		0.62	0.47 - 0.81		1.4	1.0 - 1.8
NCT04356937	74.2	70.0 - 77.9		14.2	11.6 - 17.1		4.4	3.5 - 5.5		0.62	0.46 - 0.80		1.3	0.9 - 1.8
NCT04357730	73.7	69.5 - 77.5		14.4	11.8 - 17.3		4.5	3.5 - 5.5		0.61	0.46 - 0.79		1.3	0.9 - 1.7
NCT04358068	73.7	69.5 - 77.5		14.3	11.7 - 17.2		4.4	3.5 - 5.5		0.61	0.46 - 0.79		1.3	0.9 - 1.7
NCT04358081	74.0	69.8 - 77.8		14.2	11.6 - 17.1		4.4	3.5 - 5.5		0.59	0.45 - 0.77		1.3	0.9 - 1.7
NCT04359797	74.0	69.7 - 77.8		14.2	11.6 - 17.1		4.5	3.5 - 5.5		0.62	0.47 - 0.81		1.4	1.0 - 1.8
NCT04363736	74.2	70.0 - 77.9		14.0	11.5 - 16.8		4.4	3.5 - 5.5		0.61	0.46 - 0.79		1.3	0.9 - 1.7
NCT04365985	74.0	69.7 - 77.8		14.2	11.6 - 17.1		4.5	3.5 - 5.6		0.61	0.46 - 0.80		1.3	0.9 - 1.8
NCT04372186	74.1	69.9 - 77.8		14.2	11.6 - 17.1		4.5	3.6 - 5.6		0.62	0.47 - 0.81		1.3	0.9 - 1.6
NCT04374019	73.7	69.5 - 77.5		14.3	11.8 - 17.2		4.4	3.5 - 5.5		0.61	0.46 - 0.79		1.3	0.9 - 1.7
NCT04377620	73.8	69.5 - 77.6		14.2	11.7 - 17.2		4.4	3.5 - 5.5		0.62	0.47 - 0.80		1.3	0.9 - 1.8
NCT04377711	73.7	69.4 - 77.4		14.3	11.7 - 17.2		4.6	3.6 - 5.7		0.61	0.46 - 0.80		1.4	1.0 - 1.8
NCT04380688	74.0	69.7 - 77.7		14.2	11.6 - 17.1		4.5	3.5 - 5.5		0.61	0.46 - 0.80		1.3	0.9 - 1.7
NCT04388826	73.6	69.4 - 77.4		14.4	11.8 - 17.3		4.5	3.5 - 5.5		0.60	0.46 - 0.78		1.3	0.9 - 1.7
NCT04401293	74.2	69.9 - 77.9		14.0	11.5 - 16.9		4.4	3.4 - 5.4		0.62	0.47 - 0.80		1.3	0.9 - 1.8
NCT04401579	74.0	69.8 - 77.8		14.2	11.6 - 17.1		4.3	3.4 - 5.4		0.60	0.45 - 0.78		1.3	0.9 - 1.8
NCT04405570	73.7	69.4 - 77.4		14.4	11.8 - 17.4		4.5	3.5 - 5.5		0.62	0.47 - 0.80		1.3	0.9 - 1.8
NCT04409509	73.7	69.5 - 77.5		14.3	11.7 - 17.2		4.4	3.5 - 5.5		0.61	0.46 - 0.80		1.3	0.9 - 1.8
NCT04411628	73.7	69.5 - 77.5		14.3	11.7 - 17.2		4.4	3.5 - 5.5		0.61	0.46 - 0.79		1.3	0.9 - 1.7
NCT04411680	73.7	69.4 - 77.4		14.4	11.8 - 17.3		4.5	3.5 - 5.5		0.61	0.46 - 0.79		1.3	0.9 - 1.7
NCT04421404	74.1	69.9 - 77.8		14.3	11.7 - 17.2		4.3	3.4 - 5.4		0.61	0.46 - 0.79		1.3	0.9 - 1.7
NCT04425252	74.0	69.7 - 77.8		14.2	11.6 - 17.1		4.5	3.5 - 5.5		0.61	0.46 - 0.79		1.3	0.9 - 1.7
NCT04432272	73.6	69.4 - 77.4		14.3	11.7 - 17.3		4.5	3.5 - 5.5		0.61	0.46 - 0.80		1.3	0.9 - 1.7
NCT04435184	74.2	69.9 - 77.9		14.0	11.5 - 16.8		4.5	3.5 - 5.5		0.61	0.46 - 0.80		1.3	0.9 - 1.7
NCT04456153	73.9	69.6 - 77.6		14.3	11.7 - 17.2		4.5	3.5 - 5.5		0.61	0.46 - 0.80		1.3	0.9 - 1.8
NCT04492475	74.0	69.7 - 77.7		14.2	11.6 - 17.1		4.4	3.4 - 5.4		0.60	0.45 - 0.79		1.3	0.9 - 1.8
NCT04494646	73.8	69.6 - 77.6		14.3	11.7 - 17.2		4.4	3.5 - 5.5		0.61	0.46 - 0.79		1.3	0.9 - 1.7
NCT04498247	73.3	69.2 - 76.9		14.6	12.1 - 17.5		4.6	3.6 - 5.6		0.62	0.47 - 0.81		1.4	1.0 - 1.8
NCT04498273	73.7	69.4 - 77.5		14.3	11.7 - 17.2		4.5	3.6 - 5.6		0.61	0.46 - 0.80		1.4	0.9 - 1.8
NCT04501952	73.8	69.5 - 77.5		14.4	11.8 - 17.3		4.5	3.5 - 5.6		0.62	0.47 - 0.81		1.3	0.9 - 1.7
NCT04502472	73.8	69.5 - 77.6		14.4	11.8 - 17.3		4.4	3.4 - 5.4		0.61	0.46 - 0.80		1.3	0.9 - 1.8
NCT04504032	73.6	69.3 - 77.4		14.4	11.8 - 17.3		4.6	3.6 - 5.7		0.62	0.47 - 0.81		1.3	0.9 - 1.8
NCT04524507	74.1	69.8 - 77.8		14.1	11.5 - 16.9		4.5	3.5 - 5.5		0.61	0.46 - 0.79		1.3	0.9 - 1.7
NCT04569786	73.7	69.4 - 77.5		14.3	11.7 - 17.2		4.5	3.5 - 5.6		0.62	0.47 - 0.81		1.3	0.9 - 1.8
NCT04583592	73.8	69.5 - 77.6		14.2	11.6 - 17.1		4.5	3.5 - 5.6		0.62	0.47 - 0.81		1.3	0.9 - 1.8
NCT04685213	74.4	70.3 - 78.0		14.1	11.6 - 17.0		4.4	3.5 - 5.5		0.61	0.46 - 0.79		1.3	0.9 - 1.7
NCT04701658	73.7	69.4 - 77.5		14.4	11.8 - 17.3		4.5	3.5 - 5.6		0.61	0.46 - 0.79		1.3	0.9 - 1.7
**Total**	**73.9**	**69.7 - 77.7**		**14.3**	**11.8 - 17.2**		**4.4**	**3.6 - 5.6**		**0.61**	**0.47 - 0.87**		**1.3**	**1.0 - 1.8**

© 2022 Xiao H et al. *JAMA Intern Med.*

eTable 6. Sex and race/ethnicity representation in COVID-19 clinical trials solely conducted among adults in the US

		Estimated proportion of participants (95%)	Effect of Moderator		Proportion of population (%)		Proportion of cumulative COVID19 incidence as of April 2021		Proportion of cumulative COVID19 incidence as of Feb 2022	
			OR (95% CI)	p-value	Unadjusted (p-value)	Adjusted (p-value)	Unadjusted (p-value)	Adjusted (p-value)	Unadjusted (p-value)	Adjusted (p-value)
**Female**										
Overall (n=85)		44.5 (41.9, 47.2)			51.7 (<.001)		52.7 (<.001)		53.8 (<.001)	
Purpose	Prevention (n=8)	48.5 (40.3, 56.8)	1.20 (0.84,	0.31	51.7 (0.22)		52.7 (0.09)		53.8 (0.03)	
	Treatment (n=77)	44.0 (41.2, 46.8)	Ref	Ref	51.7 (<.001)		52.7 (<.001)		53.8 (<.001)	
Phase	Phase 1/ Phase 2	(44.9 41.5, 48.3)	Ref	Ref	51.7 (<.001)		52.7 (<.001)		53.8 (<.001)	
	Phase 3/ Phase 4	44.3 (40.0, 48.6)	0.98 (0.78,	0.83	51.7 (<.001)		52.7 (<.001)		53.8 (<.001)	
Primary Funder	Government (n=4)	43.5 (32.5, 55.2)	0.95 (0.58,	0.86	51.7 (0.02)		52.7 (0.008)		53.8 (0.003)	
	Industry (n=28)	44.6 (40.8, 48.4)	Ref	Ref	51.7 (<.001)		52.7 (<.001)		53.8 (<.001)	
	Other (n=52)	44.5 (40.7, 48.3)	1.00 (0.80,	0.97	51.7 (<.001)		52.7 (<.001)		53.8 (<.001)	
**Race: white**										
Overall (n=62)		72.8 (67.7, 77.3)			81.3 (<.001)	80.6 (<.001)	77.7 (0.03)	76.6 (0.10)	76.6 (0.10)	74.7 (0.43)
Purpose	Prevention (n=7)	88.4 (79.7, 93.7)	3.35 (1.67,	<.001	81.3 (0.09)	79.8 (0.05)	77.7 (0.02)	77.2 (0.02)	76.6 (0.01)	75.0 (0.006)
	Treatment (n=55)	69.5 (64.5, 74.2)	Ref	Ref	81.3 (<.001)	82.7 (<.001)	77.7 (<.001)	74.9 (0.02)	76.6 (0.002)	73.7 (0.08)
Phase	Phase 1/ Phase 2	75.0 (68.4, 80.7)	Ref	Ref	81.3 (0.03)	80.7 (0.04)	77.7 (0.38)	78.1 (0.31)	76.6 (0.61)	76.0 (0.75)
	Phase 3/ Phase 4	65.7 (58.7, 72.1)	0.64 (0.41,	0.04	81.3 (<0.001)	80.4 (<.001)	77.7 (<.001)	76.4 (<.001)	76.6 (<.001)	74.4 (0.006)
Primary Funder	Government (n=4)	77.5 (54.6, 90.8)	1.05 (0.35,	0.93	81.3 (0.67)	80.0 (0.78)	77.7 (0.98)	73.5 (0.69)	76.6 (0.92)	72.0 (0.59)
	Industry (n=26)	76.6 (69.7, 82.4)	Ref	Ref	81.3 (0.12)	80.2 (0.24)	77.7 (0.74)	76.9 (0.93)	76.6 (0.99)	74.8 (0.59)
	Other (n=32)	68.3 (60.3, 75.4)	65.7 (39.7,	0.010	81.3 (<.001)	83.9 (<.001)	77.7 (0.007)	75.9 (0.03)	76.6 (0.02)	75.0 (0.06)
**Race: Black**										
Overall (n=62)	*Overall (n=62)*	15.6 (1.6, 19.2)			11.1 (0.002)	12.6 (0.05)	14.3 (0.42)	16.0 (0.82)	14.7 (0.58)	16.5 (0.61)
Purpose	Prevention (n=7)	6.5 (3.1, 13.3)	0.32 (0.14,	0.006	11.1 (0.15)	13.6 (0.04)	14.3 (0.03)	16.0 (0.01)	14.7 (0.02)	16.5 (0.009)
	Treatment (n=55)	17.9 (14.7, 21.7)	Ref	Ref	11.1 (<0.001)	10.0 (<.001)	14.3 (0.03)	16.1 (0.29)	14.7 (0.05)	16.2 (0.32)

© 2022 Xiao H et al. *JAMA Intern Med.*

Phase	Phase 1/ Phase 2	14.3 (10.6, 19.0)	Ref	Ref		11.1 (0.10)	12.5		14.3 (0.99)	14.7 (0.85)		14.7 (0.85)	15.2 (0.16)
	Phase 3/ Phase 4	18.4 (14.1, 23.5)	1.35 (0.84,	0.21		11.1 (<.001)	12.7 (0.006)		14.3 (0.06)	16.2 (0.35)		14.7 (0.10)	16.6 (0.35)
Primary Funder	Government (n=4)	15.2 (7.4, 28.6)	1.06 (0.44,	0.88		11.1 (0.38)	12.4 (0.57)		14.3 (0.87)	16.4 (0.82)		14.7 (0.93)	16.6 (0.79)
	Industry (n=26)	14.3 (10.3, 19.6)	Ref	Ref		11.1 (0.13)	13.1 (0.58)		14.3 (0.99)	16.0 (0.50)		14.7 (0.88)	16.6 (0.36)
	Other (n=32)	17.0 (12.4, 23.0)	1.23 (0.73,	0.45		11.1 (0.009)	9.5 (<.001)		14.3 (0.27)	15.5 (0.56)		14.7 (0.36)	15.6 (0.58)
**Race: Asian**													
Overall (n=62)		4.3 (3.3, 5.6)				5.3 (0.13)	4.7 (0.52)		3.8 (0.34)	3.6 (0.18)		4.2 (0.90)	4.0 (0.62)
Purpose	Prevention (n=7)	2.9 (1.5, 5.5)	0.62 (0.30,	0.20		5.3 (0.07)	4.7 (0.15)		3.8 (0.43)	3.0 (0.95)		4.2 (0.26)	3.5 (0.58)
	Treatment (n=55)	4.6 (3.5, 6.1)	Ref	Ref		5.3 (0.35)	4.7 (0.87)		3.8 (0.17)	5.0 (0.59)		4.2 (0.55)	5.0 (0.22)
Phase	Phase 1/ Phase 2	3.6 (2.6, 5.1)	Ref	Ref		5.3 (0.03)	4.5 (0.21)		3.8 (0.80)	3.6 (0.95)		4.2 (0.37)	4.2 (0.43)
	Phase 3/ Phase 4	6.6 (4.3, 9.8)	1.86 (1.07,	0.03		5.3 (0.30)	4.8 (0.13)		3.8 (0.01)	3.6 (0.004)		4.2 (0.04)	4.1 (0.02)
Primary Funder	Government (n=4)	13.6 (12.1, 15.3)	4.06 (2.80,	<.001		5.3 (<.001)	6.5 (<.001)		3.8 (<.001)	5.4 (<.001)		4.2 (<.001)	5.9 (<.001)
	Industry (n=26)	3.7 (2.7, 5.2)	Ref	Ref		5.3 (0.04)	4.7 (0.18)		3.8 (0.93)	3.3 (0.44)		4.2 (0.46)	3.8 (0.95)
	Other (n=32)	4.4 (29, 6.6)	1.18 (0.68,	0.55		5.3 (0.39)	4.0 (0.68)		3.8 (0.49)	4.7 (0.75)		4.2 (0.87)	5.1 (0.48)
**Race: Native Hawaiian / Pacific Islander**													
Overall (n=62)		0.80 (0.59, 1.07)				0.24 (<.001)	0.21 (<.001)		0.20 (<.001)	0.20 (<.001)		0.24 (<.001)	0.21 (<.001)
Purpose	Prevention (n=7)	0.20 (0.19, 0.30)	0.24 (0.16,	<.001		0.24 (0.72)	0.21 (0.36)		0.20 (0.19)	0.20 (0.19)		0.24 (0.72)	0.22 (0.60)
	Treatment (n=55)	1.0 (0.73, 1.30)	Ref	Ref		0.24 (<.001)	0.19 (<.001)		0.20 (<.001)	0.20 (<.001)		0.24 (<.001)	0.22 (<.001)
Phase	Phase 1/ Phase 2	1.0 (0.72, 1.48)	Ref	Ref		0.24 (<.001)	0.19 (<.001)		0.20 (<.001)	0.13 (<.001)		0.24 (<.001)	0.15 (<.001)
	Phase 3/ Phase 4	0.60 (0.35, 1.05)	0.58 (0.29,	0.11		0.24 (0.001)	0.21 (<.001)		0.20 (<.001)	0.21 (<.001)		0.24 (0.001)	0.23 (<.001)
Primary Funder	Government (n=4)	0.98 (0.41, 2.34)	1.24 (0.45,	0.66		0.24 (0.002)	0.24 (0.002)		0.20 (0.004)	0.33 (0.01)		0.24 (0.002)	0.36 (0.002)
	Industry (n=26)	0.78 (0.48, 1.27)	Ref	Ref		0.24 (<.001)	0.21 (<.001)		0.20 (<.001)	0.20 (<.001)		0.24 (<.001)	0.21 (<.001)
	Other (n=32)	0.74 (0.49, 1.13)	0.95 (050, 1.82)	0.87		0.24 (<.001)	0.18 (<.001)		0.20 (<.001)	0.16 (<.001)		0.24 (<.001)	0.17 (<.001)
**Race: American Indian / Alaska Native**													
Overall (n=62)		1.3 (0.9, 1.7)				1.9 (0.04)	1.9 (0.04)		1.0 (0.18)	0.8 (<.001)		1.0 (0.18)	0.9 (<.001)
Purpose	Prevention (n=7)	0.8 (0.7, 0.9)	0.56 (0.36,	0.007		1.9 (<.001)	1.8 (<.001)		1.04 (<.001)	0.8 (0.57)		1.0 (<.001)	0.9 (0.02)

© 2022 Xiao H et al. *JAMA Intern Med.*

	Treatment (n=55)	1.5 (1.0, 2.2)	Ref	Ref		1.9 (0.15)	2.5 (0.008)		1.0 (0.11)	0.7 (0.001)		1.0 (0.11)	0.7 (<.001)
Phase	Phase 1/ Phase 2	1.5 (1.0, 2.2)	Ref	Ref		1.9 (0.19)	2.1 (0.08)		1.0 (0.08)	0.8 (0.006)		1.0 (0.08)	0.9 (0.02)
	Phase 3/ Phase 4	1.1 (0.5, 2..5)	0.76 (0.32,	0.55		1.9 (0.17)	2.0 (0.15)		1.0 (0.81)	0.8 (0.36)		1.0 (0.81)	1.2 (0.95)
Primary Funder	Government (n=4)	1.1 (0.7, 1.8)	0.88 (0.43,	0.73		1.9 (0.01)	2.0 (0.01)		1.0 (0.79)	0.7 (0.07)		1.0 (0.79)	0.9 (0.27)
	Industry (n=26)	1.3 (0.7, 2.1)	Ref	Ref		1.9 (0.11)	1.9 (0.14)		1.0 (0.49)	0.8 (0.11)		1.0 (0.49)	1.2 (0.80)
	Other (n=32)	1.4 (0.7, 2.5)	1.11 (0.50,	0.80		1.9 (0.26)	2.5 (0.05)		1.0 (0.33)	0.7 (0.03)		1.0 (0.33)	1.1 (0.40)
**Ethnicity: Hispanic**													
Overall (n=54)		34.8 (26.8, 43.8)				17.7 (<.001)	18.4 (<.001)		16.8 (<.001)	21.9 (<.001)		14.5 (<.001)	19.2 (<.001)
Purpose	Prevention (n=6)	22.6 (13.4 35.6)	0.51 (0.23,	0.08		17.7 (0.10)	17.6 (0.09)		16.8 (0.05)	22.2 (0.82)		14.5 (0.005)	19.5 (0.30)
	Treatment (n=48)	36.7 (27.6, 46.7)	Ref	Ref		17.7 (<.001)	20.7 (<.001)		16.8 (<.001)	21.2 (<.001)		14.5 (<.001)	18.5 (<.001)
Phase	Phase 1/ Phase 2	30.4 (21.0, 41.9)	Ref	Ref		17.7 (0.002)	21.3 (0.03)		16.8 (<.001)	24.4 (0.15)		14.5 (<.001)	21.6 (0.03)
	Phase 3/ Phase 4	37.9 (28.9, 47.8)	1.39 (0.73,	0.31		17.7 (<.001)	18.0 (<.001)		16.8 (<.001)	21.9 (<.001)		14.5 (<.001)	19.2 (<.001)
Primary Funder	Government (n=4)	28.7 (14.8, 48.2)	0.63 (0.24,	0.35		17.7 (0.03)	20.2 (0.10)		16.8 (0.02)	22.9 (0.26)		14.5 (0.004)	19.5 (0.08)
	Industry (n=23)	39.0 (28.0, 51.2)	Ref	Ref		17.7 (<.001)	18.1 (<.001)		16.8 (<.001)	22.1 (<.001)		14.5 (<.001)	19.4 (<.001)
	Other (n=27)	32.4 (19.4, 46.6)	0.73 (0.32,	0.42		17.7 (0.02)	16.1 (0.008)		16.8 (0.01)	17.0 (0.01)		14.5 (0.002)	14.7 (0.003)

© 2022 Xiao H et al. *JAMA Intern Med.*

eFigure. Forest plots of the study-level and summary estimates for each domain

Study or Subgroup	Events	Total	Weight (common)	Weight (random)	IV, Fixed + Random, 95% CI
prevention = 1					
O'Brien et al., 2021	814	1505	0.9%	1.2%	0.541 [0.515; 0.566]
Jackson et al., 2020	23	45	0.0%	0.8%	0.511 [0.358; 0.663]
Goepfert et al., 2021	229	439	0.3%	1.2%	0.522 [0.474; 0.569]
Chu et al., 2021	390	600	0.3%	1.2%	0.650 [0.610; 0.688]
Ali et al., 2021	1811	3726	2.2%	1.2%	0.486 [0.470; 0.502]
NCT04368260	20	40	0.0%	0.7%	0.500 [0.338; 0.662]
NCT04351243	71	225	0.1%	1.1%	0.316 [0.255; 0.381]
NCT04498247	148	263	0.2%	1.1%	0.563 [0.500; 0.624]
NCT04569786	118	232	0.1%	1.1%	0.509 [0.442; 0.575]
Falsey AR., 2021	14364	32379	19.1%	1.2%	0.444 [0.438; 0.449]
Shroff RT	22	70	0.0%	0.9%	0.314 [0.209; 0.436]
Dunkle LM., 2021	12271	25452	15.2%	1.2%	0.482 [0.476; 0.488]
Thomas SJ., 2021	21627	44047	26.3%	1.2%	0.491 [0.486; 0.496]
Polack FP., 2020	14366	30351	18.1%	1.2%	0.473 [0.468; 0.479]
Total (fixed effect, 95% CI)		139374	83.0%	--	0.476 [0.473; 0.478]
Total (random effects, 95% CI)			--	15.3%	0.489 [0.445; 0.534]
Heterogeneity: $Tau^2 = 0.1001$; $Chi^2 = 318.97$, df = 13 ($P < 0.01$); $I^2 = 96\%$					
prevention = 0					
Weinreich et al., 2021	2052	4057	2.4%	1.2%	0.506 [0.490; 0.521]
Uckun et al., 2021	39	76	0.0%	0.9%	0.513 [0.396; 0.630]
Thomas et al., 2021	132	214	0.1%	1.1%	0.617 [0.548; 0.682]
Taylor et al., 2021	26	80	0.0%	0.9%	0.325 [0.224; 0.439]
Strohbehn et al., 2021	16	32	0.0%	0.7%	0.500 [0.319; 0.681]
Skipper et al., 2020	238	423	0.2%	1.2%	0.563 [0.514; 0.611]
Sengupta et al., 2020	10	27	0.0%	0.6%	0.370 [0.194; 0.576]
Salazar et al., 2020	14	25	0.0%	0.6%	0.560 [0.349; 0.756]
Perepu et al., 2021	76	173	0.1%	1.1%	0.439 [0.364; 0.517]
Miller et al., 2020	16	30	0.0%	0.7%	0.533 [0.343; 0.717]
Lundgren et al., 2021	137	314	0.2%	1.1%	0.436 [0.381; 0.493]
Lanzoni et al., 2021	11	24	0.0%	0.6%	0.458 [0.256; 0.672]
Kaur et al., 2021	46	125	0.1%	1.0%	0.368 [0.284; 0.459]
Humeniuk et al., 2020	50	120	0.1%	1.0%	0.417 [0.327; 0.510]
Huang et al., 2021	123	294	0.2%	1.1%	0.418 [0.361; 0.477]
Hess et al., 2021	11	20	0.0%	0.5%	0.550 [0.315; 0.769]
Gupta et al., 2021	371	637	0.4%	1.2%	0.582 [0.543; 0.621]
Greenbaum et al., 2021	19	44	0.0%	0.8%	0.432 [0.283; 0.590]
Elamir et al., 2022	25	50	0.0%	0.8%	0.500 [0.355; 0.645]
Donato et al., 2021	26	51	0.0%	0.8%	0.510 [0.366; 0.652]
Dayya et al., 2021	50	136	0.1%	1.0%	0.368 [0.287; 0.455]
Bradfute et al., 2020	4	12	0.0%	0.4%	0.333 [0.099; 0.651]
Bennett−Guerrero et al., 2021	30	74	0.0%	0.9%	0.405 [0.293; 0.526]
Barnabas et al., 2021	277	689	0.4%	1.2%	0.402 [0.365; 0.440]
Bar et al., 2021	43	79	0.0%	0.9%	0.544 [0.428; 0.657]
Abella et al., 2021	91	132	0.1%	1.0%	0.689 [0.603; 0.767]
NCT04332081	7	80	0.0%	0.6%	0.088 [0.036; 0.172]
NCT04365699	15	81	0.0%	0.8%	0.185 [0.108; 0.287]
NCT04331899	50	120	0.1%	1.0%	0.417 [0.327; 0.510]
NCT04332107	174	260	0.1%	1.1%	0.669 [0.608; 0.726]
NCT04338009	68	152	0.1%	1.0%	0.447 [0.367; 0.530]
NCT04342663	109	152	0.1%	1.0%	0.717 [0.638; 0.787]
NCT04411667	13	33	0.0%	0.7%	0.394 [0.229; 0.579]
NCT04365153	12	45	0.0%	0.7%	0.267 [0.146; 0.419]
NCT04421404	19	34	0.0%	0.7%	0.559 [0.379; 0.728]
NCT04343261	21	48	0.0%	0.8%	0.438 [0.295; 0.588]
NCT04399980	14	40	0.0%	0.7%	0.350 [0.206; 0.517]
NCT04402970	11	30	0.0%	0.6%	0.367 [0.199; 0.561]
NCT04356937	102	243	0.1%	1.1%	0.420 [0.357; 0.485]
NCT04328467	760	1483	0.9%	1.2%	0.512 [0.487; 0.538]
NCT04335552	3	11	0.0%	0.3%	0.273 [0.060; 0.610]
NCT04340557	12	31	0.0%	0.7%	0.387 [0.218; 0.578]
NCT04401579	381	1033	0.6%	1.2%	0.369 [0.339; 0.399]
NCT04280705	378	1062	0.6%	1.2%	0.356 [0.327; 0.386]
NCT04380688	22	62	0.0%	0.8%	0.355 [0.237; 0.487]
NCT04315298	617	1861	1.0%	1.2%	0.332 [0.310; 0.353]
NCT04292899	1807	4838	2.7%	1.2%	0.374 [0.360; 0.387]
NCT04320615	132	438	0.2%	1.1%	0.301 [0.259; 0.347]
NCT04372186	154	377	0.2%	1.1%	0.408 [0.358; 0.460]
NCT04358068	11	16	0.0%	0.4%	0.688 [0.413; 0.890]
NCT04363736	40	97	0.1%	1.0%	0.412 [0.313; 0.517]
NCT04358081	7	19	0.0%	0.5%	0.368 [0.163; 0.616]
NCT04342897	35	95	0.1%	1.0%	0.368 [0.272; 0.474]
NCT04332991	212	479	0.3%	1.2%	0.443 [0.398; 0.488]
NCT04308668	690	1304	0.8%	1.2%	0.529 [0.502; 0.557]
NCT03852537	18	44	0.0%	0.8%	0.409 [0.263; 0.568]
NCT04425538	4	17	0.0%	0.4%	0.235 [0.068; 0.499]
NCT04456153	22	60	0.0%	0.8%	0.367 [0.246; 0.501]
NCT04435184	18	42	0.0%	0.8%	0.429 [0.277; 0.590]
NCT04347954	18	35	0.0%	0.7%	0.514 [0.340; 0.686]
NCT04701658	61	109	0.1%	1.0%	0.560 [0.461; 0.655]
NCT04348500	6	16	0.0%	0.5%	0.375 [0.152; 0.646]
NCT04377711	221	400	0.2%	1.2%	0.552 [0.502; 0.602]
NCT04524507	20	56	0.0%	0.8%	0.357 [0.234; 0.496]
NCT04354870	28	49	0.0%	0.8%	0.571 [0.422; 0.712]
NCT04359797	217	501	0.3%	1.2%	0.433 [0.389; 0.478]
NCT04343989	55	178	0.1%	1.0%	0.309 [0.242; 0.382]
NCT04502472	65	109	0.1%	1.0%	0.596 [0.498; 0.689]
NCT04871815	11	22	0.0%	0.6%	0.500 [0.282; 0.718]
NCT04706416	62	148	0.1%	1.0%	0.419 [0.338; 0.503]
NCT04432272	18	54	0.0%	0.8%	0.333 [0.211; 0.475]
NCT04685213	8	19	0.0%	0.5%	0.421 [0.203; 0.665]
NCT04425720	170	280	0.2%	1.1%	0.607 [0.547; 0.665]
NCT04374019	7	12	0.0%	0.4%	0.583 [0.277; 0.848]
NCT04365985	26	70	0.0%	0.9%	0.371 [0.259; 0.495]
NCT04363437	9	21	0.0%	0.5%	0.429 [0.218; 0.660]
NCT04353037	23	39	0.0%	0.7%	0.590 [0.421; 0.744]
NCT04425252	11	23	0.0%	0.6%	0.478 [0.268; 0.694]
NCT04401293	117	253	0.2%	1.1%	0.462 [0.400; 0.526]
NCT04504032	299	497	0.3%	1.2%	0.602 [0.557; 0.645]
NCT04492475	406	969	0.6%	1.2%	0.419 [0.388; 0.451]
NCT04377620	74	211	0.1%	1.1%	0.351 [0.286; 0.419]
NCT04583592	169	295	0.2%	1.1%	0.573 [0.514; 0.630]
NCT04411628	7	24	0.0%	0.5%	0.292 [0.126; 0.511]
NCT04501952	269	562	0.3%	1.2%	0.479 [0.437; 0.521]
NCT04498273	388	657	0.4%	1.2%	0.591 [0.552; 0.628]
NCT04411680	61	122	0.1%	1.0%	0.500 [0.408; 0.592]
NCT04317040	89	234	0.1%	1.1%	0.380 [0.318; 0.446]
NCT04405570	104	202	0.1%	1.1%	0.515 [0.444; 0.586]
NCT04355767	274	511	0.3%	1.2%	0.536 [0.492; 0.580]
NCT04409509	50	124	0.1%	1.0%	0.403 [0.316; 0.495]
NCT04494646	14	38	0.0%	0.7%	0.368 [0.218; 0.540]
NCT04349098	82	190	0.1%	1.1%	0.432 [0.360; 0.505]
NCT04388826	12	39	0.0%	0.7%	0.308 [0.170; 0.476]
NCT04357730	13	50	0.0%	0.7%	0.260 [0.146; 0.403]
Total (fixed effect, 95% CI)		29744	17.0%	--	0.450 [0.444; 0.456]
Total (random effects, 95% CI)			--	84.7%	0.446 [0.423; 0.470]
Heterogeneity: $Tau^2 = 0.1660$; $Chi^2 = 994.38$, df = 94 ($P < 0.01$); $I^2 = 91\%$					
**Total (fixed effect, 95% CI)**		**169118**	**100.0%**	**--**	**0.471 [0.469; 0.474]**
**Total (random effects, 95% CI)**			**--**	**100.0%**	**0.453 [0.432; 0.474]**

Heterogeneity: $Tau^2 = 0.1570$; $Chi^2 = 1375.86$, df = 108 ($P < 0.01$); $I^2 = 92\%$
Test for subgroup differences (fixed effect): $Chi^2 = 62.52$, df = 1 ($P < 0.01$)
Test for subgroup differences (random effects): $Chi^2 = 2.79$, df = 1 ($P = 0.10$)

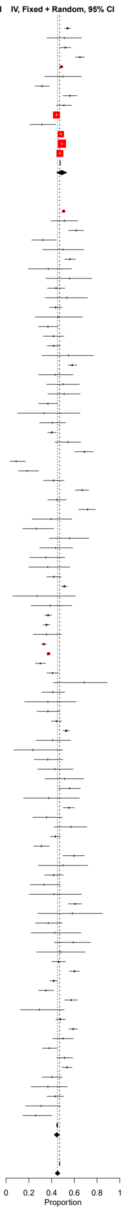

(A) Female

Study or Subgroup	Events	Total	Weight (common)	Weight (random)	IV, Fixed + Random, 95% CI
prevention = 1					
O'Brien et al., 2021	610	1497	1.2%	1.6%	0.407 [0.382; 0.433]
Jackson et al., 2020	6	45	0.0%	1.4%	0.133 [0.051; 0.268]
Goepfert et al., 2021	54	439	0.2%	1.5%	0.123 [0.094; 0.157]
Ali et al., 2021	432	3726	1.3%	1.6%	0.116 [0.106; 0.127]
NCT04351243	101	225	0.2%	1.5%	0.449 [0.383; 0.516]
NCT04498247	37	263	0.1%	1.5%	0.141 [0.101; 0.189]
NCT04569786	96	232	0.2%	1.5%	0.414 [0.350; 0.480]
Falsey AR., 2021	7223	31893	19.3%	1.6%	0.226 [0.222; 0.231]
Dunkle LM., 2021	5484	25401	14.9%	1.6%	0.216 [0.211; 0.221]
Thomas SJ., 2021	11399	43822	29.1%	1.6%	0.260 [0.256; 0.264]
Polack FP., 2020	6235	30070	17.1%	1.6%	0.207 [0.203; 0.212]
Total (fixed effect, 95% CI)		137613	83.6%	--	0.232 [0.230; 0.234]
Total (random effects, 95% CI)			--	16.8%	0.230 [0.167; 0.307]
Heterogeneity: $Tau^2 = 0.4262$; $Chi^2 = 986.85$, df = 10 ($P < 0.01$); $I^2 = 99\%$					
prevention = 0					
Weinreich et al., 2021	1542	3838	3.2%	1.6%	0.402 [0.386; 0.417]
Taylor et al., 2021	20	80	0.1%	1.5%	0.250 [0.160; 0.359]
Miller et al., 2020	3	30	0.0%	1.2%	0.100 [0.021; 0.265]
Lanzoni et al., 2021	22	24	0.0%	1.1%	0.917 [0.730; 0.990]
Humeniuk et al., 2020	117	120	0.0%	1.3%	0.975 [0.929; 0.995]
Hess et al., 2021	0	20	0.0%	0.7%	0.000 [0.000; 0.168]
Gupta et al., 2021	368	583	0.5%	1.5%	0.631 [0.591; 0.670]
Ghandehari et al., 2021	24	40	0.0%	1.5%	0.600 [0.433; 0.751]
Bradfute et al., 2020	0	12	0.0%	0.6%	0.000 [0.000; 0.265]
Barnabas et al., 2021	155	698	0.4%	1.5%	0.222 [0.192; 0.255]
Bar et al., 2021	3	79	0.0%	1.3%	0.038 [0.008; 0.107]
NCT04338009	82	152	0.1%	1.5%	0.539 [0.457; 0.621]
NCT04342663	5	146	0.0%	1.4%	0.034 [0.011; 0.078]
NCT04411667	28	33	0.0%	1.3%	0.848 [0.681; 0.949]
NCT04421404	23	33	0.0%	1.4%	0.697 [0.513; 0.844]
NCT04343261	12	46	0.0%	1.4%	0.261 [0.143; 0.411]
NCT04356937	109	228	0.2%	1.5%	0.478 [0.412; 0.545]
NCT04335552	6	11	0.0%	1.2%	0.545 [0.234; 0.833]
NCT04401579	531	1017	0.9%	1.6%	0.522 [0.491; 0.553]
NCT04280705	250	1005	0.6%	1.5%	0.249 [0.222; 0.277]
NCT04380688	28	61	0.1%	1.5%	0.459 [0.331; 0.592]
NCT04315298	526	1520	1.2%	1.6%	0.346 [0.322; 0.371]
NCT04292899	1754	4657	3.8%	1.6%	0.377 [0.363; 0.391]
NCT04320615	141	408	0.3%	1.5%	0.346 [0.299; 0.394]
NCT04372186	211	377	0.3%	1.5%	0.560 [0.508; 0.610]
NCT04358068	4	15	0.0%	1.3%	0.267 [0.078; 0.551]
NCT04363736	26	93	0.1%	1.5%	0.280 [0.191; 0.382]
NCT04342897	43	95	0.1%	1.5%	0.453 [0.350; 0.558]
NCT04308668	73	1290	0.2%	1.5%	0.057 [0.045; 0.071]
NCT04456153	42	60	0.0%	1.5%	0.700 [0.568; 0.812]
NCT04701658	50	105	0.1%	1.5%	0.476 [0.378; 0.576]
NCT04348500	8	16	0.0%	1.3%	0.500 [0.247; 0.753]
NCT04377711	172	400	0.3%	1.5%	0.430 [0.381; 0.480]
NCT04524507	18	55	0.0%	1.5%	0.327 [0.207; 0.467]
NCT04354870	2	49	0.0%	1.2%	0.041 [0.005; 0.140]
NCT04359797	417	483	0.2%	1.5%	0.863 [0.829; 0.893]
NCT04343989	49	178	0.1%	1.5%	0.275 [0.211; 0.347]
NCT04502472	31	105	0.1%	1.5%	0.295 [0.210; 0.392]
NCT04706416	131	148	0.1%	1.5%	0.885 [0.822; 0.932]
NCT04432272	2	54	0.0%	1.2%	0.037 [0.005; 0.127]
NCT04425720	179	280	0.2%	1.5%	0.639 [0.580; 0.696]
NCT04374019	0	13	0.0%	0.6%	0.000 [0.000; 0.247]
NCT04365985	2	62	0.0%	1.2%	0.032 [0.004; 0.112]
NCT04353037	3	39	0.0%	1.3%	0.077 [0.016; 0.209]
NCT04504032	95	493	0.3%	1.5%	0.193 [0.159; 0.230]
NCT04492475	311	951	0.7%	1.6%	0.327 [0.297; 0.358]
NCT04377620	54	205	0.1%	1.5%	0.263 [0.205; 0.329]
NCT04583592	177	294	0.2%	1.5%	0.602 [0.544; 0.658]
NCT04411628	15	24	0.0%	1.4%	0.625 [0.406; 0.812]
NCT04501952	235	539	0.5%	1.5%	0.436 [0.394; 0.479]
NCT04411680	35	121	0.1%	1.5%	0.289 [0.210; 0.379]
NCT04317040	85	234	0.2%	1.5%	0.363 [0.302; 0.428]
NCT04405570	86	201	0.2%	1.5%	0.428 [0.358; 0.499]
NCT04355767	156	505	0.4%	1.5%	0.309 [0.269; 0.351]
NCT04409509	24	118	0.1%	1.5%	0.203 [0.135; 0.287]
NCT04494646	11	38	0.0%	1.4%	0.289 [0.154; 0.459]
NCT04349098	73	185	0.2%	1.5%	0.395 [0.324; 0.469]
NCT04388826	14	39	0.0%	1.4%	0.359 [0.212; 0.528]
NCT04357730	32	48	0.0%	1.5%	0.667 [0.516; 0.796]
Total (fixed effect, 95% CI)		22753	16.4%	--	0.390 [0.383; 0.397]
Total (random effects, 95% CI)			--	83.2%	0.366 [0.291; 0.449]
Heterogeneity: $Tau^2 = 1.6577$; $Chi^2 = 1819.46$, df = 58 ($P = 0$); $I^2 = 97\%$					
**Total (fixed effect, 95% CI)**		**160366**	**100.0%**	**--**	**0.255 [0.253; 0.257]**
**Total (random effects, 95% CI)**			**--**	**100.0%**	**0.341 [0.278; 0.411]**

Heterogeneity: $Tau^2 = 1.4808$; $Chi^2 = 5026.01$, df = 69 ($P = 0$); $I^2 = 99\%$
Test for subgroup differences (fixed effect): $Chi^2 = 2219.70$, df = 1 ($P = 0$)
Test for subgroup differences (random effects): $Chi^2 = 6.10$, df = 1 ($P = 0.01$)

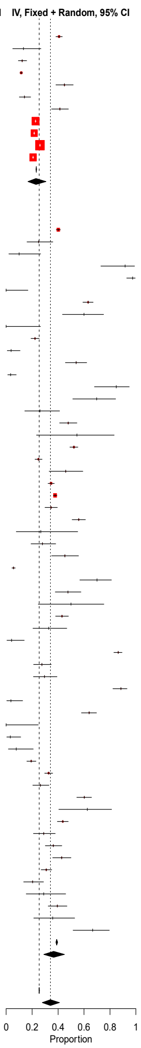

(B) Hispanic/ Latino

© 2022 Xiao H et al. *JAMA Intern Med.*

Study or Subgroup	Events	Total	Weight (common)	Weight (random)	IV, Fixed + Random, 95% CI
prevention = 1					
O'Brien et al., 2021	1288	1505	0.7%	1.5%	0.856 [0.837; 0.873]
Jackson et al., 2020	40	44	0.0%	1.1%	0.909 [0.783; 0.975]
Goepfert et al., 2021	381	439	0.2%	1.4%	0.868 [0.833; 0.898]
Chu et al., 2021	569	600	0.1%	1.4%	0.948 [0.927; 0.965]
Ali et al., 2021	3126	3697	1.9%	1.5%	0.846 [0.833; 0.857]
NCT04351243	113	172	0.2%	1.4%	0.657 [0.581; 0.728]
NCT04498247	254	263	0.0%	1.3%	0.966 [0.936; 0.984]
NCT04569786	197	232	0.1%	1.4%	0.849 [0.796; 0.893]
Falsey AR., 2021	25583	31825	20.1%	1.5%	0.804 [0.799; 0.808]
Dunkle LM., 2021	19324	25303	18.3%	1.5%	0.764 [0.758; 0.769]
Thomas SJ., 2021	36120	43796	25.3%	1.5%	0.825 [0.821; 0.828]
Polack FP., 2020	24024	28796	15.9%	1.5%	0.834 [0.830; 0.839]
Total (fixed effect, 95% CI)		136672	82.9%	--	0.811 [0.808; 0.813]
Total (random effects, 95% CI)			--	17.0%	0.857 [0.803; 0.899]
Heterogeneity: $Tau^2 = 0.4392$; $Chi^2 = 712.91$, df = 11 (P < 0.01); $I^2 = 98\%$					
prevention = 0					
Weinreich et al., 2021	3503	3953	1.6%	1.5%	0.886 [0.876; 0.896]
Uckun et al., 2021	47	76	0.1%	1.4%	0.618 [0.500; 0.728]
Thomas et al., 2021	152	214	0.2%	1.4%	0.710 [0.645; 0.770]
Taylor et al., 2021	28	80	0.1%	1.4%	0.350 [0.247; 0.465]
Miller et al., 2020	14	30	0.0%	1.3%	0.467 [0.283; 0.657]
Lanzoni et al., 2021	21	24	0.0%	1.0%	0.875 [0.676; 0.973]
Humeniuk et al., 2020	105	120	0.1%	1.3%	0.875 [0.802; 0.928]
Hess et al., 2021	5	20	0.0%	1.1%	0.250 [0.087; 0.491]
Gupta et al., 2021	506	581	0.3%	1.5%	0.871 [0.841; 0.897]
Greenbaum et al., 2021	19	19	0.0%	0.4%	1.000 [0.824; 1.000]
Ghandehari et al., 2021	31	40	0.0%	1.2%	0.775 [0.615; 0.892]
Bradfute et al., 2020	1	12	0.0%	0.6%	0.083 [0.002; 0.385]
Bennett−Guerrero et al., 2021	50	50	0.0%	0.4%	1.000 [0.929; 1.000]
Barnabas et al., 2021	369	689	0.7%	1.5%	0.536 [0.498; 0.573]
Bar et al., 2021	30	76	0.1%	1.4%	0.395 [0.284; 0.514]
NCT04342663	106	149	0.1%	1.4%	0.711 [0.632; 0.783]
NCT04421404	8	15	0.0%	1.1%	0.533 [0.266; 0.787]
NCT04343261	17	45	0.0%	1.3%	0.378 [0.238; 0.535]
NCT04356937	104	205	0.2%	1.4%	0.507 [0.437; 0.578]
NCT04335552	7	11	0.0%	1.0%	0.636 [0.308; 0.891]
NCT04401579	496	774	0.7%	1.5%	0.641 [0.606; 0.675]
NCT04280705	566	941	0.9%	1.5%	0.601 [0.569; 0.633]
NCT04380688	38	56	0.0%	1.3%	0.679 [0.540; 0.797]
NCT04315298	677	1152	1.1%	1.5%	0.588 [0.559; 0.616]
NCT04292899	2567	4598	4.5%	1.5%	0.558 [0.544; 0.573]
NCT04320615	252	378	0.3%	1.5%	0.667 [0.617; 0.714]
NCT04372186	199	346	0.3%	1.5%	0.575 [0.521; 0.628]
NCT04358068	14	15	0.0%	0.6%	0.933 [0.681; 0.998]
NCT04363736	38	80	0.1%	1.4%	0.475 [0.362; 0.590]
NCT04358081	7	12	0.0%	1.0%	0.583 [0.277; 0.848]
NCT04342897	74	95	0.1%	1.4%	0.779 [0.682; 0.858]
NCT04308668	742	1242	1.2%	1.5%	0.597 [0.570; 0.625]
NCT03852537	35	44	0.0%	1.2%	0.795 [0.647; 0.902]
NCT04456153	46	60	0.0%	1.3%	0.767 [0.640; 0.866]
NCT04435184	21	42	0.0%	1.3%	0.500 [0.342; 0.658]
NCT04701658	83	96	0.0%	1.3%	0.865 [0.780; 0.926]
NCT04348500	12	16	0.0%	1.0%	0.750 [0.476; 0.927]
NCT04377711	345	398	0.2%	1.4%	0.867 [0.829; 0.899]
NCT04524507	23	38	0.0%	1.3%	0.605 [0.434; 0.760]
NCT04354870	36	46	0.0%	1.3%	0.783 [0.636; 0.891]
NCT04359797	316	486	0.4%	1.5%	0.650 [0.606; 0.693]
NCT04343989	66	116	0.1%	1.4%	0.569 [0.474; 0.661]
NCT04502472	65	79	0.0%	1.3%	0.823 [0.721; 0.900]
NCT04432272	44	48	0.0%	1.1%	0.917 [0.800; 0.977]
NCT04685213	3	19	0.0%	1.0%	0.158 [0.034; 0.396]
NCT04374019	13	13	0.0%	0.4%	1.000 [0.753; 1.000]
NCT04365985	45	67	0.1%	1.4%	0.672 [0.546; 0.782]
NCT04353037	25	35	0.0%	1.2%	0.714 [0.537; 0.854]
NCT04425252	15	23	0.0%	1.2%	0.652 [0.427; 0.836]
NCT04401293	102	197	0.2%	1.4%	0.518 [0.446; 0.589]
NCT04504032	441	496	0.2%	1.4%	0.889 [0.858; 0.915]
NCT04492475	584	852	0.7%	1.5%	0.685 [0.653; 0.717]
NCT04377620	149	184	0.1%	1.4%	0.810 [0.746; 0.864]
NCT04583592	233	289	0.2%	1.4%	0.806 [0.756; 0.850]
NCT04411628	19	22	0.0%	1.0%	0.864 [0.651; 0.971]
NCT04501952	452	549	0.3%	1.5%	0.823 [0.789; 0.854]
NCT04498273	494	588	0.3%	1.5%	0.840 [0.808; 0.869]
NCT04411680	81	93	0.0%	1.3%	0.871 [0.785; 0.932]
NCT04317040	111	167	0.1%	1.4%	0.665 [0.588; 0.736]
NCT04405570	176	202	0.1%	1.4%	0.871 [0.817; 0.914]
NCT04355767	337	511	0.5%	1.5%	0.659 [0.617; 0.701]
NCT04409509	93	112	0.1%	1.4%	0.830 [0.748; 0.895]
NCT04494646	25	32	0.0%	1.2%	0.781 [0.600; 0.907]
NCT04349098	78	170	0.2%	1.4%	0.459 [0.382; 0.537]
NCT04388826	35	38	0.0%	1.0%	0.921 [0.786; 0.983]
NCT04357730	30	35	0.0%	1.1%	0.857 [0.697; 0.952]
Total (fixed effect, 95% CI)		22261	17.1%	--	0.666 [0.659; 0.672]
Total (random effects, 95% CI)			--	83.0%	0.707 [0.661; 0.749]
Heterogeneity: $Tau^2 = 0.6471$; $Chi^2 = 1932.6$, df = 65 (P = 0); $I^2 = 97\%$					
**Total (fixed effect, 95% CI)**		**158933**	**100.0%**	**--**	**0.790 [0.788; 0.792]**
**Total (random effects, 95% CI)**			**--**	**100.0%**	**0.739 [0.697; 0.777]**

Heterogeneity: $Tau^2 = 0.7467$; $Chi^2 = 4717.83$, df = 77 (P = 0); $I^2 = 98\%$
Test for subgroup differences (fixed effect): $Chi^2 = 2072.33$, df = 1 (P = 0)
Test for subgroup differences (random effects): $Chi^2 = 16.28$, df = 1 (P < 0.01)

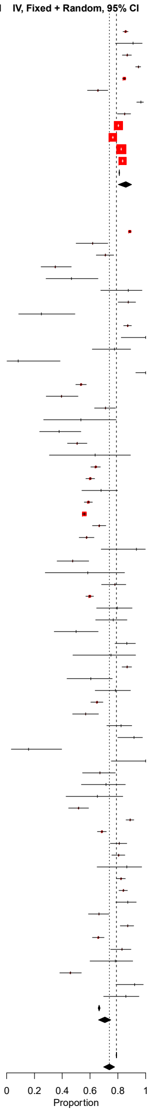

(C)White

Study or Subgroup	Events	Total	Weight (common)	Weight (random)	IV, Fixed + Random, 95% CI
prevention = 1					
O'Brien et al., 2021	140	1505	0.9%	1.5%	0.093 [0.079; 0.109]
Jackson et al., 2020	2	44	0.0%	0.9%	0.045 [0.006; 0.155]
Goepfert et al., 2021	17	439	0.1%	1.4%	0.039 [0.023; 0.061]
Chu et al., 2021	16	600	0.1%	1.4%	0.027 [0.015; 0.043]
Ali et al., 2021	125	3697	0.8%	1.5%	0.034 [0.028; 0.040]
NCT04351243	45	172	0.2%	1.5%	0.262 [0.198; 0.334]
NCT04498247	5	263	0.0%	1.2%	0.019 [0.006; 0.044]
NCT04569786	27	232	0.2%	1.5%	0.116 [0.078; 0.165]
Falsey AR., 2021	2686	31825	16.9%	1.5%	0.084 [0.081; 0.088]
Dunkle LM., 2021	2798	25303	17.1%	1.5%	0.111 [0.107; 0.115]
Thomas SJ., 2021	4216	43796	26.2%	1.5%	0.096 [0.094; 0.099]
Polack FP., 2020	3090	28796	19.0%	1.5%	0.107 [0.104; 0.111]
Total (fixed effect, 95% CI)		136672	81.7%	--	0.098 [0.096; 0.100]
Total (random effects, 95% CI)			--	17.2%	0.072 [0.047; 0.109]
Heterogeneity: $Tau^2 = 0.5932$; $Chi^2 = 405.62$, df = 11 (P < 0.01); $I^2 = 97\%$					
prevention = 0					
Weinreich et al., 2021	209	3953	1.4%	1.5%	0.053 [0.046; 0.060]
Uckun et al., 2021	28	76	0.1%	1.4%	0.368 [0.261; 0.487]
Thomas et al., 2021	51	214	0.3%	1.5%	0.238 [0.183; 0.301]
Taylor et al., 2021	44	80	0.1%	1.5%	0.550 [0.435; 0.662]
Miller et al., 2020	12	30	0.0%	1.3%	0.400 [0.227; 0.594]
Lanzoni et al., 2021	3	24	0.0%	1.1%	0.125 [0.027; 0.324]
Humeniuk et al., 2020	15	120	0.1%	1.4%	0.125 [0.072; 0.198]
Hess et al., 2021	15	20	0.0%	1.2%	0.750 [0.509; 0.913]
Gupta et al., 2021	38	581	0.2%	1.5%	0.065 [0.047; 0.089]
Greenbaum et al., 2021	0	19	0.0%	0.4%	0.000 [0.000; 0.176]
Ghandehari et al., 2021	4	40	0.0%	1.2%	0.100 [0.028; 0.237]
Bradfute et al., 2020	0	12	0.0%	0.4%	0.000 [0.000; 0.265]
Bennett−Guerrero et al., 2021	0	50	0.0%	0.4%	0.000 [0.000; 0.071]
Barnabas et al., 2021	54	689	0.3%	1.5%	0.078 [0.059; 0.101]
Bar et al., 2021	42	76	0.1%	1.4%	0.553 [0.434; 0.667]
NCT04342663	38	149	0.2%	1.5%	0.255 [0.187; 0.333]
NCT04421404	2	15	0.0%	0.9%	0.133 [0.017; 0.405]
NCT04343261	16	45	0.1%	1.4%	0.356 [0.219; 0.512]
NCT04356937	40	205	0.2%	1.5%	0.195 [0.143; 0.256]
NCT04335552	3	11	0.0%	1.0%	0.273 [0.060; 0.610]
NCT04401579	156	774	0.9%	1.5%	0.202 [0.174; 0.232]
NCT04280705	226	941	1.2%	1.5%	0.240 [0.213; 0.269]
NCT04380688	12	56	0.1%	1.4%	0.214 [0.116; 0.344]
NCT04315298	350	1152	1.7%	1.5%	0.304 [0.277; 0.331]
NCT04292899	804	4598	4.6%	1.5%	0.175 [0.164; 0.186]
NCT04320615	66	378	0.4%	1.5%	0.175 [0.138; 0.217]
NCT04372186	57	346	0.3%	1.5%	0.165 [0.127; 0.208]
NCT04358068	1	15	0.0%	0.7%	0.067 [0.002; 0.319]
NCT04363736	33	80	0.1%	1.5%	0.412 [0.304; 0.528]
NCT04358081	3	12	0.0%	1.0%	0.250 [0.055; 0.572]
NCT04342897	15	95	0.1%	1.4%	0.158 [0.091; 0.247]
NCT04308668	52	1242	0.3%	1.5%	0.042 [0.031; 0.055]
NCT03852537	2	44	0.0%	0.9%	0.045 [0.006; 0.155]
NCT04456153	8	60	0.0%	1.3%	0.133 [0.059; 0.246]
NCT04435184	20	42	0.1%	1.4%	0.476 [0.320; 0.636]
NCT04701658	6	96	0.0%	1.3%	0.062 [0.023; 0.131]
NCT04348500	2	16	0.0%	0.9%	0.125 [0.016; 0.383]
NCT04377711	47	398	0.3%	1.5%	0.118 [0.088; 0.154]
NCT04524507	14	38	0.1%	1.4%	0.368 [0.218; 0.540]
NCT04354870	0	46	0.0%	0.4%	0.000 [0.000; 0.077]
NCT04359797	99	486	0.5%	1.5%	0.204 [0.169; 0.242]
NCT04343989	32	116	0.2%	1.5%	0.276 [0.197; 0.367]
NCT04502472	4	79	0.0%	1.2%	0.051 [0.014; 0.125]
NCT04432272	4	48	0.0%	1.2%	0.083 [0.023; 0.200]
NCT04685213	5	19	0.0%	1.2%	0.263 [0.091; 0.512]
NCT04374019	0	13	0.0%	0.4%	0.000 [0.000; 0.247]
NCT04365985	14	67	0.1%	1.4%	0.209 [0.119; 0.326]
NCT04353037	3	35	0.0%	1.1%	0.086 [0.018; 0.231]
NCT04425252	4	23	0.0%	1.1%	0.174 [0.050; 0.388]
NCT04401293	70	197	0.3%	1.5%	0.355 [0.289; 0.426]
NCT04504032	36	496	0.2%	1.5%	0.073 [0.051; 0.099]
NCT04492475	160	852	0.9%	1.5%	0.188 [0.162; 0.216]
NCT04377620	26	184	0.2%	1.5%	0.141 [0.094; 0.200]
NCT04583592	46	289	0.3%	1.5%	0.159 [0.119; 0.207]
NCT04411628	2	22	0.0%	0.9%	0.091 [0.011; 0.292]
NCT04501952	42	549	0.3%	1.5%	0.077 [0.056; 0.102]
NCT04498273	78	588	0.5%	1.5%	0.133 [0.106; 0.163]
NCT04411680	7	93	0.0%	1.3%	0.075 [0.031; 0.149]
NCT04317040	50	167	0.2%	1.5%	0.299 [0.231; 0.375]
NCT04405570	11	202	0.1%	1.4%	0.054 [0.027; 0.095]
NCT04355767	103	511	0.6%	1.5%	0.202 [0.168; 0.239]
NCT04409509	13	112	0.1%	1.4%	0.116 [0.063; 0.190]
NCT04494646	4	32	0.0%	1.1%	0.125 [0.035; 0.290]
NCT04349098	47	170	0.2%	1.5%	0.276 [0.211; 0.350]
NCT04388826	1	38	0.0%	0.7%	0.026 [0.001; 0.138]
NCT04357730	1	35	0.0%	0.7%	0.029 [0.001; 0.149]
Total (fixed effect, 95% CI)		22261	18.3%	--	0.174 [0.169; 0.180]
Total (random effects, 95% CI)			--	82.8%	0.165 [0.136; 0.199]
Heterogeneity: $Tau^2 = 0.7130$; $Chi^2 = 1227.47$, df = 65 (P < 0.01); $I^2 = 95\%$					
**Total (fixed effect, 95% CI)**		**158933**	**100.0%**	**--**	**0.109 [0.108; 0.111]**
**Total (random effects, 95% CI)**			**--**	**100.0%**	**0.143 [0.118; 0.172]**

Heterogeneity: $Tau^2 = 0.8326$; $Chi^2 = 2591.43$, df = 77 (P = 0); $I^2 = 97\%$
Test for subgroup differences (fixed effect): $Chi^2 = 958.34$, df = 1 (P < 0.01)
Test for subgroup differences (random effects): $Chi^2 = 12.85$, df = 1 (P < 0.01)

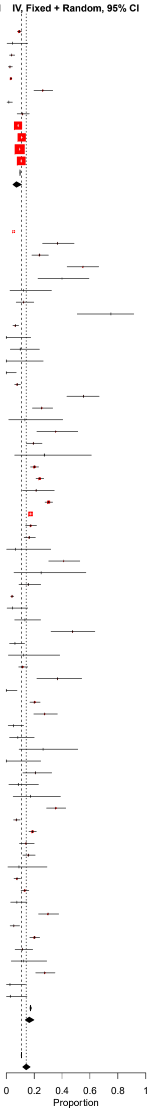

(D) Black

© 2022 Xiao H et al. *JAMA Intern Med.*

Study or Subgroup	Events	Total	Weight (common)	Weight (random)	IV, Fixed + Random, 95% CI
prevention = 1					
O'Brien et al., 2021	42	1505	0.5%	1.9%	0.028 [0.020; 0.038]
Jackson et al., 2020	1	44	0.0%	0.8%	0.023 [0.001; 0.120]
Goepfert et al., 2021	28	439	0.4%	1.9%	0.064 [0.043; 0.091]
Chu et al., 2021	7	600	0.1%	1.7%	0.012 [0.005; 0.024]
Ali et al., 2021	221	3697	2.8%	2.0%	0.060 [0.052; 0.068]
NCT04351243	11	172	0.1%	1.8%	0.064 [0.032; 0.112]
NCT04498247	2	263	0.0%	1.2%	0.008 [0.001; 0.027]
NCT04569786	4	232	0.1%	1.5%	0.017 [0.005; 0.044]
Falsey AR., 2021	1428	31825	18.3%	2.0%	0.045 [0.043; 0.047]
Dunkle LM., 2021	1127	25303	14.5%	2.0%	0.045 [0.042; 0.047]
Thomas SJ., 2021	1894	43796	24.4%	2.0%	0.043 [0.041; 0.045]
Polack FP., 2020	1382	28796	17.7%	2.0%	0.048 [0.046; 0.051]
Total (fixed effect, 95% CI)		136672	78.9%	--	0.045 [0.044; 0.046]
Total (random effects, 95% CI)			--	20.6%	0.038 [0.029; 0.051]
Heterogeneity: $Tau^2 = 0.2012$; $Chi^2 = 66.84$, df = 11 ($P < 0.01$); $I^2 = 84\%$					
prevention = 0					
Weinreich et al., 2021	182	3953	2.3%	2.0%	0.046 [0.040; 0.053]
Uckun et al., 2021	0	76	0.0%	0.5%	0.000 [0.000; 0.047]
Thomas et al., 2021	0	214	0.0%	0.5%	0.000 [0.000; 0.017]
Taylor et al., 2021	0	80	0.0%	0.5%	0.000 [0.000; 0.045]
Miller et al., 2020	2	30	0.0%	1.1%	0.067 [0.008; 0.221]
Lanzoni et al., 2021	0	24	0.0%	0.5%	0.000 [0.000; 0.142]
Humeniuk et al., 2020	0	120	0.0%	0.5%	0.000 [0.000; 0.030]
Hess et al., 2021	0	20	0.0%	0.5%	0.000 [0.000; 0.168]
Gupta et al., 2021	34	581	0.4%	1.9%	0.059 [0.041; 0.081]
Greenbaum et al., 2021	0	19	0.0%	0.5%	0.000 [0.000; 0.176]
Ghandehari et al., 2021	2	40	0.0%	1.1%	0.050 [0.006; 0.169]
Bradfute et al., 2020	0	12	0.0%	0.5%	0.000 [0.000; 0.265]
Bennett−Guerrero et al., 2021	0	50	0.0%	0.5%	0.000 [0.000; 0.071]
Barnabas et al., 2021	73	689	0.9%	1.9%	0.106 [0.084; 0.131]
Bar et al., 2021	4	76	0.1%	1.5%	0.053 [0.015; 0.129]
NCT04342663	4	149	0.1%	1.5%	0.027 [0.007; 0.067]
NCT04421404	4	15	0.0%	1.3%	0.267 [0.078; 0.551]
NCT04343261	0	45	0.0%	0.5%	0.000 [0.000; 0.079]
NCT04356937	9	205	0.1%	1.7%	0.044 [0.020; 0.082]
NCT04335552	0	11	0.0%	0.5%	0.000 [0.000; 0.285]
NCT04401579	101	774	1.2%	2.0%	0.130 [0.108; 0.156]
NCT04280705	135	941	1.6%	2.0%	0.143 [0.122; 0.168]
NCT04380688	0	56	0.0%	0.5%	0.000 [0.000; 0.064]
NCT04315298	113	1152	1.4%	2.0%	0.098 [0.082; 0.117]
NCT04292899	446	4598	5.4%	2.0%	0.097 [0.089; 0.106]
NCT04320615	38	378	0.5%	1.9%	0.101 [0.072; 0.135]
NCT04372186	6	346	0.1%	1.6%	0.017 [0.006; 0.037]
NCT04358068	0	15	0.0%	0.5%	0.000 [0.000; 0.218]
NCT04363736	4	80	0.1%	1.5%	0.050 [0.014; 0.123]
NCT04358081	1	12	0.0%	0.8%	0.083 [0.002; 0.385]
NCT04342897	4	95	0.1%	1.5%	0.042 [0.012; 0.104]
NCT04308668	410	1242	3.7%	2.0%	0.330 [0.304; 0.357]
NCT03852537	2	44	0.0%	1.1%	0.045 [0.006; 0.155]
NCT04456153	0	60	0.0%	0.5%	0.000 [0.000; 0.060]
NCT04435184	1	42	0.0%	0.8%	0.024 [0.001; 0.126]
NCT04701658	2	96	0.0%	1.2%	0.021 [0.003; 0.073]
NCT04348500	2	16	0.0%	1.1%	0.125 [0.016; 0.383]
NCT04377711	2	398	0.0%	1.2%	0.005 [0.001; 0.018]
NCT04524507	0	38	0.0%	0.5%	0.000 [0.000; 0.093]
NCT04354870	10	46	0.1%	1.7%	0.217 [0.109; 0.364]
NCT04359797	14	486	0.2%	1.8%	0.029 [0.016; 0.048]
NCT04343989	18	116	0.2%	1.8%	0.155 [0.095; 0.234]
NCT04502472	10	79	0.1%	1.7%	0.127 [0.062; 0.220]
NCT04432272	0	48	0.0%	0.5%	0.000 [0.000; 0.074]
NCT04685213	1	19	0.0%	0.8%	0.053 [0.001; 0.260]
NCT04374019	0	13	0.0%	0.5%	0.000 [0.000; 0.247]
NCT04365985	0	67	0.0%	0.5%	0.000 [0.000; 0.054]
NCT04353037	5	35	0.1%	1.5%	0.143 [0.048; 0.303]
NCT04425252	0	23	0.0%	0.5%	0.000 [0.000; 0.148]
NCT04401293	25	197	0.3%	1.9%	0.127 [0.084; 0.182]
NCT04504032	1	496	0.0%	0.8%	0.002 [0.000; 0.011]
NCT04492475	83	852	1.0%	2.0%	0.097 [0.078; 0.119]
NCT04377620	7	184	0.1%	1.6%	0.038 [0.015; 0.077]
NCT04583592	5	289	0.1%	1.5%	0.017 [0.006; 0.040]
NCT04411628	1	22	0.0%	0.8%	0.045 [0.001; 0.228]
NCT04501952	13	549	0.2%	1.8%	0.024 [0.013; 0.040]
NCT04498273	9	588	0.1%	1.7%	0.015 [0.007; 0.029]
NCT04411680	2	93	0.0%	1.2%	0.022 [0.003; 0.076]
NCT04317040	4	167	0.1%	1.5%	0.024 [0.007; 0.060]
NCT04405570	6	202	0.1%	1.6%	0.030 [0.011; 0.064]
NCT04355767	18	511	0.2%	1.8%	0.035 [0.021; 0.055]
NCT04409509	4	112	0.1%	1.5%	0.036 [0.010; 0.089]
NCT04494646	2	32	0.0%	1.1%	0.062 [0.008; 0.208]
NCT04349098	10	170	0.1%	1.7%	0.059 [0.029; 0.106]
NCT04388826	0	38	0.0%	0.5%	0.000 [0.000; 0.093]
NCT04357730	0	35	0.0%	0.5%	0.000 [0.000; 0.100]
Total (fixed effect, 95% CI)		22261	21.1%	--	0.108 [0.104; 0.113]
Total (random effects, 95% CI)			--	79.4%	0.046 [0.036; 0.060]
Heterogeneity: $Tau^2 = 0.8157$; $Chi^2 = 1079.96$, df = 65 ($P < 0.01$); $I^2 = 94\%$					
**Total (fixed effect, 95% CI)**		**158933**	**100.0%**	**--**	**0.055 [0.054; 0.056]**
**Total (random effects, 95% CI)**			**--**	**100.0%**	**0.044 [0.035; 0.055]**

Heterogeneity: $Tau^2 = 0.7171$; $Chi^2 = 2246.85$, df = 77 ($P = 0$); $I^2 = 97\%$
Test for subgroup differences (fixed effect): $Chi^2 = 1100.05$, df = 1 ($P < 0.01$)
Test for subgroup differences (random effects): $Chi^2 = 0.89$, df = 1 ($P = 0.35$)

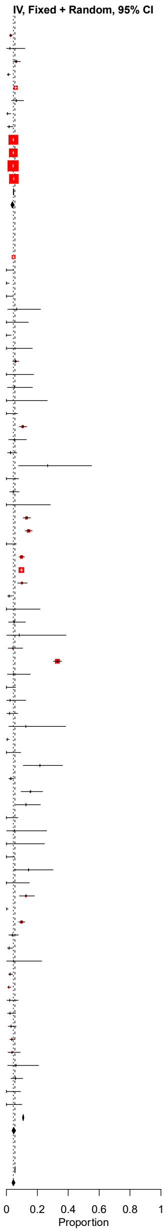

(E)Asian

Study or Subgroup	Events	Total	Weight (common)	Weight (random)	IV, Fixed + Random, 95% CI
prevention = 1					
O'Brien et al., 2021	3	1505	0.7%	2.2%	0.002 [0.000; 0.006]
Jackson et al., 2020	0	44	0.1%	0.7%	0.000 [0.000; 0.080]
Goepfert et al., 2021	1	439	0.2%	1.2%	0.002 [0.000; 0.013]
Chu et al., 2021	1	600	0.2%	1.2%	0.002 [0.000; 0.009]
Ali et al., 2021	2	3697	0.5%	1.9%	0.001 [0.000; 0.002]
NCT04351243	0	172	0.1%	0.8%	0.000 [0.000; 0.021]
NCT04498247	0	263	0.1%	0.8%	0.000 [0.000; 0.014]
NCT04569786	0	232	0.1%	0.8%	0.000 [0.000; 0.016]
Falsey AR., 2021	82	31825	18.7%	3.5%	0.003 [0.002; 0.003]
Dunkle LM., 2021	57	25303	13.0%	3.5%	0.002 [0.002; 0.003]
Thomas SJ., 2021	90	43796	20.6%	3.5%	0.002 [0.002; 0.003]
Polack FP., 2020	67	28796	15.3%	3.5%	0.002 [0.002; 0.003]
Total (fixed effect, 95% CI)		136672	69.8%	--	0.002 [0.002; 0.003]
Total (random effects, 95% CI)			--	23.7%	0.002 [0.002; 0.003]
Heterogeneity: $Tau^2 = 0$; $Chi^2 = 7.83$, df = 11 ($P = 0.73$); $I^2 = 0\%$					
prevention = 0					
Weinreich et al., 2021	0	3953	0.1%	0.8%	0.000 [0.000; 0.001]
Uckun et al., 2021	1	76	0.2%	1.2%	0.013 [0.000; 0.071]
Thomas et al., 2021	0	214	0.1%	0.8%	0.000 [0.000; 0.017]
Taylor et al., 2021	0	80	0.1%	0.7%	0.000 [0.000; 0.045]
Miller et al., 2020	0	30	0.1%	0.7%	0.000 [0.000; 0.116]
Lanzoni et al., 2021	0	24	0.1%	0.7%	0.000 [0.000; 0.142]
Humeniuk et al., 2020	0	120	0.1%	0.7%	0.000 [0.000; 0.030]
Hess et al., 2021	0	20	0.1%	0.7%	0.000 [0.000; 0.168]
Gupta et al., 2021	0	581	0.1%	0.8%	0.000 [0.000; 0.006]
Greenbaum et al., 2021	0	19	0.1%	0.7%	0.000 [0.000; 0.176]
Ghandehari et al., 2021	0	40	0.1%	0.7%	0.000 [0.000; 0.088]
Bradfute et al., 2020	0	12	0.1%	0.7%	0.000 [0.000; 0.265]
Bennett−Guerrero et al., 2021	0	50	0.1%	0.7%	0.000 [0.000; 0.071]
Barnabas et al., 2021	2	689	0.5%	1.9%	0.003 [0.000; 0.010]
Bar et al., 2021	0	76	0.1%	0.7%	0.000 [0.000; 0.047]
NCT04342663	0	149	0.1%	0.8%	0.000 [0.000; 0.024]
NCT04421404	0	15	0.1%	0.7%	0.000 [0.000; 0.218]
NCT04343261	0	45	0.1%	0.7%	0.000 [0.000; 0.079]
NCT04356937	1	205	0.2%	1.2%	0.005 [0.000; 0.027]
NCT04335552	0	11	0.1%	0.7%	0.000 [0.000; 0.285]
NCT04401579	11	774	2.5%	3.1%	0.014 [0.007; 0.025]
NCT04280705	4	941	0.9%	2.4%	0.004 [0.001; 0.011]
NCT04380688	0	56	0.1%	0.7%	0.000 [0.000; 0.064]
NCT04315298	6	1152	1.4%	2.7%	0.005 [0.002; 0.011]
NCT04292899	41	4598	9.3%	3.5%	0.009 [0.006; 0.012]
NCT04320615	8	378	1.8%	2.9%	0.021 [0.009; 0.041]
NCT04372186	1	346	0.2%	1.2%	0.003 [0.000; 0.016]
NCT04358068	0	15	0.1%	0.7%	0.000 [0.000; 0.218]
NCT04363736	1	80	0.2%	1.2%	0.013 [0.000; 0.068]
NCT04358081	1	12	0.2%	1.2%	0.083 [0.002; 0.385]
NCT04342897	1	95	0.2%	1.2%	0.011 [0.000; 0.057]
NCT04308668	9	1242	2.0%	3.0%	0.007 [0.003; 0.014]
NCT03852537	0	44	0.1%	0.7%	0.000 [0.000; 0.080]
NCT04456153	0	60	0.1%	0.7%	0.000 [0.000; 0.060]
NCT04435184	0	42	0.1%	0.7%	0.000 [0.000; 0.084]
NCT04701658	1	96	0.2%	1.2%	0.010 [0.000; 0.057]
NCT04348500	0	16	0.1%	0.7%	0.000 [0.000; 0.206]
NCT04377711	3	398	0.7%	2.2%	0.008 [0.002; 0.022]
NCT04524507	0	38	0.1%	0.7%	0.000 [0.000; 0.093]
NCT04354870	0	46	0.1%	0.7%	0.000 [0.000; 0.077]
NCT04359797	0	486	0.1%	0.8%	0.000 [0.000; 0.008]
NCT04343989	0	116	0.1%	0.7%	0.000 [0.000; 0.031]
NCT04502472	0	79	0.1%	0.7%	0.000 [0.000; 0.046]
NCT04432272	0	48	0.1%	0.7%	0.000 [0.000; 0.074]
NCT04685213	0	19	0.1%	0.7%	0.000 [0.000; 0.176]
NCT04374019	0	13	0.1%	0.7%	0.000 [0.000; 0.247]
NCT04365985	0	67	0.1%	0.7%	0.000 [0.000; 0.054]
NCT04353037	2	35	0.4%	1.8%	0.057 [0.007; 0.192]
NCT04425252	0	23	0.1%	0.7%	0.000 [0.000; 0.148]
NCT04401293	0	197	0.1%	0.8%	0.000 [0.000; 0.019]
NCT04504032	0	496	0.1%	0.8%	0.000 [0.000; 0.007]
NCT04492475	9	852	2.0%	3.0%	0.011 [0.005; 0.020]
NCT04377620	0	184	0.1%	0.8%	0.000 [0.000; 0.020]
NCT04583592	0	289	0.1%	0.8%	0.000 [0.000; 0.013]
NCT04411628	0	22	0.1%	0.7%	0.000 [0.000; 0.154]
NCT04501952	1	549	0.2%	1.2%	0.002 [0.000; 0.010]
NCT04498273	4	588	0.9%	2.4%	0.007 [0.002; 0.017]
NCT04411680	1	93	0.2%	1.2%	0.011 [0.000; 0.058]
NCT04317040	0	167	0.1%	0.8%	0.000 [0.000; 0.022]
NCT04405570	0	202	0.1%	0.8%	0.000 [0.000; 0.018]
NCT04355767	0	511	0.1%	0.8%	0.000 [0.000; 0.007]
NCT04409509	1	112	0.2%	1.2%	0.009 [0.000; 0.049]
NCT04494646	0	32	0.1%	0.7%	0.000 [0.000; 0.109]
NCT04349098	2	170	0.5%	1.8%	0.012 [0.001; 0.042]
NCT04388826	1	38	0.2%	1.2%	0.026 [0.001; 0.138]
NCT04357730	0	35	0.1%	0.7%	0.000 [0.000; 0.100]
Total (fixed effect, 95% CI)		22261	30.2%	--	0.009 [0.008; 0.011]
Total (random effects, 95% CI)			--	76.3%	0.009 [0.007; 0.011]
Heterogeneity: $Tau^2 = 0.0868$; $Chi^2 = 67.9$, df = 65 ($P = 0.38$); $I^2 = 4\%$					
**Total (fixed effect, 95% CI)**		**158933**	**100.0%**	**--**	**0.003 [0.003; 0.004]**
**Total (random effects, 95% CI)**			**--**	**100.0%**	**0.006 [0.005; 0.008]**

Heterogeneity: $Tau^2 = 0.5241$; $Chi^2 = 255.29$, df = 77 ($P < 0.01$); $I^2 = 70\%$
Test for subgroup differences (fixed effect): $Chi^2 = 179.55$, df = 1 ($P < 0.01$)
Test for subgroup differences (random effects): $Chi^2 = 115.62$, df = 1 ($P < 0.01$)

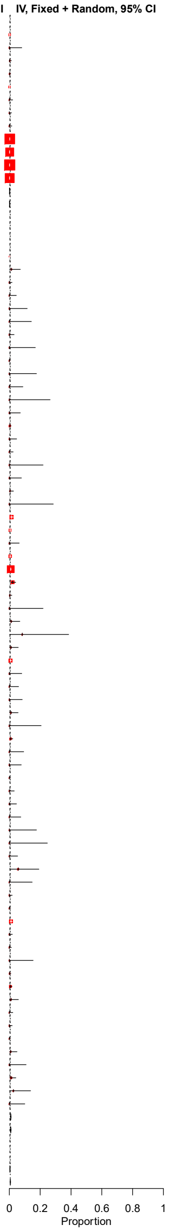

(F) Native Hawaiian/Pacific Islander

© 2022 Xiao H et al. *JAMA Intern Med.*

Study or Subgroup	Events	Total	Weight (common)	Weight (random)	IV, Fixed + Random, 95% CI
prevention = 1					
O'Brien et al., 2021	3	1505	0.7%	2.2%	0.002 [0.000; 0.006]
Jackson et al., 2020	0	44	0.1%	0.7%	0.000 [0.000; 0.080]
Goepfert et al., 2021	1	439	0.2%	1.2%	0.002 [0.000; 0.013]
Chu et al., 2021	1	600	0.2%	1.2%	0.002 [0.000; 0.009]
Ali et al., 2021	2	3697	0.5%	1.9%	0.001 [0.000; 0.002]
NCT04351243	0	172	0.1%	0.8%	0.000 [0.000; 0.021]
NCT04498247	0	263	0.1%	0.8%	0.000 [0.000; 0.014]
NCT04569786	0	232	0.1%	0.8%	0.000 [0.000; 0.016]
Falsey AR., 2021	82	31825	18.7%	3.5%	0.003 [0.002; 0.003]
Dunkle LM., 2021	57	25303	13.0%	3.5%	0.002 [0.002; 0.003]
Thomas SJ., 2021	90	43796	20.6%	3.5%	0.002 [0.002; 0.003]
Polack FP., 2020	67	28796	15.3%	3.5%	0.002 [0.002; 0.003]
**Total (fixed effect, 95% CI)**		**136672**	**69.8%**	**--**	**0.002 [0.002; 0.003]**
**Total (random effects, 95% CI)**			**--**	**23.7%**	**0.002 [0.002; 0.003]**
Heterogeneity: $Tau^2 = 0$; $Chi^2 = 7.83$, df = 11 (P = 0.73); $I^2 = 0\%$					
prevention = 0					
Weinreich et al., 2021	0	3953	0.1%	0.8%	0.000 [0.000; 0.001]
Uckun et al., 2021	1	76	0.2%	1.2%	0.013 [0.000; 0.071]
Thomas et al., 2021	0	214	0.1%	0.8%	0.000 [0.000; 0.017]
Taylor et al., 2021	0	80	0.1%	0.7%	0.000 [0.000; 0.045]
Miller et al., 2020	0	30	0.1%	0.7%	0.000 [0.000; 0.116]
Lanzoni et al., 2021	0	24	0.1%	0.7%	0.000 [0.000; 0.142]
Humeniuk et al., 2020	0	120	0.1%	0.7%	0.000 [0.000; 0.030]
Hess et al., 2021	0	20	0.1%	0.7%	0.000 [0.000; 0.168]
Gupta et al., 2021	0	581	0.1%	0.8%	0.000 [0.000; 0.006]
Greenbaum et al., 2021	0	19	0.1%	0.7%	0.000 [0.000; 0.176]
Ghandehari et al., 2021	0	40	0.1%	0.7%	0.000 [0.000; 0.088]
Bradfute et al., 2020	0	12	0.1%	0.7%	0.000 [0.000; 0.265]
Bennett−Guerrero et al., 2021	0	50	0.1%	0.7%	0.000 [0.000; 0.071]
Barnabas et al., 2021	2	689	0.5%	1.9%	0.003 [0.000; 0.010]
Bar et al., 2021	0	76	0.1%	0.7%	0.000 [0.000; 0.047]
NCT04342663	0	149	0.1%	0.8%	0.000 [0.000; 0.024]
NCT04421404	0	15	0.1%	0.7%	0.000 [0.000; 0.218]
NCT04343261	0	45	0.1%	0.7%	0.000 [0.000; 0.079]
NCT04356937	1	205	0.2%	1.2%	0.005 [0.000; 0.027]
NCT04335552	0	11	0.1%	0.7%	0.000 [0.000; 0.285]
NCT04401579	11	774	2.5%	3.1%	0.014 [0.007; 0.025]
NCT04280705	4	941	0.9%	2.4%	0.004 [0.001; 0.011]
NCT04380688	0	56	0.1%	0.7%	0.000 [0.000; 0.064]
NCT04315298	6	1152	1.4%	2.7%	0.005 [0.002; 0.011]
NCT04292899	41	4598	9.3%	3.5%	0.009 [0.006; 0.012]
NCT04320615	8	378	1.8%	2.9%	0.021 [0.009; 0.041]
NCT04372186	1	346	0.2%	1.2%	0.003 [0.000; 0.016]
NCT04358068	0	15	0.1%	0.7%	0.000 [0.000; 0.218]
NCT04363736	1	80	0.2%	1.2%	0.013 [0.000; 0.068]
NCT04358081	1	12	0.2%	1.2%	0.083 [0.002; 0.385]
NCT04342897	1	95	0.2%	1.2%	0.011 [0.000; 0.057]
NCT04308668	9	1242	2.0%	3.0%	0.007 [0.003; 0.014]
NCT03852537	0	44	0.1%	0.7%	0.000 [0.000; 0.080]
NCT04456153	0	60	0.1%	0.7%	0.000 [0.000; 0.060]
NCT04435184	0	42	0.1%	0.7%	0.000 [0.000; 0.084]
NCT04701658	1	96	0.2%	1.2%	0.010 [0.000; 0.057]
NCT04348500	0	16	0.1%	0.7%	0.000 [0.000; 0.206]
NCT04377711	3	398	0.7%	2.2%	0.008 [0.002; 0.022]
NCT04524507	0	38	0.1%	0.7%	0.000 [0.000; 0.093]
NCT04354870	0	46	0.1%	0.7%	0.000 [0.000; 0.077]
NCT04359797	0	486	0.1%	0.8%	0.000 [0.000; 0.008]
NCT04343989	0	116	0.1%	0.7%	0.000 [0.000; 0.031]
NCT04502472	0	79	0.1%	0.7%	0.000 [0.000; 0.046]
NCT04432272	0	48	0.1%	0.7%	0.000 [0.000; 0.074]
NCT04685213	0	19	0.1%	0.7%	0.000 [0.000; 0.176]
NCT04374019	0	13	0.1%	0.7%	0.000 [0.000; 0.247]
NCT04365985	0	67	0.1%	0.7%	0.000 [0.000; 0.054]
NCT04353037	2	35	0.4%	1.8%	0.057 [0.007; 0.192]
NCT04425252	0	23	0.1%	0.7%	0.000 [0.000; 0.148]
NCT04401293	0	197	0.1%	0.8%	0.000 [0.000; 0.019]
NCT04504032	0	496	0.1%	0.8%	0.000 [0.000; 0.007]
NCT04492475	9	852	2.0%	3.0%	0.011 [0.005; 0.020]
NCT04377620	0	184	0.1%	0.8%	0.000 [0.000; 0.020]
NCT04583592	0	289	0.1%	0.8%	0.000 [0.000; 0.013]
NCT04411628	0	22	0.1%	0.7%	0.000 [0.000; 0.154]
NCT04501952	1	549	0.2%	1.2%	0.002 [0.000; 0.010]
NCT04498273	4	588	0.9%	2.4%	0.007 [0.002; 0.017]
NCT04411680	1	93	0.2%	1.2%	0.011 [0.000; 0.058]
NCT04317040	0	167	0.1%	0.8%	0.000 [0.000; 0.022]
NCT04405570	0	202	0.1%	0.8%	0.000 [0.000; 0.018]
NCT04355767	0	511	0.1%	0.8%	0.000 [0.000; 0.007]
NCT04409509	1	112	0.2%	1.2%	0.009 [0.000; 0.049]
NCT04494646	0	32	0.1%	0.7%	0.000 [0.000; 0.109]
NCT04349098	2	170	0.5%	1.8%	0.012 [0.001; 0.042]
NCT04388826	1	38	0.2%	1.2%	0.026 [0.001; 0.138]
NCT04357730	0	35	0.1%	0.7%	0.000 [0.000; 0.100]
**Total (fixed effect, 95% CI)**		**22261**	**30.2%**	**--**	**0.009 [0.008; 0.011]**
**Total (random effects, 95% CI)**			**--**	**76.3%**	**0.009 [0.007; 0.011]**
Heterogeneity: $Tau^2 = 0.0868$; $Chi^2 = 67.9$, df = 65 (P = 0.38); $I^2 = 4\%$					
**Total (fixed effect, 95% CI)**		**158933**	**100.0%**	**--**	**0.003 [0.003; 0.004]**
**Total (random effects, 95% CI)**			**--**	**100.0%**	**0.006 [0.005; 0.008]**

Heterogeneity: $Tau^2 = 0.5241$; $Chi^2 = 255.29$, df = 77 (P < 0.01); $I^2 = 70\%$
Test for subgroup differences (fixed effect): $Chi^2 = 179.55$, df = 1 (P < 0.01)
Test for subgroup differences (random effects): $Chi^2 = 115.62$, df = 1 (P < 0.01)

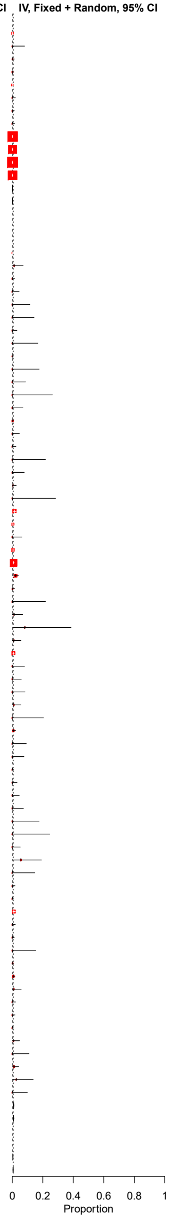

(G) American Indian/Native Alaska

© 2022 Xiao H et al. *JAMA Intern Med.*

eReferences

1. Abella BS, Jolkovsky EL, Biney BT, et al. Efficacy and Safety of Hydroxychloroquine vs Placebo for Pre-exposure SARS-CoV-2 Prophylaxis Among Health Care Workers: A Randomized Clinical Trial. *JAMA internal medicine*. Feb 1 2021;181(2):195-202. doi:10.1001/jamainternmed.2020.6319
2. Ali K, Berman G, Zhou H, et al. Evaluation of mRNA-1273 SARS-CoV-2 Vaccine in Adolescents. *New England Journal of Medicine*. 2021/12/09 2021;385(24):2241-2251. doi:10.1056/NEJMoa2109522
3. Bar KJ, Shaw PA, Choi GH, et al. A randomized controlled study of convalescent plasma for individuals hospitalized with COVID-19 pneumonia. *The Journal of clinical investigation*. Dec 15 2021;131(24)doi:10.1172/jci155114
4. Barnabas RV, Brown ER, Bershteyn A, et al. Hydroxychloroquine as Postexposure Prophylaxis to Prevent Severe Acute Respiratory Syndrome Coronavirus 2 Infection : A Randomized Trial. *Annals of internal medicine*. Mar 2021;174(3):344-352. doi:10.7326/m20-6519
5. Bennett-Guerrero E, Romeiser JL, Talbot LR, et al. Severe Acute Respiratory Syndrome Coronavirus 2 Convalescent Plasma Versus Standard Plasma in Coronavirus Disease 2019 Infected Hospitalized Patients in New York: A Double-Blind Randomized Trial. *Critical care medicine*. Jul 1 2021;49(7):1015-1025. doi:10.1097/ccm.0000000000005066
6. Bikdeli B, Talasaz AH, Rashidi F, et al. Intermediate-Dose versus Standard-Dose Prophylactic Anticoagulation in Patients with COVID-19 Admitted to the Intensive Care Unit: 90-Day Results from the INSPIRATION Randomized Trial. *Thrombosis and haemostasis*. Jan 2022;122(1):131-141. doi:10.1055/a-1485-2372
7. Bradfute SB, Hurwitz I, Yingling AV, et al. Severe Acute Respiratory Syndrome Coronavirus 2 Neutralizing Antibody Titers in Convalescent Plasma and Recipients in New Mexico: An Open Treatment Study in Patients With Coronavirus Disease 2019. *The Journal of infectious diseases*. Oct 13 2020;222(10):1620-1628. doi:10.1093/infdis/jiaa505
8. Chigutsa E, O'Brien L, Ferguson-Sells L, Long A, Chien J. Population Pharmacokinetics and Pharmacodynamics of the Neutralizing Antibodies Bamlanivimab and Etesevimab in Patients With Mild to Moderate COVID-19 Infection. *Clin Pharmacol Ther*. Nov 2021;110(5):1302-1310. doi:10.1002/cpt.2420
9. Chu L, McPhee R, Huang W, et al. A preliminary report of a randomized controlled phase 2 trial of the safety and immunogenicity of mRNA-1273 SARS-CoV-2 vaccine. *Vaccine*. May 12 2021;39(20):2791-2799. doi:10.1016/j.vaccine.2021.02.007
10. Dayya D, O'Neill OJ, Feiertag TD, et al. The use of oxygen hoods in patients failing on conventional high-flow oxygen delivery systems, the effects on oxygenation, mechanical ventilation and mortality rates in hypoxic patients with COVID-19. A Prospective Controlled Cohort Study. *Respiratory medicine*. Apr 2021;179:106312. doi:10.1016/j.rmed.2021.106312
11. Donato ML, Park S, Baker M, et al. Clinical and laboratory evaluation of patients with SARS-CoV-2 pneumonia treated with high-titer convalescent plasma. *JCI insight*. Mar 22 2021;6(6)doi:10.1172/jci.insight.143196
12. Dunkle LM, Kotloff KL, Gay CL, et al. Efficacy and safety of NVX-CoV2373 in adults in the United States and Mexico. 2022;386(6):531-543.
13. Elamir YM, Amir H, Lim S, et al. A randomized pilot study using calcitriol in hospitalized COVID-19 patients. *Bone*. Jan 2022;154:116175. doi:10.1016/j.bone.2021.116175
14. Falsey AR, Sobieszczyk ME, Hirsch I, et al. Phase 3 safety and efficacy of AZD1222 (ChAdOx1 nCoV-19) Covid-19 vaccine. 2021;385(25):2348-2360.
15. Ghandehari S, Matusov Y, Pepkowitz S, et al. Progesterone in Addition to Standard of Care vs Standard of Care Alone in the Treatment of Men Hospitalized With Moderate to Severe COVID-19: A Randomized, Controlled Pilot Trial. *Chest*. Jul 2021;160(1):74-84. doi:10.1016/j.chest.2021.02.024
16. Goepfert PA, Fu B, Chabanon A-L, et al. Safety and immunogenicity of SARS-CoV-2 recombinant protein vaccine formulations in healthy adults: a randomised, placebo-controlled, dose-ranging study. 2021:2021.01.19.20248611. doi:10.1101/2021.01.19.20248611 %J medRxiv
17. Greenbaum U, Klein K, Martinez F, et al. High levels of common cold coronavirus antibodies in convalescent plasma are associated with improved survival in COVID-19 patients. *medRxiv : the preprint server for health sciences*. Mar 10 2021;doi:10.1101/2021.03.08.21252775
18. Gupta A, Gonzalez-Rojas Y, Juarez E, et al. Early Treatment for Covid-19 with SARS-CoV-2 Neutralizing Antibody Sotrovimab. *The New England journal of medicine*. Nov 18 2021;385(21):1941-1950. doi:10.1056/NEJMoa2107934

© 2022 Xiao H et al. *JAMA Intern Med.*

19. Hess CB, Nasti TH, Dhere VR, et al. Immunomodulatory Low-Dose Whole-Lung Radiation for Patients with Coronavirus Disease 2019-Related Pneumonia. *International journal of radiation oncology, biology, physics*. Mar 15 2021;109(4):867-879. doi:10.1016/j.ijrobp.2020.12.011
20. Huang YH, Huang JT. Use of chlorhexidine to eradicate oropharyngeal SARS-CoV-2 in COVID-19 patients. *Journal of medical virology*. Jul 2021;93(7):4370-4373. doi:10.1002/jmv.26954
21. Humeniuk R, Mathias A, Cao H, et al. Safety, Tolerability, and Pharmacokinetics of Remdesivir, An Antiviral for Treatment of COVID-19, in Healthy Subjects. *Clinical and translational science*. Sep 2020;13(5):896-906. doi:10.1111/cts.12840
22. Jackson LA, Anderson EJ, Rouphael NG, et al. An mRNA Vaccine against SARS-CoV-2 - Preliminary Report. *The New England journal of medicine*. Nov 12 2020;383(20):1920-1931. doi:10.1056/NEJMoa2022483
23. Kaur R, Vines DL, Mirza S, et al. Early versus late awake prone positioning in non-intubated patients with COVID-19.
24. Kimura KS, Freeman MH, Wessinger BC, et al. Interim analysis of an open-label randomized controlled trial evaluating nasal irrigations in non-hospitalized patients with coronavirus disease 2019. *International forum of allergy & rhinology*. Dec 2020;10(12):1325-1328. doi:10.1002/alr.22703
25. Lanzoni G, Linetsky E, Correa D, et al. Umbilical cord mesenchymal stem cells for COVID-19 acute respiratory distress syndrome: A double-blind, phase 1/2a, randomized controlled trial. *Stem cells translational medicine*. May 2021;10(5):660-673. doi:10.1002/sctm.20-0472
26. Lerner D, Garvey K, Arrighi-Allisan A, et al. Letter to the editor: Study Summary - Randomized Control Trial of Omega-3 Fatty Acid Supplementation for the Treatment of COVID-19 Related Olfactory Dysfunction. *Trials*. Nov 23 2020;21(1):942. doi:10.1186/s13063-020-04905-y
27. Liu HH, Ezekowitz MD, Columbo M, et al. The future is now: our experience starting a remote clinical trial during the beginning of the COVID-19 pandemic. *Trials*. 2021/09/07 2021;22(1):603. doi:10.1186/s13063-021-05537-6
28. Lundgren JD, Grund B, Barkauskas CE, et al. A Neutralizing Monoclonal Antibody for Hospitalized Patients with Covid-19. *The New England journal of medicine*. Mar 11 2021;384(10):905-914. doi:10.1056/NEJMoa2033130
29. Miller J, Bruen C, Schnaus M, et al. Auxora versus standard of care for the treatment of severe or critical COVID-19 pneumonia: results from a randomized controlled trial. *Critical care (London, England)*. Aug 14 2020;24(1):502. doi:10.1186/s13054-020-03220-x
30. Nickel RS, Margulies S, Frazer B, Luban NLC, Webb J. Combination dose-escalated hydroxyurea and transfusion: an approach to conserve blood during the COVID-19 pandemic. *Blood*. Jun 18 2020;135(25):2320-2322. doi:10.1182/blood.2020006582
31. O'Brien MP, Forleo-Neto E, Musser BJ, et al. Subcutaneous REGEN-COV Antibody Combination for Covid-19 Prevention. *medRxiv : the preprint server for health sciences*. Jun 17 2021;doi:10.1101/2021.06.14.21258567
32. Perepu US, Chambers I, Wahab A, et al. Standard prophylactic versus intermediate dose enoxaparin in adults with severe COVID-19: A multi-center, open-label, randomized controlled trial. *Journal of thrombosis and haemostasis : JTH*. Sep 2021;19(9):2225-2234. doi:10.1111/jth.15450
33. Baden LR, El Sahly HM, Essink B, et al. Efficacy and Safety of the mRNA-1273 SARS-CoV-2 Vaccine. *New England Journal of Medicine*. 2021/02/04 2020;384(5):403-416. doi:10.1056/NEJMoa2035389
34. Salazar E, Perez KK, Ashraf M, et al. Treatment of Coronavirus Disease 2019 (COVID-19) Patients with Convalescent Plasma. *The American journal of pathology*. Aug 2020;190(8):1680-1690. doi:10.1016/j.ajpath.2020.05.014
35. Sengupta V, Sengupta S, Lazo A, Woods P, Nolan A, Bremer N. Exosomes Derived from Bone Marrow Mesenchymal Stem Cells as Treatment for Severe COVID-19. *Stem cells and development*. Jun 15 2020;29(12):747-754. doi:10.1089/scd.2020.0080
36. Shah T, McCarthy M, Nasir I, et al. Design and rationale of the colchicine/statin for the prevention of COVID-19 complications (COLSTAT) trial. *Contemporary clinical trials*. Nov 2021;110:106547. doi:10.1016/j.cct.2021.106547
37. Shroff RT, Chalasani P, Wei R, et al. Immune responses to two and three doses of the BNT162b2 mRNA vaccine in adults with solid tumors. *Nature Medicine*. 2021/11/01 2021;27(11):2002-2011. doi:10.1038/s41591-021-01542-z
38. Skipper CP, Pastick KA, Engen NW, et al. Hydroxychloroquine in Nonhospitalized Adults With Early COVID-19 : A Randomized Trial. *Annals of internal medicine*. Oct 20 2020;173(8):623-631. doi:10.7326/m20-4207

© 2022 Xiao H et al. *JAMA Intern Med.*

39. Strohbehn GW, Heiss BL, Rouhani SJ, et al. COVIDOSE: A Phase II Clinical Trial of Low-Dose Tocilizumab in the Treatment of Noncritical COVID-19 Pneumonia. *Clinical pharmacology and therapeutics*. Mar 2021;109(3):688-696. doi:10.1002/cpt.2117
40. Taylor SP, Bundy H, Smith WM, Skavroneck S, Taylor B, Kowalkowski MA. Awake Prone Positioning Strategy for Nonintubated Hypoxic Patients with COVID-19: A Pilot Trial with Embedded Implementation Evaluation. *Annals of the American Thoracic Society*. Aug 2021;18(8):1360-1368. doi:10.1513/AnnalsATS.202009-1164OC
41. Thomas S, Patel D, Bittel B, et al. Effect of High-Dose Zinc and Ascorbic Acid Supplementation vs Usual Care on Symptom Length and Reduction Among Ambulatory Patients With SARS-CoV-2 Infection: The COVID A to Z Randomized Clinical Trial. *JAMA network open*. Feb 1 2021;4(2):e210369. doi:10.1001/jamanetworkopen.2021.0369
42. Thomas SJ, Moreira ED, Jr., Kitchin N, et al. Safety and Efficacy of the BNT162b2 mRNA Covid-19 Vaccine through 6 Months. *The New England journal of medicine*. Nov 4 2021;385(19):1761-1773. doi:10.1056/NEJMoa2110345
43. Uckun FM, Orhan C, Powell J, et al. Non-clinical safety profile and pharmacodynamics of two formulations of the anti-sepsis drug candidate Rejuveinix (RJX). *Biomedicine & pharmacotherapy = Biomedecine & pharmacotherapie*. Sep 2021;141:111823. doi:10.1016/j.biopha.2021.111823
44. Wang R, DeGruttola V, Lei Q, et al. The vitamin D for COVID-19 (VIVID) trial: A pragmatic cluster-randomized design. *Contemporary clinical trials*. Jan 2021;100:106176. doi:10.1016/j.cct.2020.106176
45. Weinreich DM, Sivapalasingam S, Norton T, et al. REGEN-COV Antibody Combination and Outcomes in Outpatients with Covid-19. *The New England journal of medicine*. Dec 2 2021;385(23):e81. doi:10.1056/NEJMoa2108163
46. Steroid Dosing by bioMARker Guided Titration in Critically Ill Patients With Pneumonia. Clinicaltrials.gov. Updated September 16, 2021. Accessed September 18, 2022. https://clinicaltrials.gov/ct2/show/NCT03852537
47. Adaptive COVID-19 Treatment Trial (ACTT). ClinicalTrials.gov. Updated March 14, 2022. Accessed September 17, 2022. https://ClinicalTrials.gov/show/NCT04280705
48. Study to Evaluate the Safety and Antiviral Activity of Remdesivir (GS-5734,Ñ¢) in Participants With Severe Coronavirus Disease (COVID-19). ClinicalTrials.gov. Updated December 31, 2020. Accessed September 17, 2022. https://ClinicalTrials.gov/show/NCT04292899
49. Post-exposure Prophylaxis / Preemptive Therapy for SARS-Coronavirus-2. ClinicalTrials.gov. Updated May 13, 2021. Accessed September 17, 2022. https://ClinicalTrials.gov/show/NCT04308668
50. Evaluation of the Efficacy and Safety of Sarilumab in Hospitalized Patients With COVID-19. ClinicalTrials.gov. Updated September 23, 2021. Accessed September 17, 2022. https://ClinicalTrials.gov/show/NCT04315298
51. CD24Fc (MK-7110) as a Non-antiviral Immunomodulator in COVID-19 Treatment (MK-7110-007). ClinicalTrials.gov. Updated October 15, 2021. Accessed September 17, 2022. https://ClinicalTrials.gov/show/NCT04317040
52. A Study to Evaluate the Safety and Efficacy of Tocilizumab in Patients With Severe COVID-19 Pneumonia. ClinicalTrials.gov. Updated June 30, 2021. Accessed September 17, 2022. https://ClinicalTrials.gov/show/NCT04320615
53. Pre-exposure Prophylaxis for SARS-Coronavirus-2. ClinicalTrials.gov. Updated July 2, 2021. Accessed September 17, 2022. https://ClinicalTrials.gov/show/NCT04328467
54. Single-Blind Study of a Single Dose of Peginterferon Lambda-1a Compared With Placebo in Outpatients With Mild COVID-19. ClinicalTrials.gov. Updated November 30, 2021. Accessed September 17, 2022. https://ClinicalTrials.gov/show/NCT04331899
55. Hyperbaric Oxygen for COVID-19 Patients. ClinicalTrials.gov. Updated June 22, 2021. Accessed September 17, 2022. https://ClinicalTrials.gov/show/NCT04332081
56. Azithromycin for COVID-19 Treatment in Outpatients Nationwide. ClinicalTrials.gov. Updated October 7, 2021. Accessed September 17, 2022. https://ClinicalTrials.gov/show/NCT04332107
57. Outcomes Related to COVID-19 Treated With Hydroxychloroquine Among In-patients With Symptomatic Disease. ClinicalTrials.gov. Updated March 17, 2021. Accessed September 17, 2022. https://ClinicalTrials.gov/show/NCT04332991

© 2022 Xiao H et al. *JAMA Intern Med.*

58. Pragmatic Factorial Trial of Hydroxychloroquine, Azithromycin, or Both for Treatment of Severe SARS-CoV-2 Infection. ClinicalTrials.gov. Updated May 17, 2021. Accessed September 17, 2022. https://ClinicalTrials.gov/show/NCT04335552
59. Elimination or Prolongation of ACE Inhibitors and ARB in Coronavirus Disease 2019. ClinicalTrials.gov. Updated April 9, 2021. Accessed September 17, 2022. https://ClinicalTrials.gov/show/NCT04338009
60. Do Angiotensin Receptor Blockers Mitigate Progression to Acute Respiratory Distress Syndrome With SARS-CoV-2 Infection. ClinicalTrials.gov. Updated May 26, 2021. Accessed September 17, 2022. https://ClinicalTrials.gov/show/NCT04340557
61. A Double-blind, Placebo-controlled Clinical Trial of Fluvoxamine for Symptomatic Individuals With COVID-19 Infection. ClinicalTrials.gov. Updated July 9, 2021. Accessed September 17, 2022. https://ClinicalTrials.gov/show/NCT04342663
62. A Study of LY3127804 in Participants With COVID-19. ClinicalTrials.gov. Updated August 12, 2021. Accessed September 17, 2022. https://ClinicalTrials.gov/show/NCT04342897
63. Convalescent Plasma in the Treatment of COVID 19. ClinicalTrials.gov. Updated September 24, 2020. Accessed September 17, 2022. https://ClinicalTrials.gov/show/NCT04343261
64. A Randomized Placebo-controlled Safety and Dose-finding Study for the Use of the IL-6 Inhibitor Clazakizumab in Patients With Life-threatening COVID-19 Infection. ClinicalTrials.gov. Updated June 14, 2022. Accessed September 17, 2022. https://ClinicalTrials.gov/show/NCT04343989
65. PVP-I Nasal Sprays and SARS-CoV-2 Nasopharyngeal Titers (for COVID-19). ClinicalTrials.gov. Updated October 21, 2021. Accessed September 17, 2022. https://ClinicalTrials.gov/show/NCT04347954
66. Clazakizumab (Anti-Interleukin 6 (IL-6) Monoclonal) Compared to Placebo for Coronavirus Disease 2019 (COVID-19). ClinicalTrials.gov. Updated December 2, 2021. Accessed September 17, 2022. https://ClinicalTrials.gov/show/NCT04348500
67. Evaluation of Activity and Safety of Oral Selinexor in Participants With Severe COVID-19 Infection. ClinicalTrials.gov. Updated November 1, 2021. Accessed September 17, 2022. https://ClinicalTrials.gov/show/NCT04349098
68. A Study to Assess the Efficacy and Safety of Gimsilumab in Subjects With Lung Injury or Acute Respiratory Distress Syndrome Secondary to COVID-19 (BREATHE). ClinicalTrials.gov. Updated December 14, 2021. Accessed September 17, 2022. https://ClinicalTrials.gov/show/NCT04351243
69. PATCH 2&3:Prevention & Treatment of COVID-19 (Severe Acute Respiratory Syndrome Coronavirus 2) With Hydroxychloroquine. ClinicalTrials.gov. Updated October 19, 2021. Accessed September 17, 2022. https://ClinicalTrials.gov/show/NCT04353037
70. COVID-19 PrEP HCW HCQ Study. ClinicalTrials.gov. Updated October 6, 2021. Accessed September 17, 2022. https://ClinicalTrials.gov/show/NCT04354870
71. Convalescent Plasma in Outpatients With COVID-19. ClinicalTrials.gov. Updated October 19, 2021. Accessed September 17, 2022. https://ClinicalTrials.gov/show/NCT04355767
72. Efficacy of Tocilizumab on Patients With COVID-19. ClinicalTrials.gov. Updated July 27, 2021. Accessed September 17, 2022. https://ClinicalTrials.gov/show/NCT04356937
73. Fibrinolytic Therapy to Treat ARDS in the Setting of COVID-19 Infection. ClinicalTrials.gov. Updated January 20, 2022. Accessed September 17, 2022. https://ClinicalTrials.gov/show/NCT04357730
74. Evaluating the Efficacy of Hydroxychloroquine and Azithromycin to Prevent Hospitalization or Death in Persons With COVID-19. ClinicalTrials.gov. Updated November 16, 2021. Accessed September 17, 2022. https://ClinicalTrials.gov/show/NCT04358068
75. Hydroxychloroquine Monotherapy and in Combination With Azithromycin in Patients With Moderate and Severe COVID-19 Disease. ClinicalTrials.gov. Updated October 11, 2021. Accessed September 17, 2022. https://ClinicalTrials.gov/show/NCT04358081
76. COVID-19 Patient Positioning Pragmatic Trial. ClinicalTrials.gov. Updated December 27, 2021. Accessed September 17, 2022. https://ClinicalTrials.gov/show/NCT04359797
77. COlchicine in Moderate-severe Hospitalized Patients Before ARDS to Treat COVID-19. ClinicalTrials.gov. Updated February 18, 2022. Accessed September 17, 2022. https://ClinicalTrials.gov/show/NCT04363437

© 2022 Xiao H et al. *JAMA Intern Med.*

78. A Study to Investigate Intravenous Tocilizumab in Participants With Moderate to Severe COVID-19 Pneumonia. ClinicalTrials.gov. Updated August 31, 2022. Accessed September 17, 2022. https://ClinicalTrials.gov/show/NCT04363736
79. Canakinumab in Covid-19 Cardiac Injury (The Three C Study). ClinicalTrials.gov. Updated April 15, 2021. Accessed September 17, 2022. https://ClinicalTrials.gov/show/NCT04365153
80. Cardiovascular Effects of COVID-19. ClinicalTrials.gov. Updated September 16, 2021. Accessed September 17, 2022. https://ClinicalTrials.gov/show/NCT04365699
81. Study of Immunomodulation Using Naltrexone and Ketamine for COVID-19. ClinicalTrials.gov. Updated January 26, 2022. Accessed September 17, 2022. https://ClinicalTrials.gov/show/NCT04365985
82. Clinical Validation of New Injection Molded Flocked Nasopharyngeal Swabs in Response to the COVID-19 Pandemic. ClinicalTrials.gov. Updated February 17, 2022. Accessed September 17, 2022. https://ClinicalTrials.gov/show/NCT04368260
83. A Study to Evaluate the Efficacy and Safety of Tocilizumab in Hospitalized Participants With COVID-19 Pneumonia. ClinicalTrials.gov. Updated September 27, 2021. Accessed September 17, 2022. https://ClinicalTrials.gov/show/NCT04372186
84. Novel Agents for Treatment of High-risk COVID-19 Positive Patients. ClinicalTrials.gov. Updated January 20, 2022. Accessed September 17, 2022. https://ClinicalTrials.gov/show/NCT04374019
85. Assessment of Efficacy and Safety of Ruxolitinib in Participants With COVID-19-Associated ARDS Who Require Mechanical Ventilation (RUXCOVID-DEVENT). ClinicalTrials.gov. Updated January 19, 2022. Accessed September 17, 2022. https://ClinicalTrials.gov/show/NCT04377620
86. A Study of the Safety and Efficacy of Ciclesonide in the Treatment of Non-hospitalized COVID-19 Patients. ClinicalTrials.gov. Updated January 11, 2022. Accessed September 17, 2022. https://ClinicalTrials.gov/show/NCT04377711
87. Acalabrutinib Study With Best Supportive Care Versus Best Supportive Care in Subjects Hospitalized With COVID-19. ClinicalTrials.gov. Updated September 13, 2021. Accessed September 17, 2022. https://ClinicalTrials.gov/show/NCT04380688
88. COVID-19 Treatment of Severe Acute Respiratory Syndrome With Veru-111. ClinicalTrials.gov. Updated November 24, 2021. Accessed September 17, 2022. https://ClinicalTrials.gov/show/NCT04388826
89. Mavrilimumab to Reduce Progression of Acute Respiratory Failure in COVID-19 Pneumonia and Systemic Hyper-inflammation. ClinicalTrials.gov. Updated May 13, 2021. Accessed September 17, 2022. https://ClinicalTrials.gov/show/NCT04399980
90. Full Dose Heparin Vs. Prophylactic Or Intermediate Dose Heparin in High Risk COVID-19 Patients. ClinicalTrials.gov. Updated November 22, 2021. Accessed September 17, 2022. https://ClinicalTrials.gov/show/NCT04401293
91. Adaptive COVID-19 Treatment Trial 2 (ACTT-2). ClinicalTrials.gov. Updated March 14, 2022. Accessed September 17, 2022. https://ClinicalTrials.gov/show/NCT04401579
92. Dornase Alfa for ARDS in Patients With Severe Acute Respiratory Syndrome-Coronavirus-2 (SARS-CoV-2). ClinicalTrials.gov. Updated April 14, 2021. Accessed September 17, 2022. https://ClinicalTrials.gov/show/NCT04402970
93. Safety, Tolerability and Efficacy of Molnupiravir (EIDD-2801) to Eliminate Infectious Virus Detection in Persons With COVID-19. ClinicalTrials.gov. Updated February 16, 2022. Accessed September 17, 2022. https://ClinicalTrials.gov/show/NCT04405570
94. Treatment With CSL312 in Adults With Coronavirus Disease 2019 (COVID-19). ClinicalTrials.gov. Updated January 24, 2022. Accessed September 17, 2022. https://ClinicalTrials.gov/show/NCT04409509
95. A Study of LY3819253 (LY-CoV555) in Participants Hospitalized for COVID-19. ClinicalTrials.gov. Updated November 12, 2021. Accessed September 17, 2022. https://ClinicalTrials.gov/show/NCT04411628
96. Study of SOC Plus IVIG Compared to SOC Alone in the Treatment of COVID-19. ClinicalTrials.gov. Updated June 25, 2021. Accessed September 17, 2022. https://ClinicalTrials.gov/show/NCT04411667
97. Study of Sargramostim in Patients With COVID-19. ClinicalTrials.gov. Updated February 18, 2022. Accessed September 17, 2022. https://ClinicalTrials.gov/show/NCT04411680

© 2022 Xiao H et al. *JAMA Intern Med.*

98. Effects of COVID-19 Convalescent Plasma (CCP) on Coronavirus-associated Complications in Hospitalized Patients. ClinicalTrials.gov. Updated August 25, 2021. Accessed September 17, 2022. https://ClinicalTrials.gov/show/NCT04421404
99. Safety and Anti-coronavirus Response of Suppression of Host Nucleotide Synthesis in Patients With COVID-19. ClinicalTrials.gov. Updated February 17, 2022. Accessed September 17, 2022. https://ClinicalTrials.gov/show/NCT04425252
100. A Phase 2 Trial of Infliximab in Coronavirus Disease 2019 (COVID-19). ClinicalTrials.gov. Updated April 20, 2022. Accessed September 17, 2022. https://ClinicalTrials.gov/show/NCT04425538
101. Use of Remote Monitoring for COVID-19 Patient. ClinicalTrials.gov. Updated January 24, 2022. Accessed September 17, 2022. https://ClinicalTrials.gov/show/NCT04425720
102. ANTIBODY-LEVEL BASED ANALYSIS OF COVID-19 CONVALESCENT SERUM (ABACCuS). ClinicalTrials.gov. Updated March 9, 2022. Accessed September 17, 2022. https://ClinicalTrials.gov/show/NCT04432272
103. Crizanlizumab for Treating COVID-19 Vasculopathy. ClinicalTrials.gov. Updated November 9, 2021. Accessed September 17, 2022. https://ClinicalTrials.gov/show/NCT04435184
104. Atovaquone for Treatment of COVID-19. ClinicalTrials.gov. Updated December 14, 2021. Accessed September 17, 2022. https://ClinicalTrials.gov/show/NCT04456153
105. Rapid, Onsite COVID-19 Detection. ClinicalTrials.gov. Updated January 20, 2022. Accessed September 17, 2022. https://ClinicalTrials.gov/show/NCT04460690
106. Adaptive COVID-19 Treatment Trial 3 (ACTT-3). ClinicalTrials.gov. Updated March 14, 2022. Accessed September 17, 2022. https://ClinicalTrials.gov/show/NCT04492475
107. BARCONA: A Study of Effects of Bardoxolone Methyl in Participants With SARS-Corona Virus-2 (COVID-19). ClinicalTrials.gov. Updated January 13, 2022. Accessed September 17, 2022. https://ClinicalTrials.gov/show/NCT04494646
108. A Study to Assess Safety, Tolerability, and Immunogenicity of V591 (COVID-19 Vaccine) in Healthy Participants (V591-001). ClinicalTrials.gov. Updated December 23, 2021. Accessed September 17, 2022. https://ClinicalTrials.gov/show/NCT04498247
109. COVID-19 Positive Outpatient Thrombosis Prevention in Adults Aged 40-80. ClinicalTrials.gov. Updated February 17, 2022. Accessed September 17, 2022. https://ClinicalTrials.gov/show/NCT04498273
110. Study to Evaluate the Efficacy and Safety of Remdesivir (GS-5734,Ñ¢) Treatment of Coronavirus Disease 2019 (COVID-19) in an Outpatient Setting. ClinicalTrials.gov. Updated November 16, 2021. Accessed September 17, 2022. https://ClinicalTrials.gov/show/NCT04501952
111. Treatment of Severe Coronavirus Disease 2019 (COVID-19) With Convalescent Plasma. ClinicalTrials.gov. Updated January 10, 2022. Accessed September 17, 2022. https://ClinicalTrials.gov/show/NCT04502472
112. A Trial to Evaluate Safety and Efficacy of Rivaroxaban (COVID-19). ClinicalTrials.gov. Updated October 22, 2021. Accessed September 17, 2022. https://ClinicalTrials.gov/show/NCT04504032
113. Coronavirus Disease 2019 (COVID-19) Antibody Plasma Research Study in Hospitalized Patients. ClinicalTrials.gov. Updated December 3, 2021. Accessed September 17, 2022. https://ClinicalTrials.gov/show/NCT04524507
114. Dose Ranging Trial to Assess Safety and Immunogenicity of V590 (COVID-19 Vaccine) in Healthy Adults (V590-001). ClinicalTrials.gov. Updated December 29, 2021. Accessed September 17, 2022. https://ClinicalTrials.gov/show/NCT04569786
115. Repeat Testing for SARS-CoV-2. ClinicalTrials.gov. Updated January 20, 2022. Accessed September 17, 2022. https://ClinicalTrials.gov/show/NCT04579549
116. Camostat Efficacy vs. Placebo for Outpatient Treatment of COVID-19 (CAMELOT). ClinicalTrials.gov. Updated January 18, 2022. Accessed September 17, 2022. https://ClinicalTrials.gov/show/NCT04583592
117. Diagnostic Performance of an Antigen Test for SARS-CoV-2 Infection (COVID-19). ClinicalTrials.gov. Updated August 17, 2021. Accessed September 17, 2022. https://ClinicalTrials.gov/show/NCT04610489
118. Electrical Stimulation for Critically Ill Covid-19 Patients. ClinicalTrials.gov. Updated January 21, 2022. Accessed September 17, 2022. https://ClinicalTrials.gov/show/NCT04685213

© 2022 Xiao H et al. *JAMA Intern Med.*

119. A Real World Study of Bamlanivimab in Participants With Mild-to-Moderate Coronavirus Disease 2019 (COVID-19). ClinicalTrials.gov. Updated November 16, 2021. Accessed September 17, 2022. https://ClinicalTrials.gov/show/NCT04701658
120. N-Acetyl Glucosamine as Therapeutic Intervention for Coronavirus Disease-19 (COVID-19). ClinicalTrials.gov. Updated November 4, 2021. Accessed September 17, 2022. https://ClinicalTrials.gov/show/NCT04706416
121. Effects of Sodium Pyruvate Nasal Spray in COVID-19 Long Haulers. ClinicalTrials.gov. Updated March 9, 2022. Accessed September 17, 2022. https://ClinicalTrials.gov/show/NCT04871815
122. BinaxNOW for Evaluating Children for Infection With SARS-CoV-2 (COVID-19). ClinicalTrials.gov. Updated January 28, 2022. Accessed September 17, 2022. https://ClinicalTrials.gov/show/NCT05087524

© 2022 Xiao H et al. *JAMA Intern Med.*
